# Genome-wide effects of social status on DNA methylation in the brain of a cichlid fish, *Astatotilapia burtoni*

**DOI:** 10.1186/s12864-019-6047-9

**Published:** 2019-09-11

**Authors:** Austin T. Hilliard, Dan Xie, Zhihai Ma, Michael P. Snyder, Russell D. Fernald

**Affiliations:** 1 Department of Biology, Stanford, CA 94305 USA; 2 Department of Genetics, Stanford University School of Medicine, Stanford, CA 94305 USA

**Keywords:** DNA methylation, social status, cichlid fish, *Astatotilapia burtoni*, brain, hypothalamus, neural plasticity, gene regulation, bisulfite sequencing, environment, whole genome

## Abstract

**Background:**

Successful social behavior requires real-time integration of information about the environment, internal physiology, and past experience. The molecular substrates of this integration are poorly understood, but likely modulate neural plasticity and gene regulation. In the cichlid fish species *Astatotilapia burtoni*, male social status can shift rapidly depending on the environment, causing fast behavioral modifications and a cascade of changes in gene transcription, the brain, and the reproductive system. These changes can be permanent but are also reversible, implying the involvement of a robust but flexible mechanism that regulates plasticity based on internal and external conditions. One candidate mechanism is DNA methylation, which has been linked to social behavior in many species, including *A. burtoni*. But, the extent of its effects after *A. burtoni* social change were previously unknown.

**Results:**

We performed the first genome-wide search for DNA methylation patterns associated with social status in the brains of male *A. burtoni*, identifying hundreds of Differentially Methylated genomic Regions (DMRs) in dominant versus non-dominant fish. Most DMRs were inside genes supporting neural development, synapse function, and other processes relevant to neural plasticity, and DMRs could affect gene expression in multiple ways. DMR genes were more likely to be transcription factors, have a duplicate elsewhere in the genome, have an anti-sense lncRNA, and have more splice variants than other genes. Dozens of genes had multiple DMRs that were often seemingly positioned to regulate specific splice variants.

**Conclusions:**

Our results revealed genome-wide effects of *A. burtoni* social status on DNA methylation in the brain and strongly suggest a role for methylation in modulating plasticity across multiple biological levels. They also suggest many novel hypotheses to address in mechanistic follow-up studies, and will be a rich resource for identifying the relationships between behavioral, neural, and transcriptional plasticity in the context of social status.

**Electronic supplementary material:**

The online version of this article (10.1186/s12864-019-6047-9) contains supplementary material, which is available to authorized users.

## Background

Social interactions regulate individual access to resources like food and potential mates that are critical for survival and reproduction [1], and social relationships are essential for well-being in many species, including humans [2, 3]. Acting optimally in social contexts requires integration of information about the external environment, internal physiology, and past experience in real-time to modify behavior [4, 5]. The molecular substrates underlying this integration are complex and not well understood [6], but rapid responses to changing circumstances depend on flexible brain function, i.e. neural plasticity, which in turn is supported by context-specific patterns of gene transcription [7]. Therefore, knowing when and where to act is contingent on plasticity at multiple levels, from gene transcription through neurons and neural circuits to behavior. This idea is supported by evidence that different behaviors depend on specific combinations of transcriptional states in key brain regions that are dynamically regulated in response to changes both in the environment and body [8–13]. A leading candidate mechanism for achieving these transcriptional dynamics is DNA methylation [7, 14–16].

DNA methylation can mediate between the environment and the genome via the dynamic addition and removal of epigenetic marks that alter gene expression [17]. It has been implicated in multiple forms of neural plasticity, and active (de) methylation is an important regulator of gene expression in adult neurons involved in learning and memory [18–20]. Methylation likely supports “metaplasticity” (plasticity *of* neural plasticity), since it is a relatively stable epigenetic mark that also retains the potential for change [21, 22]. For example, methylation altered by learning could flag genes for regulation only in certain conditions, priming the future activation of specific transcriptional states without disrupting baseline neural function by permanently altering synapses or cell homeostasis [22]. The idea of metaplasticity extends naturally to behavioral flexibility as well, and changes in methylation have been associated with social behavior and status in humans [23, 24], non-human primates [25], rodents [26–28], insects [14, 29], and a well-studied cichlid fish, *Astatotilapia burtoni* (Additional file 1: Figure S1) [30], which was the focus of this study.

Male *A. burtoni* exist in a dynamic hierarchy where an individual’s social standing can shift rapidly depending on the social environment, causing fast behavioral modifications that trigger a cascade of changes in gene transcription, the hypothalamus, and the reproductive system [31–33]. Here, we studied DNA methylation in brains of male *A. burtoni* from the extreme ends of the social spectrum: stable dominant (D) males that control territory - and thus access to food and potential mates - and socially suppressed non-dominant (ND) males. D males are reproductively capable and employ a repertoire of behaviors to aggressively maintain territory and court and spawn with females [34], while ND males are reproductively incapable and their behavior is mostly limited to fleeing from aggressors. D males have gonadotropin-releasing hormone (GnRH1) neurons in the preoptic area (POA) of the hypothalamus that are roughly eightfold larger in volume [35, 36], a more active hypothalamic-pituitary-gonadal (HPG) axis [37], and larger testes compared to ND males [38, 39].

However, ND males will display dominance behaviors almost immediately when given the social opportunity, and this “social ascent” is accompanied within minutes by increased GnRH1 neuron activity [40] and steroid hormone production [41, 42], as well as transcriptional activation across multiple brain regions and the HPG axis [33, 37, 43]. Over the next few days after ascent, sustained dominance leads to increased cell proliferation and neural differentiation in the hypothalamus [44]. GnRH1 neuron size and dendritic complexity also increase [35, 36, 45, 46], altering the firing properties and neuronal connections of these cells [47, 48] and leading to rapid testes growth and sperm production [49].

Remarkably, all of these changes are reversible, and male *A. burtoni* can move up and down the social hierarchy multiple times throughout life. The stable-yet-reversible nature of *A. burtoni* social status resembles a form of metaplasticity whereby changes in neural function and behavior are dynamically modified depending on social context and internal state, suggesting that DNA methylation may bridge plasticity at the transcriptional, cellular, and behavioral levels. Indeed, systemic manipulation of methylation can promote or inhibit *A. burtoni* social ascent [30], but how it affects biological function in brain areas like the hypothalamus that are deeply intertwined with social status remains unknown. Here, we hypothesized that genome-wide signatures of methylation could reveal key biological processes driven by social change. In particular, we predicted that methylation would affect genes germane to both synaptic and homeostatic neural plasticity since 1) past experience guides behaviors of both D and ND males in key social contexts [50–53], and 2) the profound growth of cells in the POA after social ascent - including GnRH1 and somatostatin neurons [54] - is likely accompanied by changes in the distribution of neurotransmitter receptors and ion channels.

We performed whole-genome bisulfite sequencing (BS-seq) and RNA-seq on brain tissue from two males of each social status. Since methylation levels are correlated across the genome and functionally relevant differences are generally associated with entire regions as opposed to single loci, we used the BSmooth method to search for differentially methylated regions (DMRs) [55], identifying hundreds across the genome. Because this approach was novel in *A. burtoni,* we measured distances between DMRs and genes and devised a method to determine the significance of the results (see Methods). Roughly 75% of the DMRs were in a gene body and most of the rest were within 5 kb of a gene. These “DMR genes” were involved in processes supporting neural plasticity, development, and growth, and a few were differentially expressed, although the overall relationship between methylation and expression varied depending on gene function and DMR location. This was also true of the relationships between methylation, transposable elements (TEs), and conserved non-coding elements (CNEs), and a significant number of DMRs overlapped long non-coding RNAs (lncRNAs) that were antisense to a protein-coding gene, hinting at subtle interactions between multiple layers of gene regulation. Our results generated many novel hypotheses to address in mechanistic follow-up studies, and will be a rich resource for identifying the relationships between behavioral, neural, and transcriptional plasticity in the context of social status.

## Results

**Fig. 1:**
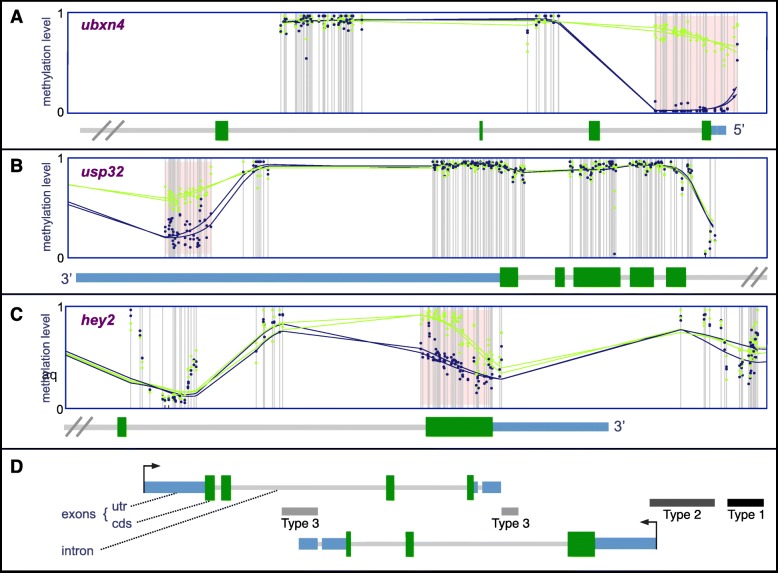
Example DMRs with schematic illustration of regulatory region DMR types. **a-c:** Raw methylation ratios (colored dots) and smoothed methylation values (colored lines, y-axis) for CpGs with more than 4x coverage (grey vertical lines) in every fish are colored by social state (green = dominant, blue = nondominant), while the relevant parts of the genome are schematized below following (**d**), including non-coding and protein-coding exons, introns, and 5′ or 3′ ends (x-axis). Pink shading shows the extent of each DMR. As illustrated, (**a**), UBX domain protein 4 (*ubxn4*) had a type-2 regulatory region DMR that overlapped its transcription start site; (**b**), ubiquitin specific peptidase 32 (*usp32*) had a type-2 regulatory region DMR within its 3′ untranslated region; and (**c**), hes-related family bHLH transcription factor with YRPW motif 2 (*hey2*) had a DMR that extended from its last intron to cover its last protein-coding exon. **d:** We classified three different types of DMRs in regulatory regions schematized as shown in a hypothetical genomic region with two genes in antisense orientation. **Type-1**: within 5 kb of a gene but outside the body. **Type-2**: overlapping any of the first or last 1 kb of a gene. **Type-3**: a type-1 or type-2 DMR for one gene that was also within the body of another gene on the opposite strand

**Fig. 2:**
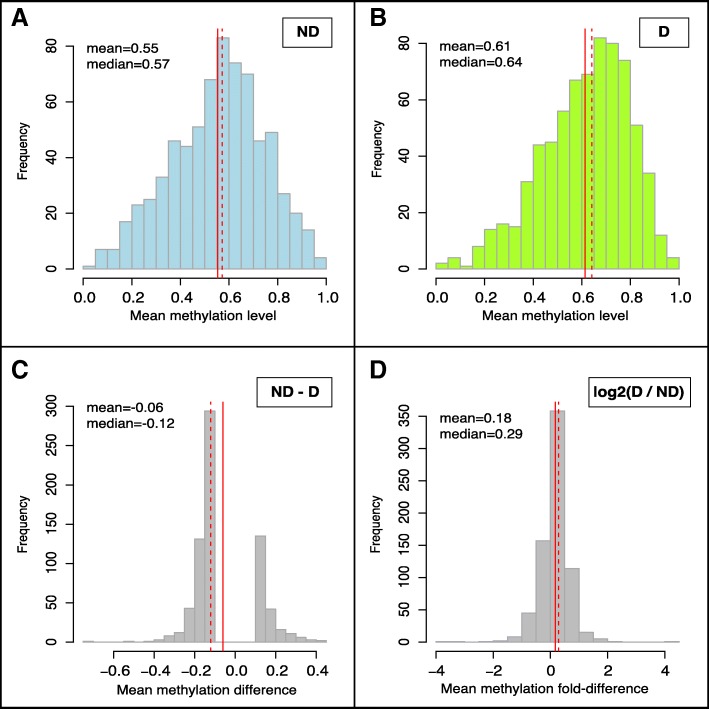
Mean methylation levels and differences across all DMRs. Histograms of average methylation levels within all 709 DMRs, and how they were different across social status. Means and medians of each distribution are reported in the plot and represented by vertical solid and dashed red lines, respectively. All values for individual DMRs are reported in Additional file 2: Table S1. **a-b:** Averages of smoothed methylation levels across all cytosines within each DMR for ND (**a**) and D (**b**) fish. **c:** Differences between average smoothed methylation levels for ND - D fish, such that negative values reflect higher methylation in D fish. DMRs where the absolute value of this difference was less than 0.1 were filtered out during quality control (Methods). **d:** Log2 ratio (fold-difference) of average smoothed methylation values for D / ND fish, such that positive values reflect higher methylation in D fish

We identified 709 DMRs in *A. burtoni* hypothalamus as a function of social status. Three of the most significant are schematized in Fig. 1 (see Methods). The median DMR was 330 base pairs (bp) long, with average baseline methylation levels of 60% that differed by 26% between D and ND males (Fig. 2, Additional file 1: Figure S2; Additional file 2: Table S1A). D and ND methylation was effectively identical across the > 99.9% of the genome outside of the DMRs (Additional file 1: Figure S3). D males had higher methylation levels in 494 DMRs (D-DMRs) and 12% higher methylation in the DMRs overall, even including the 215 where ND methylation was higher (ND-DMRs, *p* < 7.8e-10, Mann-Whitney *U* test). D- and ND-DMRs varied in a number of ways but the biological significance of these differences was unclear (see Methods, Additional file 1: Figure S4).

### DMR locations and characteristics relative to genes and other genomic features

Altogether, > 90% of the DMRs overlapped a gene, regulatory region (defined as 5 kb up- to 1 kb downstream of the 5′ end, or 1 kb up- to 5 kb downstream of the 3′ end), TE, or CNE (Additional file 2: Table S1B-C). DMRs were closer to more genes than expected by chance, especially within 15 kb (Additional file 1: Figure S5, Figure S6A), and methylation differences were greater in areas with more genes, independent of other DMR characteristics (Additional file 1: Figure S6B-C). A significant majority of DMRs were in a gene body or regulatory region (*n* = 626, Table 1A, Additional file 1: Figure S7A), and a significant number of DMRs in genes and 5′ regulatory regions contained a CNE (Table 1B). In contrast, there were significantly fewer overlaps between DMRs and TEs than expected by chance, particularly in gene bodies (Table 1B). Some intergenic DMRs contained a TE (*n* = 13) or CNE (*n* = 10; Additional file 1: Figure S7B), and all others were within 5 kb of a TE (*n* = 60, median = 0.8 kb distance).

**Table 1:** Significance of DMRs overlapping genomic features

A	body	p-val	5′ reg	p-val	3′ reg	p-val
** DMRs**	522	**<1e-4**	147	**0.027**	145	**0.042**
**> 1 gene**	15	0.15	20	0.46	19	0.61
** genes**	484	**<1e-4**	166	**0.057**	162	0.11
**> 1 DMR**	42	**0.042**	2	0.47	5	**0.053**
**coding**	469	**<1e-4**	150	0.12	156	**0.053**
**lncRNA**	15	0.43	16	**0.055**	6	***0.94***
B	TE	<null p-val	CNE	>null p-val		
** DMRs**	111	**<1e-4**	44	**<1e-4**		
** DMRs (> 1 hit)**	29	**0.006**	3	0.29		
** hits**	146	**<1e-4**	48	**0.001**		
**+gene body**	65	**<1e-4**	28	**<1e-4**		
**+ 5′ region**	34	0.46	6	**0.0093**		
**+ 3′ region**	33	0.34	3	0.28		

A: Numbers of DMRs in gene bodies and regulatory regions with p-values (columns). Rows are the numbers of: DMRs overlapping at least one (1) or more (2) genes, genes with at least one (3) or more (4) DMRs, protein-coding genes (5), and long-non-coding RNAs (6). P-values are defined as the fraction of nullDMR sets with as many or more overlaps as the real DMRs and p-values < 0.1 are bolded (see Methods). The 3′ regulatory region-lncRNA p-value is italicized because this number was actually lower than expected since only ~ 6% of the nullDMR sets had fewer hits in 3′ regulatory regions of lncRNAs

B: Numbers of DMRs containing transposable elements (TEs) and conserved non-coding elements (CNEs) with p-values (columns). Rows are the numbers of: DMRs overlapping at least one (1) or more (2) TE/CNE, TE/CNEs in a DMR (3), TE/CNEs in a DMR that also overlapped a gene body (4) or regulatory region (5,6). *P*-values reflect the fraction of nullDMR sets with fewer (TEs) or more (CNEs) hits than the actual DMRs

**Fig. 3:**
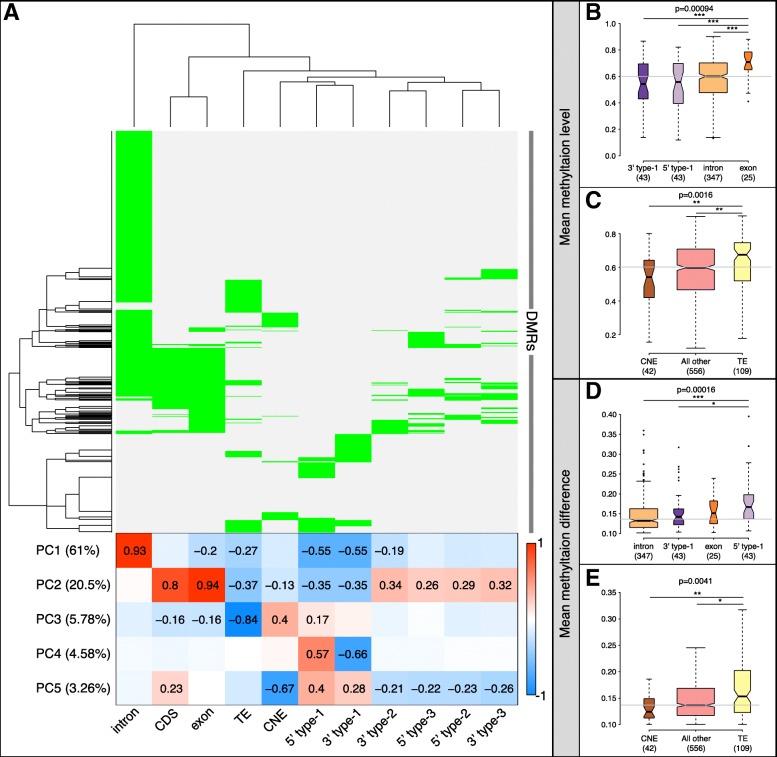
DMRs overlapping different genomic features had distinct methylation profiles. **a:** Upper heatmap and dendrograms show unsupervised hierarchical clustering of 709 DMRs (rows) based on the combinations of genomic features they overlapped (columns). Green colored cells indicate DMR-feature overlap. Bottom heatmap shows Pearson correlations between the first 5 principal components (PCs, rows) of the DMR correlation matrix and genomic features (columns). The name of each PC is followed in parentheses by the amount of variance it explained within the DMR correlations. Red and blue denote strong positive and negative correlations, respectively. Correlation coefficients are shown in cells where the *p*-value was significant after Bonferroni-correction for 55 tests (5 PCs × 11 feature types). **b-e:** Boxplots show comparisons of DMR baseline methylation levels (**b-c**, y-axis) and the absolute value of ND - D methylation differences (**d-e**, y-axis) within different groupings of the DMRs. Baseline methylation is defined as the mean of all smoothed methylation values within the DMR across all fish and denoted as all.mean in Additional file 1: Figure S2 and Additional file 2: Table S1, while the absolute value of ND - D methylation differences is denoted as meanDiff.abs in Additional file 1: Figure S2 and Additional file 2: Table S1. First, DMRs are grouped by whether they were contained within one part of a gene body or regulatory region, including introns, exons, within 5 kb upstream of transcription start site (5′ type-1), or within 5 kb downstream of 3′ end (3′ type-1) (**b**, **d**), and second, DMRs were grouped by whether they contained transposable elements (TEs) or conserved non-coding elements (CNEs) (**c**, **e**). The number of DMRs in each grouping is reported in parentheses below group names in each boxplot. Horizontal grey lines denote the median value across all DMRs tested. Kruskal-Wallis omnibus *p*-values are reported above each plot and asterisks indicate significance levels of pairwise comparisons after Dunn’s test with Bonferroni-correction; *p* < 0.05*, 0.01**, 0.001***

Almost half of the DMRs overlapped more than one genomic feature (*n* = 307, Fig. 3a top), but roughly 80% of the variability in DMR location could be attributed to whether they were located in a gene body, and if so, whether they overlapped an intron or exon (Fig. 3a bottom, PC1–2). Other criteria were whether DMRs overlapped a TE or CNE, and for those within 5 kb of a gene, whether they were up- or downstream of it (Fig. 3a bottom, PC3–5).

Many DMRs in regulatory regions had the potential to affect multiple genes. Some were in both the body of one gene and regulatory region of another, so we divided regulatory region DMRs into 3 types for further analysis (Fig. 1d, Additional file 1: Figure S7B-D). Type-1: within 5 kb of a gene but outside the body (*n* = 104). Type-2: overlapping any of the first or last 1 kb of a gene (*n* = 71). Type-3: a type-1 or type-2 DMR for one gene that was also within the body of another gene on the opposite strand (*n* = 110). Many type-1 DMRs in the 5′ region of one gene were also 3′ to another gene (Table 2, Additional file 1: Figure S7B-C). There was also significant overlap between type-2 and type-3 DMRs (Table 2A, Additional file 1: Figure S7D), i.e. DMRs that overlapped the first or last 1 kb of a gene were likely also within the body of another gene on the antisense strand. Finally, it was more common for 5′ versus 3′ type-2 DMRs to overlap an intron (OR = 2.34, *p* < 0.02, Fisher’s exact test; Additional file 1: Figure S7C), and many intronic DMRs overlapped protein-coding (OR = 2.74, *p* < 4.85e-4), but not non-coding exons (OR = 0.131, *p* < 1.43e-8), thus intronic DMRs tended to be well within gene bodies and away from the 3′ end.

**Table 2:** Regulatory region DMRs were often linked to multiple genes and had little relationship to TEs

A: DMRs	5' type-3	3' type-2	3' type-3	5' type-1
** 5' type-2**	**7.04** **<2e-6**	2.31 >0.12	**11.6** **<7e-11**	
** 5' type-3**		**9.06** **<4e-7**	2.69 >0.01	
** 3' type-2**			**6.97** **<8e-6**	
** 3' type-1**				**5.87** **<5e-7**
B: Genes	DMR			
	body	5’ reg	3’ reg	
** TE**				
** body**	**3.28** **<4e-22**	1.12 >0.54	1.16 >0.44	
** 5’ reg** ** +body**	**1.46** **<6e-5**	0.97 >0.87	1.28 >0.13	
** 5’ reg** ** -body**	**0.29** **<5e-15**	0.9 >0.68	0.83 >0.41	
** 3’ reg** ** +body**	**1.94** **<6e-12**	1.28 >0.13	1.02 >0.93	
** 3’ reg** ** -body**	**0.33** **<4e-13**	0.94 >0.83	0.94 >0.83	

In both A and B, cells contain odds-ratios (top) and uncorrected p-values (bottom) from Fisher’s exact test, and highly significant results (p<6e-5) are in bold. A: Significance of overlaps between different types of DMR-regulatory region hits (see Figure 1d). **Type-1**: within 5kb of a gene but outside the body. **Type-2**: overlapping any of the first or last 1kb of a gene. **Type-3**: a type-1 or type-2 DMR for one gene that was also within the body of another gene on the opposite strand. Empty cells are either a redundant or undefined comparison, e.g. by definition 5’ and 3’ type-1 DMRs cannot overlap any part of a gene body so there were no overlaps with type-2/3. B: TEs in gene bodies and regulatory regions were predictive of DMRs in gene bodies but not regulatory regions. Significance of overlaps between genes based on whether they had a DMR in their body or regulatory region (columns) and a TE in their body, regulatory region and body (+body), or regulatory region and not body (-body, rows)

DMRs had different relationships with TEs depending on their location relative to genes. The number of regulatory region DMRs with a TE was unremarkable overall (Table 1B), but a disproportionate amount of type-1 DMRs overlapped a TE (5′ and 3′: OR = 3.5, *p* < 6e-5). In contrast, gene body DMRs with a TE were rare overall (Table 1B), and they were also underrepresented within this small number of DMR-TE-gene overlaps (OR = 0.44, *p* < 2e-4). Notably, a TE anywhere within 5 kb of a gene was informative about differential methylation in the gene body, regardless of whether it overlapped a DMR; genes were more likely to have a DMR in their body if there was also a TE there, but less likely if they only had a TE in either regulatory region (Table 2B).

Some DMR characteristics varied by the type of feature(s) they overlapped. For example, DMRs that overlapped a TE had relatively low GC content (*p* < 0.003, Mann-Whitney), as well as greater methylation levels (Fig. 3c) and differences across social status (Fig. 3e), especially compared to DMRs with a CNE. There was little correlation overall between DMR methylation levels and how different they were across status (r = − 0.062, *p* < 0.1). We compared DMRs that fell within a single intron, exon, or regulatory region (*n* = 458, Additional file 1: Figure S7C) and found that type-1 5′ DMRs and exonic DMRs had larger differences than those in 3′ regions or introns (Fig. 3d), even though baseline methylation was significantly higher in exons (Fig. 3b). DMRs in coding and non-coding exons had similar methylation levels and differences across status, even though coding exons had higher GC content (methylation difference *p* > 0.28, level *p* > 0.78, GC *p* < 2e-4).

Some DMR-gene relationships were not one-to-one; 42 genes had > 1 DMR in their body and 15 DMRs overlapped > 1 gene body (Table 1A, Additional file 2: Table S2). Genes with > 1 DMR had more splice variants than single-DMR genes (*p* < 0.001, Mann-Whitney). Multi-gene DMRs were more likely to overlap transcription start sites (OR = 16.3, *p* < 6e-5) and lncRNAs (OR = 64.2, *p* < 7e-8), and a significant portion of the genes they overlapped were in fact lncRNAs (OR = 16.7, *p* < 8e-6). Therefore, even though only 3% of the gene bodies overlapped by a DMR were lncRNAs (*n* = 15), nearly half of them were antisense to a protein-coding gene (*n* = 7), and this lncRNA-coding pairing made up about half of all instances where a DMR overlapped two genes. All the protein-coding genes in these pairs were expressed less in D fish. DMR genes in coding-coding pairs were not biased towards either status, but their expression levels were more correlated (r = − 0.7, *p* < 0.09) than those in coding-lncRNA pairs (r = − 0.16, *p* < 0.8).

### Relationships between gene expression, methylation, and DMR location

The same tissue samples used to assay DNA methylation were also processed for RNA-seq so that gene expression and methylation could be directly compared (see Methods). Baseline gene expression was defined as the average level across all fish, and fold-difference across social status as log2(D/ND) expression. Seven genes that were significantly differentially expressed across social status had a DMR in their body, regulatory region (for example *ubxn4*, see Fig. 1a), or within 40 kb upstream (see Methods, Additional file 2: Table S3). Across all DMR genes, the relationships between expression and methylation varied by which part(s) of genes were differentially methylated.

**Fig. 4:**
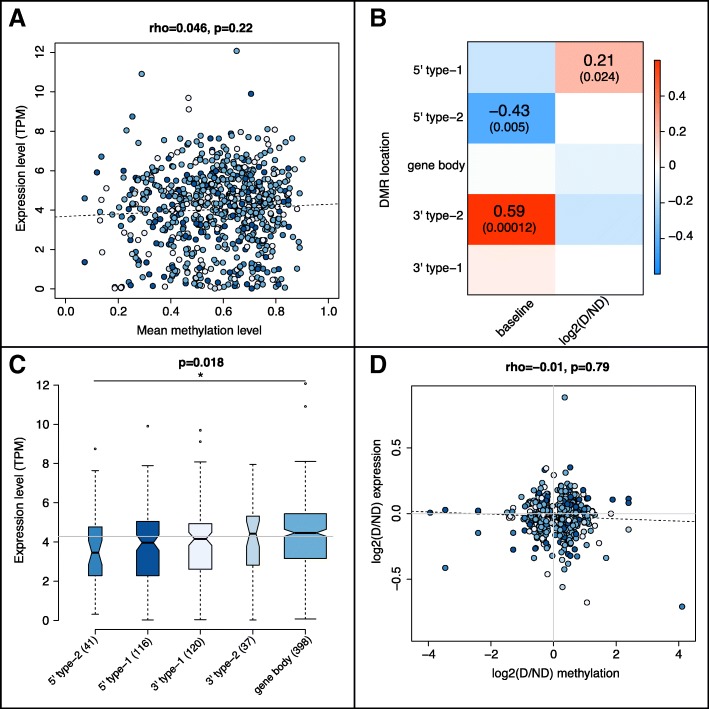
Relationships between DNA methylation and gene expression in DMR genes. **a:** Mean gene expression levels (TPM: transcripts per million, y-axis) across all fish for each DMR gene (dots), plotted as a function of the mean methylation level across all fish (x-axis) for each gene’s DMR(s). **b:** Heatmap representing Spearman correlations between methylation and gene expression as a function of DMR location (rows). Each row corresponds to a subset of genes with the same color in **a-c**. Column 1 shows correlations between baseline (mean) gene expression (TPM) and DMR methylation levels (all.mean from Additional file 1: Figure S2 and Additional file 2: Table S1), corresponding to subsets of genes from **a**. Column 2 shows correlations between expression and DMR fold-differences (see Fig. 2d and Additional file 2: Table S1), corresponding to subsets of genes from **d**. Darker red and blue colors in cells denote stronger positive and negative correlations, respectively, as shown in legend to right of the heatmap. Spearman’s rho values are shown in cells with p-values < 0.03. **c:** Boxplot comparing mean gene expression levels across all fish (y-axis) for DMR genes, based on whether DMRs were in the body and/or 5′ or 3′ regulatory regions (x-axis). Omnibus p-value from a Kruskal-Wallis test is reported above the plot, and group sizes are reported in parentheses after the group name under each box. Top and bottom of boxes represent the first and third quartiles, respectively, whiskers extend to the most extreme data points no more than 1.5 times the interquartile range from the box. Dots show data points beyond this range, and horizontal grey line denotes the overall median. *p < 0.05 from Dunn’s post-hoc test with BH-correction. **c:** Scatterplot of gene expression fold-difference, defined as log2 of D/ND (y-axis), as a function of methylation fold-difference (x-axis) for DMR genes (dots). The same genes are represented in all panels (*n* = 712). Genes with a DMR in both the 5′ and 3′ regulatory regions are not shown (*n* = 4). In **a**, **c**, **d**, methylation levels were averaged across all DMRs for genes with > 1 DMR in their body and/or regulatory region(s) (*n* = 58). In **a** and **d**, dashed lines represent the best-fit linear regression, Spearman’s rho is reported above the plot, and gene colors match the color of their corresponding group in **c**

Baseline gene expression and DMR methylation levels were not correlated overall (Fig. 4a), but they were in genes with type-2 regulatory region DMRs: negatively when the DMR was on the 5′ end, and positively when it was on the 3′ end (Fig. 4b). That is, when average methylation levels across all fish were high within a DMR in the first 1 kb of a gene then average expression levels were low, but vice versa when the DMR was in the last 1 kb of a gene. In contrast, there was no correlation between baseline DMR methylation and gene expression when DMRs were only in the gene body (Fig. 4b), but these genes had more splice variants than those with regulatory region DMRs (Additional file 1: Figure S8B), suggesting that methylation could be controlling expression of specific transcript isoforms in these cases. Finally, genes with a DMR in their body also had higher baseline expression than those with a 5′ regulatory region DMR (Fig. 4c).

Similar to baseline levels, there was no correlation overall between methylation and expression fold-differences across social status (Fig. 4d), or in genes with just a body DMR (Fig. 4b). There was however a mild positive correlation among genes with a 5′ type-1 DMR (rho = 0.21, *p* = 0.024; Fig. 4b), i.e. the magnitudes of methylation and expression differences were only linked when the DMR was in the first 1 kb of the gene. Genes where DMR methylation was higher in one status but gene expression was lower, or vice versa, were more likely to have type-2 DMRs (5′: OR = 3.8, *p* < 2e-3; 3′: OR = 2.7, *p* < 0.04) where methylation was higher in D fish (D-DMRs: OR = 2.4, *p* < 1e-5), but when methylation and expression were both higher or lower in one status, gene expression tended to be higher in D fish (Additional file 1: Figure S8C).

### Properties of DMR genes and connections with other regulatory mechanisms

Genes with a DMR in their body and/or regulatory region differed from genes that lacked a DMR in a number of ways: they had significantly higher baseline expression, greater expression differences across social status, higher GC content, more splice variants, and contained more TEs than other genes (Additional file 1: Figure S9). DMR genes were also more likely to be transcription factors (OR = 2.1, *p* < 2e-7), overlap a lncRNA (OR = 1.8, *p* < 9e-4, Additional file 2: Table S4), or have at least one duplicate elsewhere in the genome (OR = 1.8, *p* < 2e-6). Together, these findings indicate that genes with differential methylation are more constitutively active, and that their expression is shaped by a more diverse set of regulatory mechanisms than other genes.

Next, we asked whether transcription factor, lncRNA-overlapping, or duplicated DMR genes differed from other DMR genes. We found that transcription factor DMR genes had relatively low baseline expression (*p* < 7e-4, Mann-Whitney) and methylation levels (*p* < 4e-7) compared to other DMR genes, and that their DMRs were more likely to contain a CNE (OR = 4.5, *p* < 6e-5), suggesting the presence of conserved regulatory elements. Also, significant numbers of lncRNA-overlapping DMR genes had a D-DMR and/or > 1 DMR (both OR > 2.4, *p* < 0.05). For comparison, the number of DMR genes that overlapped another protein-coding gene was unremarkable (OR = 0.84, *p* > 0.16) and no more than expected had D-DMRs or > 1 DMR (0.7 < OR < 0.9, *p* > 0.7). Finally, not only were a significant amount of DMR genes duplicates, there were a number of cases where more than one copy of a duplicated gene had a DMR (see Additional file 2: Table S1B).

These different subtypes of DMR genes were also enriched for distinct but related biological processes and functions (BY-adjusted *p* < 0.1, Additional file 1: Figure S10A-C), commensurate with those enriched in the DMR genes overall (described next).

### DMR genes were involved in neural development, synapse function, and signaling

**Fig. 5:**
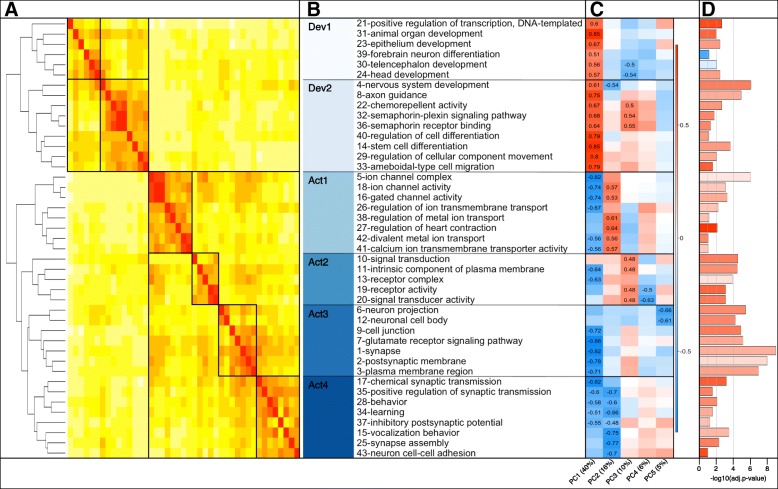
DMR genes were enriched for GO terms related to neural development and activity. **a:** Heatmap of pairwise Pearson correlations between GO terms (rows and columns) and dendrogram showing the results of unsupervised hierarchical clustering. Each GO term was represented by a vector of all DMR genes where values of 1 or 0 indicated the presence or absence, respectively, of a given gene in the term, then these vectors were used to compute correlations. Clusters of GO terms that shared many of the same DMR genes are denoted by black boxes. See Additional file 2: Table S5A for all DMR genes in each GO term. **b:** Names of enriched GO terms (rows), preceded by their significance rank, e.g. 1-synapse was the most significant. Different shades of blue to the left denote clusters and sub-clusters of GO terms, broadly defined as related to development (Dev) or activity (Act). The same color-cluster mappings are used in Fig. 6 and Additional file 1: Figure S10C and Figure S13. **c:** Heatmap of Pearson correlations between the first five principal components (PCs) of the correlation matrix in **a** (columns) and GO terms, represented by gene vectors as in **a** (rows). The name of each PC is followed in parentheses by the amount of variance it explained within the GO term correlations. Red and blue denote strong positive and negative correlations, respectively. Values are shown for correlations significant after Bonferroni correction for 215 tests (5 PCs × 43 GO terms). **d:** Bars plot -log10(Benjamini-Yekutieli-adjusted p-values) for each term. Vertical grey lines represent p-values of 0.05, 0.01, 1e-4. Bar color represents the average DMR log2(D/ND) methylation fold-difference for genes in the term, where darker red or blue represent higher mean methylation levels in D or ND fish, respectively. This quantity is referred to as log2fc in Additional file 1: Figure S2 and Additional file 2: Table S1

**Fig. 6:**
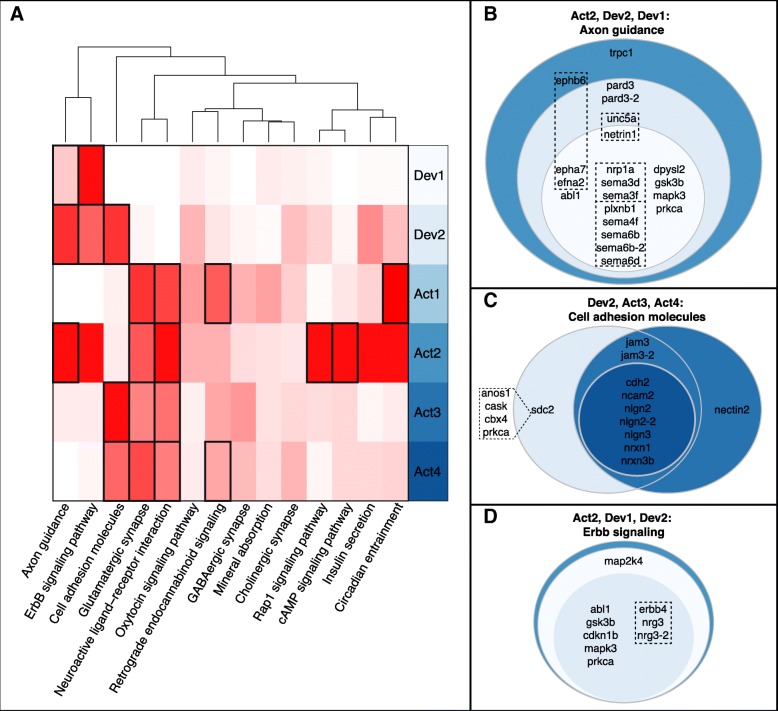
Molecular pathways distinguish types of DMR gene GO term clusters. **a:** Heatmap of odds-ratios (OR) from Fisher’s exact tests comparing the amount of overlap between DMR genes in enriched GO term clusters (rows) and KEGG pathways (columns). White represents OR~ 1 and darker shades of red indicate higher values, and thus more overlap than expected. Black boxes highlight comparisons that passed Bonferroni-correction for 84 tests (14 KEGG pathways × 6 GO term clusters). For reference, the overlap between Dev1 and Axon guidance (leftmost cell in top row) was the least significant of these with OR > 5.8 and corrected *p* < 0.023. Dendrogram above shows the results of unsupervised hierarchical clustering of the KEGG pathways based on the values in the heatmap. Complete lists of all DMR genes in each KEGG pathway can be found in Additional file 2: Table S5B. **b-d:** Venn diagrams showing DMR genes in the Axon guidance (**b**), Cell adhesion (**c**), and ErbB signaling (**d**) KEGG pathways, divided by their presence in one or more GO term clusters. Colors match those behind term cluster names in **a**. Dashed boxes in **b** and **d** highlight receptor-ligand interactions and the dashed polygon in **c** contains four DMR genes of interest that were not in the Cell adhesion pathway, but interact with *sdc2*, which was. *pard3* and *sema6b* (**b**), *jam3* and *nlgn2* (**c**), and *nrg3* (**d**) had at least one other copy in the genome that also had a DMR (see Additional file 2: Table S1B)

To investigate the potential biological effects of differential methylation, we performed functional enrichment analyses with the DMR genes (see Methods), finding they were enriched in 43 GO categories, (BY-adjusted *p* < 0.1; Fig. 5; Additional file 2: Table S5A) and 14 KEGG pathways (BH-adjusted *p* < 0.1; Fig. 6; Additional file 2: Table S5B). Prominent themes among these results were neural development, synapse function, and signaling, including many molecules in glutamatergic and GABAergic synapses and axon guidance pathways (Additional file 1: Figure S11, Figure S12).

Thirty-five DMR genes were each in 15–20 of the enriched GO terms and/or at least 3 KEGG pathways (Additional file 2: Table S5C), which led us to cluster terms by their constituent genes and systematically look for overarching functional patterns (Fig. 5). There were two distinct groups of GO terms broadly related to: 1) development and cell differentiation (Dev), and 2) neural structure and activity (Act). In turn, these could be broken into sub-clusters of related GO terms (Dev1–2, Act1–4), and six DMR genes were in at least one term from every cluster (*abl1, gabra5, gabrb3, gnao1(102305147), shank3, slc8a3*). See Methods section on genome annotation for explanation of the gene naming conventions in this paper.

Some properties of genes in the Dev and Act GO term groups were significantly different, including average gene size, GC content, TE content, baseline methylation and expression levels, and whether methylation and expression fold-differences went in the same direction across social status (Additional file 1: Figure S13). Dev and Act terms were also differentiated by the high numbers of transcription factor genes in the Dev1 and Dev2 clusters (both OR > 4.3, *p* < 3e-7), including several involved in hormone signaling (Additional file 1: Figure S10C), compared to significantly few in Act1, Act3, and Act4 (all OR < 0.3, *p* < 0.03).

Dev GO terms were uniquely enriched for gene markers overexpressed in specific cell types from [56]: endothelial cells (epithelium development-Dev1, stem cell differentiation-Dev2; OR > 5.7, BH-adjusted *p* < 0.04), and newly-formed oligodendrocytes (chemorepellent activity, semaphorin-plexin signaling pathway, semaphorin receptor binding-all Dev2; all OR > 56, *p* < 0.04) (see Methods; Additional file 2: Table S6). In general, GO terms were composed of genes with higher DMR methylation levels in D fish, but two Dev1 terms were the only ones where levels were higher in ND fish (telencephalon development, forebrain neuron differentiation, Fig. 5d).

Three Act GO terms were uniquely enriched for genes with DMRs that overlapped a TE (intrinsic component of plasma membrane, signal transducer activity, receptor activity; all OR > 2.7, BH-adjusted *p* < 0.05). In general, grouping DMR genes by GO term revealed correlations between the number of TEs in gene bodies and various gene/DMR characteristics (Additional file 1: Figure S14), suggesting a functionally important relationship between TEs and methylation.

Finally, although the Dev and Act GO term clusters were distinct there was meaningful overlap between some of their subgroups. Dev1 and Act2 shared a significant number of genes, as did Dev2 and Act2–4 (Table 3), and Dev2, Act1, and Act4 were all enriched for gene markers overexpressed in neurons (all OR > 2.5, *p* < 3e-3). Comparing genes in the GO term clusters to those in enriched KEGG pathways, much of the overlap between the Dev1/Dev2 and Act2 clusters was due to genes involved in axon guidance and ErbB signaling, and overlap between the Dev2 and Act3/Act4 clusters was due to cell adhesion molecules (Fig. 6).

**Table 3:** Overlaps between different enriched GO term clusters

	Dev1	Dev2	Act1	Act2	Act3	Act4
**Dev1**	–	106	23	**105**	50	23
**Dev2**	<2e-24	–	28	**110**	**67**	**40**
**Act1**	0.78	0.063	–	55	45	31
**Act2**	**<4e-5**	**<3e-9**	<2e-10	–	97	48
**Act3**	0.92	**<4e-5**	<3e-13	<2e-7	–	46
**Act4**	0.82	**<4e-6**	<5e-13	<4e-4	<4e-12	–

Numbers of DMR genes shared across the 6 enriched GO term clusters (above and right of diagonal) with uncorrected Fisher’s exact test p-values for each test (below and left of diagonal). The highly significant overlaps within Dev1–2 or Act1–4 term clusters were considered somewhat trivial, but bold text indicates highly significant comparisons across Dev and Act clusters. Also see Fig. 6a, which shows that some of the overlap across Dev and Act clusters can be attributed to DMR genes in the Axon guidance, ErbB signaling, and Cell adhesion molecule KEGG pathways

### DMR gene markers of hypothalamic GABAergic neurons

To investigate how specific the DMRs were to hypothalamic cell types we compared DMR genes to groups of hypothalamus cell type markers that came from unbiased clustering of mammalian single-cell RNA-seq expression data [57]. These genes were not necessarily overexpressed, but composed co-expression groups corresponding to 15 types of glutamatergic neurons, 18 types of GABAergic neurons, and six stages of oligodendrocyte development.

**Fig. 7:**
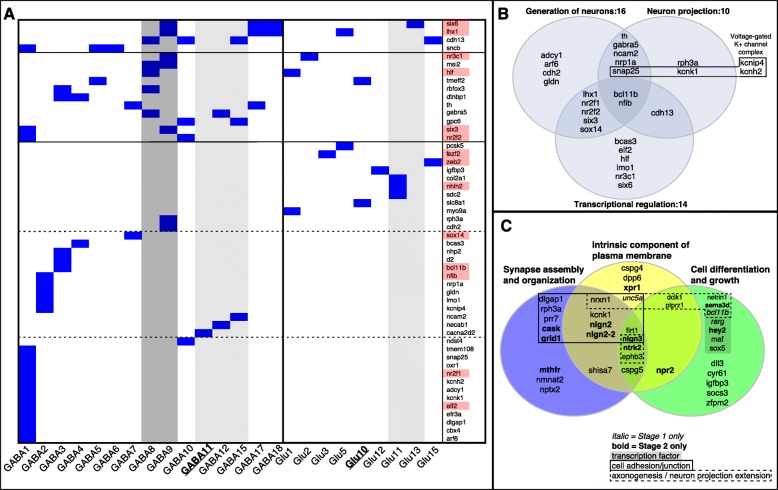
DMR genes in specific hypothalamus cell-types. **a:** Distribution of DMR genes (rows) across GABAergic and glutamatergic neuron subtypes in the hypothalamus (columns) from [57]. Blue cells indicate presence of a gene in a cell type, and pink shading behind gene names indicates transcription factor genes. Starting from the bottom, rows represent DMR genes that were found in progressively more cell types. Most genes were in one type, 11 genes were in 2 types, and 4 genes were in more than 2 types, as demarcated by the solid black horizontal lines. Dashed horizontal lines are for visual reference. Starting from the left, the first 15 columns represent GABAergic clusters sorted by the number of DMR genes they contained, then the final 9 columns (right of solid black vertical line) represent glutamatergic clusters. Note that not all clusters from [57] contained a DMR gene. GABA8 and GABA9 represent cells in the suprachiasmatic nucleus (SCN, dark grey shading), while GABA11, GABA12, GABA15, Glu11, and Glu13 represent arcuate nucleus cells (ARH, light grey shading), and GABA11 and Glu10 represent GnRH neurons (bold and underlined). Blue, yellow, and green bars below columns match the colors in the venn diagram in **c**.**b:** DMR genes in GABAergic cell clusters across GO categories that were enriched specifically in these genes (Benjamini-Hochberg-adjusted *p* < 0.1): Generation of neurons (GO:0048699), Neuron projection (GO:0043005), Transcriptional regulation (GO:0006355). Black box groups genes that are part of voltage-gated potassium channel complexes. **c:** Venn diagram of some DMR genes that were in the stage 1 and/or stage 2 oligodendrocyte progenitor cell (OPC) coexpression clusters from [57]. See Additional file 2: Table S6 for all DMR genes in these clusters. Genes are shown from GO categories that were enriched specifically in OPC cluster DMR genes (Additional file 2: Table S8). **Bold** and *italicized* text indicate genes specific to the stage 2 or stage 1 clusters, respectively. The solid black box surrounds genes involved in cell adhesion, the dashed box surrounds genes involved in axonogenesis and/or neuron projection extension, and the shaded box surrounds transcription factor genes

There were 43 DMR genes in GABAergic neuron clusters (OR > 1.4, *p* < 0.04) and 15 in glutamatergic clusters (OR > 1.4, *p* < 0.16; Additional file 2: Table S6). Six were in both types of neurons (*cdh13, hlf, lhx1, nr3c1, six6, tmeff2*) and 14 were transcription factors (OR > 3.5, *p* < 5e-4; Fig. 7a). Altogether, they made up 16/63 neuron overexpression markers among the DMR genes (OR > 5.5, *p* < 3e-6; Additional file 2: Table S6), including tyrosine hydroxylase (*th*), beta-synuclein (*sncb*), *rbfox3(102,308,260*; aka NeuN*)*, and *cacna2d2* (see Discussion). Notably, *rbfox3* had four DMRs, three of which had CNEs, including one with a putative binding site for *nr3c1*. DMR genes in GABAergic cell clusters were enriched for GO terms related to neural development (BH-adjusted *p* < 0.1, Fig. 7b).

The strongest enrichments for DMR genes in specific neuron types were in two GABAergic clusters representing vasoactive intestinal peptide (VIP, GABA9) and arginine vasopressin (AVP, GABA8) cells in the mammalian suprachiasmatic nucleus (SCN: OR > 2.7, uncorrected *p* < 0.004; Fig. 7a). Also of note were 15 DMR genes in a cluster representing somatostatin neurons (GABA1), nine in glutamatergic neurosecretory cell clusters (Glu10–15), ten in arcuate nucleus cell types (Glu11,13, GABA11–12, GABA15), and 23 in cell types with genes differentially expressed after food deprivation (Glu5,8,12, GABA1,11,15,18).

### DMR gene markers of early oligodendrocyte development

Many DMR genes were in clusters representing stages 1 (*n* = 42) and 2 (*n* = 57) of hypothalamic oligodendrocyte development (both OR > 1.7, BH-adjusted *p* < 0.013; Additional file 2: Table S6), including 15/22 oligodendrocyte progenitor cell (OPC) overexpression markers in the DMR genes (OR = 24.1, *p* < 3e-11; Additional file 2: Table S6). Thirty-four genes were in both the stage1 and 2 clusters. Notably, retinoic acid receptor *rarg (102310532)* was specific to stage 1, while neuroligins *nlgn2* and *nlgn3*, BDNF/NT-3 growth factor receptor *ntrk2(102296022)*, and transcription factor *hey2* (Fig. 1c) were specific to stage 2. DMR genes in these clusters were enriched for 15 GO terms (BH-adjusted *p* < 0.1; Fig. 7c, Additional file 2: Table S8).

## Discussion

### Neural plasticity, DNA methylation, and *A. burtoni* social change

*A. burtoni* social change involves neural plasticity at the synaptic, cellular, and circuit levels (reviewed in [58]). Synaptic/Hebbian plasticity has not been directly studied in *A. burtoni*, but it likely underlies observations of experience guiding male social behavior [50–53], and is probably important for learning optimal behavioral strategies. Synaptic plasticity has been linked to phenomena that are pertinent to *A. burtoni* social dynamics in other species, including fear conditioning [59], inhibitory avoidance [60], and spatial learning [61]. Also, cell-wide/non-Hebbian changes affecting neural circuit function in the hypothalamus occur after *A. burtoni s*ocial ascent; GnRH1 neurons increase in size and dendritic complexity [35, 36, 45, 46], altering their firing and connectivity [47, 48], somatostatin neurons grow [54], and adult neurogenesis increases [44]. Since all of these changes can revert if the social environment is altered *A. burtoni* exemplifies “metaplasticity”, i.e. plasticity of plasticity.

### DMRs genes involved in synaptic plasticity

**Fig. 8:**
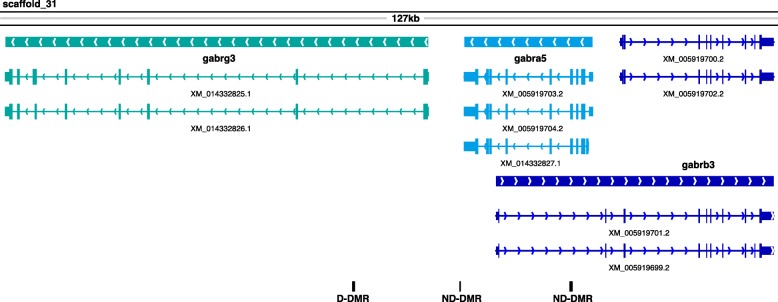
Genomic region with three GABA receptor genes and DMRs. Schematic of a 127 kb region on scaffold 31 of the *A. burtoni* genome that contains multiple GABA receptor genes and DMRs. Solid rectangles above gene names indicate their complete spans and arrows show which strand the genes are on. Individual isoforms are named with their NCBI-assigned transcript ids and are colored to match their respective genes. The positions of one D-DMR and two ND-DMRs are indicated with black rectangles below the genes

Many of the 716 genes we found with a DMR in their body or regulatory region are known players in synaptic plasticity in other species. Most encode molecules that make up the core machinery of glutamatergic and GABAergic synapses, including multiple types of glutamate and GABA receptors, transient and voltage-activated ion channels, intracellular scaffolding molecules, other key intracellular effectors, and trans-synaptic cell adhesion molecules like neuroligins and neurexins. Overall, 8/57 glutamate receptor genes and 6/33 GABA receptor genes in the *A. burtoni* genome had DMRs, which was highly significant (both OR > 5.8, *p* < 3e-4). Some had > 1 DMR in their body and/or regulatory region, and a genomic region with three GABA receptor genes and three DMRs was a compelling example of where methylation may affect splicing to fine-tune the ratios of receptor subunit isoforms (Fig. 8).

Beyond glutamate and GABA signaling, many DMR genes modulate synaptic function, for example through retrograde messaging and steroid hormone activity. DMR genes were enriched for retrograde endocannabinoid signaling (see Additional file 2: Table S5B), which affects neurotransmitter release via interactions with neuroligins and neurexins [62, 63]. Also, nitric oxide (NO) synthase adaptor protein *nos1ap(102312463)* had a DMR, and knockout of *nos1ap* in mice leads to altered dendritic spine morphology [64], thus it is plausible that *nos1ap* is involved in the GnRH1 cell dendritic changes after social ascent, or in other neuroendocrine cells (more on this below).

Other DMR genes could also be involved in dendritic remodeling after *A .burtoni* social change, and make up some of the common mechanisms underlying synaptic and homeostatic plasticity. For example, *arhgap32* (aka RICS), β-catenin interacting protein *ctnnbip1*, and N-cadherin *cdh2(102297691)* all had DMRs, and interactions among RICS, β-catenin, and N-cadherins affect dendritic structure in mammals [65]. In total, seven cadherin genes had DMRs, and cadherin-catenin complexes are implicated in many processes underlying synaptic and homeostatic plasticity, including CREB (cAMP response element-binding protein)-mediated transcription [66–69].

### DMR genes involved in homeostatic plasticity

Our results suggest that DNA methylation is involved in regulating homeostatic plasticity related to *A. burtoni* social status through mechanisms like CREB-mediated signaling, steroid hormone activity, and protein degradation. Several genes directly related to CREB/cAMP had DMRs (*adcy1*, *atf6b, crebrf, crtc3, ubxn4*). *crtc3* regulates steroidogenic acute regulatory protein (*StAR*), a CREB-activated gene linked to increased steroid hormone levels during social behavior in *A. burtoni* [70]. *atf6b* and *crebrf* are regulators of protein degradation via the unfolded protein response (UPR), which activates endoplasmic-reticulum (ER)-associated protein degradation (ERAD) in response to altered calcium homeostasis in the ER [71]. ERAD is thought to modulate homeostatic plasticity via activity-dependent degradation of GABA receptors [72, 73]. The most significant DMR overlapped the TSS for *ubxn4,* a critical molecule for ERAD [74, 75], and there were several DMRs in genes for GABA receptors.

Ubiquination and degradation of AMPA and GABA receptors may be mediated by DMR genes, thereby helping to maintain cell homeostasis, and ERAD function is modulated by ubiquitination [76, 77], Overall, we identified DMRs in several E3 ubiquitin ligases, ubiquitin-specific peptidases (e.g. *usp32*), and small ubiquitin-related modifier *sumo2(102294941)*. AMPA receptor and and PSD-95 ubiquitination are important for receptor endocytosis, degradation, and surface expression [78, 79]. Here, the AMPA receptor gene *gria4(102292924)* had a DMR, and a significant number of DMR genes interact with PSD-95 (encoded by *dlg4*; see Additional file 2: Table S7). Also, glutamate receptor-interacting protein (GRIP1) is essential for AMPA trafficking during synaptic scaling [80], and it interacts with DMR genes like hormone receptors (*nr3c1*, *thra (102296139)*), and *cspg4* (aka neural/glial antigen 2, *ng2*; Additional file 2: Table S7).

The androgen receptor (AR) is a steroid hormone receptor that affects specific *A. burtoni* social behaviors [81, 82], and our results suggest it may act in this context by modulating intracellular calcium and ERAD. There are two *A. burtoni* AR subtypes [83], and their expression in the brain correlates with that of GnRH1 [42]. Neither was differentially methylated in our study, but several DMR genes mediate AR or its effects in various contexts in other species: *hey2* (Fig. 1c), *pkn1(102295574)* and *ncoa2*, *usp12, usp32* (Fig. 1b)*,* and *zmiz1(102310819)* [84–88]. Of particular interest here, AR regulates aspects of the UPR in non-neural tissue [89, 90]. It is an open question whether AR plays a similar role in the brain of *A. burtoni*. But, a DMR in the gene for voltage-gated calcium channel (VGCC) subunit *cacna2d2*, contained a CNE with a putative AR binding site, suggesting that this is likely a true genomic target of AR.

The involvement of DMR genes in molecules and mechanisms that play roles in homeostatic plasticity is particularly intriguing with respect to GnRH1 and somatostatin cells, whose growth after *A. burtoni* social change must necessitate considerable homeostatic regulation, and some aspects of this section are revisited later in the Discussion on GnRH1 and other neuroendocrine cells below.

### DMR genes involved in adult neurogenesis

Adult neurogenesis is ongoing in *A. burtoni* and amplified in D males [44], thus DMRs in developmentally important genes may play a role in this difference. For example, the strong enrichment of genes in the axon guidance pathway could reflect the integration of new neurons into existing circuits. Notably, development-related DMR genes were not fundamentally distinct from those important for synapse structure and plasticity. Almost half of the DMR genes in enriched GO terms were involved in development and activity, including all in the ErbB signaling pathway, > 90% in the axon guidance and cell adhesion pathways, and > 75% in the insulin secretion, oxytocin signaling, Rap1 signaling, and circadian entrainment pathways. Also, 5/6 GO term clusters were enriched for genes in the OPC clusters from [57], 19 of which were involved in development and activity, including transcription factors (*bcl11b, hey2, nr2f2(102292088), rarg (102310532)*), axon guidance (*ntn1, sema3d, unc5a*), and cell adhesion molecules (*cdh13, nlgn2, nlgn3, nrxn1*).

DMRs in OPC genes may modulate integration of adult-born glia and/or neurons into existing circuits, including around hypothalamic neuroendocrine cells. We found no evidence of OPC-GnRH1 interactions in the literature but GnRH1 cells are in close contact with other glia that play a critical role in GnRH1 secretion [91]. For example, GnRH1 neuronal activity is reduced in mice with impaired prostaglandin E2 signaling that is due to defective ErbB signaling in astrocytes [92]. DMR gene *hpgd* regulates prostaglandin E2 [93] and *erbb4(102292440)* was one of the more prominent DMR genes involved in many functions. The DMR gene *cspg4* (aka *ng2*, see above), was in both OPC clusters, and *cspg4* knockout in mice affects glutamate signaling and behavior [94]. *cspg4*-positive OPCs are unique among glia in forming glutamatergic and GABAergic synapses with neurons and functionally integrating into neural networks [94].

### Potential roles for differential methylation in regulating GnRH1 cell function

The electrical properties of GnRH1 neurons are extremely complex and there are many DMR genes with the ability to play subtle roles in their modulation. Mammalian studies have shown that GnRH1 neurons generate activity-dependent, long duration calcium transients that arise via interplay between electrical activity, VGCCs, intracellular calcium release from the ER, GABA and AMPA receptors, and calcium-activated potassium (SK) channels [95]. In addition to the multiple GABA receptors and AMPA receptor *gria4* that had DMRs, other DMR genes can affect calcium dynamics by modulating one of the above mechanisms, potentially in relation to AR or *erbb4*: VGCC subunits (*cacna2d2, cacna1b(102311676)*), ryanodine and BDNF receptors (*ryr2, ntrk2*), and Q subfamily voltage-dependent potassium channels (*kcnq1(102292488), kcnq3*).

Three DMR genes were in coexpression clusters corresponding to GnRH cells in [57] (*cacna2d2*, *tmeff2, slc8a1*; see Fig. 7a). *cacna2d2* acts as a regulatory subunit in L-type VGCCs, and its DMR contained an AR binding site just upstream of an anti-sense lncRNA. L-VGCCs activate *ryr2* to release calcium from intracellular stores in muscle cells and a similar mechanism could exist to release calcium from the ER in GnRH1 cells. *slc8a1* (aka *ncx1*) is located on the plasma and ER membranes, and regulates intracellular calcium. There is evidence that its effects prolong calcium transients in dendritic spines and contribute to synaptic metaplasticity [96]. Finally, *tmeff2* is a transmembrane protein that inhibits cell growth, is regulated by androgen in cancer cells [97], and is a potential ligand for *erbb4* [98].

Changes in potassium conductance and Q subfamily potassium channels mediated by DMR genes could contribute to the difference in GnRH1 repolarization across *A. burtoni* social status [47], and play a role in stabilizing GnRH1 calcium transients after changes in cell membrane properties. First, multiple voltage-gated potassium, calcium, and chloride channel genes had DMRs (Additional file 2: Table S1B). Also, *A. burtoni* GnRH1 neurons are coupled by gap junctions [48] and inhibited via dopamine D2 receptors that are likely coupled with potassium channels [99]. Multiple DMR genes could influence interactions between chemical and electrical synapses, such as NMDA receptors (*grin2a, grin3b*) or genes involved in endocannabinoid activity and cAMP signaling (*adcy1*) [100]. Finally, in mice, Q-type potassium channels like those encoded by DMR genes *kcnq1* and *kcnq3* are inhibited by a chemical that blocks the GnRH1 sAHP current, but only when the membrane potential is positive; *kcnq3* is enhanced when the membrane potential it is negative [101].

This last point is intriguing with respect to the similarity between social ascent and puberty [37] and the excitation of GnRH1 cells by GABA, which has not been tested in *A. burtoni* but does exist in other teleost fish [102, 103]. Work in mammals has linked GABA excitation to development and shown that the switch from depolarization to hyperpolarization occurs during puberty [104, 105]. Expression patterns of GABA receptor subunits in GnRH1 cells change during development and involve activation of these neurons at puberty in mammals [104], and similar processes could be at work in *A. burtoni* GnRH1 cells every time social change occurs. In addition, there is evidence that retrograde endocannabinoid signaling regulates excitatory GABAergic inputs to GnRH1 cells [106], and several DMR genes are involved in this pathway.

### Potential roles for differential methylation to influence other neuroendocrine cells

The samples we analyzed contained many types of neurons and glial cells from the hypothalamus and some adjacent areas (see Methods). Since methylation patterns are cell-type specific in the brain [107], we explored whether DMR genes could be indicative of processes in particular cells, finding that they were enriched in coexpression clusters from [57] representing hypothalamic AVP and VIP cells in the mammalian SCN. We also performed multiple layers of DMR curation, partly to remove DMRs that could have reflected an imbalance of extra-hypothalamic tissue across the samples (see Methods). We caution that while cell types are generally conserved across vertebrates, homologous brain structures such as the SCN and arcuate nucleus are not as straightforward to identify. Nevertheless, our results were intriguing. The SCN is known for its role in circadian timing and DMR genes were enriched in the circadian entrainment KEGG pathway, but interestingly none were among the DMR genes in the SCN clusters. Instead, almost all were part of multiple pathways and/or synapse types, representing a core set of DMR gene signaling molecules (Additional file 2: Table S5B-C). The only two DMR genes specific to the circadian entrainment pathway were aforementioned *nos1ap*, and the T-type voltage-gated calcium channel *cacna1i(102289833)*, which encodes a calcium channel subunit involved in the pacing of neuronal firing and network oscillations. It may be affected by DMR gene *gnao1(102305147)*, which modulates presynaptic calcium channels, and could directly affect the activity of calcium-sensitive DMR genes in GABAergic cells like *adcy1, cask, kcnip4, necab1,* and specifically *cdh13* and *rph3a* in the SCN.

A recent study deleted *erbb4* - a prominent DMR gene - specifically in mice VIP neurons, causing long-term dysregulation of cortical function that emerged during adolescence [108]. This is intriguing due to the similarities between *A. burtoni* social ascent and puberty [37] and the functional connections between VIP and GnRH1 neurons [109]. If *erbb4*’s effects on neural function are connected with onset of puberty in other species, it could play an analogous role during *A. burtoni* social ascent. *erbb4* deletion in mice interrupted the ability of cortical circuits to adapt to ongoing cognitive and behavioral demands, and to accurately encode information about behaviorally relevant environmental features, abilities absolutely central to successful *A. burtoni* social ascent. It also eliminated neural network synchrony, disrupting temporally organized activity that is key for information processing [110]. Interestingly, *erbb4* is a receptor for neuregulins, and two copies of the neuregulin 3 gene (*102,306,195, 102,314,635*) had DMRs; in one DMR methylation was higher in D fish, while in the other it was higher in ND fish, suggesting different patterns of regulation with respect to social status, and potentially evolutionary subfunctionalization.

### Implications for female *A. burtoni* social status

Most work on *A. burtoni* social behaviors and status has focused on males, but females can also form social hierarchies. Similar to males, aggressive behaviors and androgen levels increase in D females [111], and social defeat hampers sensory information processing [112]. In contrast, relationships between hormones and dominance behaviors differ from those in males [111], and proactive social behavior seems to protect against major sensorimotor deficiency in ND females [112]. D females also do not show the dramatic increases in growth rate and reproductive ability characteristic to D males [113], although volatility in female social status may be tied to their reproductive cycle [111].

It is reasonable to think some of the differences we identified in DNA methylation could also be found in females. For example, DMRs in *nr3c1* and genes related to oxytocin signaling or ErbB signaling (see Additional file 2: Table S5B) in VIP neurons may exist in D females. *nr3c1* and *oxtr* methylation has been linked with aggressive behavior in mammals [114], and VIP-specific *erbb4* knockout in mice disrupts neural activity key for information processing in the context of reproductive maturation (see above). Additionally, disrupting *nrg3* or VIP signaling in mice leads to sensorimotor deficits similar to those in ND male and female *A. burtoni*, although the hypothalamus was not specifically implicated in these studies [115, 116].

On the other hand, while male reproductive capability increases as a result of social ascent, in females the ability to maintain D status seems contingent on their reproductive cycle [111]. Thus, for social status DMRs in genes that we linked to puberty-related phenomena in mammals - ErbB signaling and GABA excitation in neuroendocrine cells - it is not clear whether they would be found in a methylation study of female social status. The expectations for DMRs in genes affecting growth and/or GnRH1 activity are also murky, given that female social status is not correlated with growth rate or gonad size, two major features of *A. burtoni* D males, but androgens are elevated in D females.

### Transcriptional plasticity, DNA methylation, and *A. burtoni* social change

Experience-dependent changes in methylation can direct later transcription and plasticity through regulation of alternative splicing and TEs, or by priming genes for future transcription [117–119], and our results highlight many ways that methylation could differentially modulate transcription in D versus ND *A. burtoni*. These included directly increasing/decreasing expression by methylation near the 5′ or 3′ ends of a gene, regulation of transcription factor expression, differentially regulating gene duplicates, and interactions with other epigenetic mechanisms. DMR genes had high numbers of TEs, splice variants, and antisense lncRNAs compared to other genes, suggesting that the physiological consequences of *A. burtoni* social change are mediated by interplay between multiple epigenetic systems in potentially subtle ways that would not be detectable in overall measures of gene expression.

Nuclear hormone receptors are well-studied in the context of *A. burtoni*. Social status, and our results link their differential methylation with another prominent epigenetic mechanism, histone deacetylase (HDAC) activity. Eight DMR genes were nuclear receptors or coactivators, for example *nr3c1* and *thra*, and 18 interact with several nuclear receptor coregulators. These included the transcription factor *hey2*, which had one of the most significant DMRs (see Fig. 1), and two HDACs: *hdac4(102312396)* and *hdac7.* In total, 18 DMR genes interact with HDACs, including 6/18 nuclear receptor related genes, and two copies of the gene for histone H3.3 (*h3f3a: 102308796, 102,309,100*), which is associated with gene activation and specific methylation marks.

Surprisingly few DMRs overlapped a TE, but the location of TEs in and around genes was predictive of having a DMR. Also, the number of TEs in DMR gene bodies was related to how much methylation changed and gene function. There were more TEs in DMR genes related to neural activity as opposed to development, particularly those that interact with MAGUK family proteins like PSD-95 in glutamatergic synapses. TE movement could influence and/or reflect *A. burtoni* social change since in other species it occurs in adults, is elevated in the brain [118, 120, 121], occurs in response to environmental stimuli [122, 123], and may generate unique experience-dependent transcriptomes of individual neurons [124].

Experience-dependent alternative splicing affects synaptic plasticity [125], and regulation of alternative isoforms may be reflected in altered ratios of splice variants but not overall gene expression changes [126], which could be related to the lack of correlation between methylation and expression we found in genes with a DMR in their body. An intriguing example of DMRs potentially affecting splicing was in *rbfox3* (aka NeuN), a massive gene (431 kb) with 10 splice variants and four DMRs, three of which were in introns upstream of the TSSs for different variants. Thus, methylation could affect relative expression levels of *rbfox3* splice variants, meaning these DMRs had the potential to affect the splicing of a gene that is itself a splicing regulator. One *rbfox3* DMR contained three CNEs and potential binding sites for *nr3c1*. It was near the gene’s 3′ end and may also affect the expression of downstream *grin2c*, an NMDA receptor gene.

*rbfox3* was in the AVP coexpression cluster from [57] with other DMR genes that demonstrate the multi-layered regulatory potential of the DMRs (*cdh13, gabra5, hlf, msi2*). *msi2* is a post-transcriptional regulator that inhibits translation (*msi1* also had a DMR). *cdh13* had a DMR near each end of the gene, and only the 5′ DMR contained a CNE and potential estrogen receptor binding sites. *gabra5* was in a region with two other GABA receptor genes and three DMRs, all of which had the potential to regulate expression or splicing of one or more of the genes (Fig. 8). Finally, the DMR 2.5 kb upstream of *hlf* was also 1 kb upstream of a lncRNA.

The functions of some DMR genes involved in synapse plasticity are highly dependent on alternative splicing in other species. For example, splicing of neuroligin and neurexin genes affects function in ways specific to glutamatergic versus GABAergic synapses [127, 128] and different aspects of synapse formation and function [129]. Also, context-specific alternative splicing of *arhgap32*/RICS is crucial for its function. One isoform is expressed primarily in postsynaptic membranes and neurite growth cones and another is in the ER and Golgi, where it is involved in transport of molecules like N-cadherin from the ER to Golgi [130], and *ergic2(102314043)*, a chaperone molecule involved in ER-Golgi transport also had a DMR.

Finally, many DMR genes had an antisense lncRNA (Additional file 1: Figure S10A; Additional file 2: Table S4), including nuclear hormone receptors (*nr2f2, nr6a1, rarg*) and genes involved in calcium dynamics (*cacna2d2, ryr2, slc8a3*), signal transduction (for example *ctnnbip1, erbb4*), and axon guidance (*sema6b, nrxn3b, plxnb1*). Antisense lncRNAs can interfere with the splicing, RNA editing, sub cellular distribution, transport, or nuclear retention of the corresponding sense RNA, and can alter mRNA stability and modulate translation [131, 132].

Two particularly intriguing lncRNA-overlapping DMR genes were retinoic acid receptor gamma (*rarg*) and VGCC *cacna2d2*. The *rarg* DMR overlapped an entire coding exon as well as the TSS for an antisense lncRNA. It was just downstream of the TSS for a truncated *rarg* splice variant and contained potential retinoic acid and estrogen receptor binding sites. Retinoic acid is known to mediate synaptic scaling [133] and the retinoic acid induced transcription factor *rai1* also had a DMR. The third intron of *cacna2d2* contains an antisense lncRNA. A DMR was in the same intron ~ 10 kb upstream of the lncRNA and contained a CNE with a putative AR binding site, suggesting that this is likely a true genomic target of AR and regulated differentially as a function of *A. burtoni* social status.

### Stress and glucocorticoid receptor methylation

Differential methylation of the glucocorticoid receptor gene *nr3c1* can affect multiple mechanisms of neural and transcriptional plasticity. In male *A. burtoni*, as in many species, levels of stress and reproductive hormones reflect the social standing of individuals. ND males have low androgen and GnRH1 levels, but high levels of stress-related corticotrophin releasing factor (CRF) and cortisol [134], which binds to GR. CRF suppresses reproductive function, potentially by acting on GnRH1 cells, which express GR. Many fish species including *A. burtoni* have two glucocorticoid receptor genes, one of which had a DMR here (*nr3c1*, referred to as GR1 in [135]). One isoform of this gene leads to less efficient cortisol binding and is expressed at higher levels in ND males, protecting GnRH1 cells and allowing reproductive neural circuits to remain relatively intact during chronic stress [135]. It is possible that these relative isoform expression levels are shaped by the DMR that we identified in the body of *nr3c1*.

DMRs could also affect the ability of GR to regulate other molecules. GR can downregulate brain-derived neurotrophic factor (BDNF) expression through direct binding [136], and the DMR in BDNF receptor gene *ntrk2* contained a putative GR binding site. GR/BDNF methylation has been extensively studied with respect to early life experience, chronic stress, neuropsychiatric disorders, and synaptic plasticity in mammals [137–140], and other DMRs in important plasticity-related genes contained putative GR binding sites as well, such as *nrxn1*, *nlgn2(102313675)*, *nlgn3*, and *ntrk2*. GR can also rapidly and selectively stimulate endocannabinoids or NO to suppress excitatory or facilitate inhibitory synaptic inputs, respectively, in neuroendocrine cells [141], and the inhibitory effect of stress hormones on neurogenesis seems to result from disruption of the excitation-inhibition balance in neural progenitor cells caused by aberrant GR activation [142].

A classic example of methylation and transcriptional priming involves *nr3c1* [143], and there are many scenarios wherein methylation of specific binding elements or transcription factors could dynamically orchestrate transcriptional priming or bookmarking genes in just D or ND males, leading to contextually-appropriate behavioral responses. It is possible that in ND males, for example, methylation of *nr3c1* primes transcription of genes important for aggressive territorial behaviors only when D males are absent, but the lack of this epigenetic mark in D males would allow aggression-related genes to be expressed constitutively.

### Considerations related to small sample size

Due to resource limitations, only two fish of each social status were assayed in this study. This small sample size is obviously not ideal, and readers should take appropriate caution when interpreting the results. However, we were careful and conservative in our approach, and have confidence that the results reliably fulfill the main aims of this study: 1) creating a genome-wide picture of DNA methylation in the brains of socially dominant and non-dominant *A. burtoni*, and 2) building a resource to facilitate the generation of novel hypotheses.

We tailored our methods in several ways to limit sample size-related worries as much as possible. First, BSmooth [55] was used to detect DMRs because it can produce robust findings with small sample sizes. An extensive analysis of multiple whole-genome bisulfite sequencing datasets found that improvements in true positive rates (TPR) and false discovery rates (FDR) for BSmooth DMR detection were minimal when the number of samples per group increased from two to three, and that TPR gains fell off rapidly beyond 8-10X genomic coverage (see [144] - Fig. 2; mean coverage here was ~8X, see Methods section “BS-seq read alignment and calculation of CpG methylation ratios”). Second, because there were no gold-standard DMRs or a high-quality publicly available linkage map for *A. burtoni*, we ran BSmooth multiple times and kept only the DMRs that were robust to varying the main parameters (see Methods section “Smoothing and identification of differentially methylated regions (DMRs)”). Third, because some DMRs could have reflected individual differences in, e.g., life history or dissection variability, and not necessarily social status, we permuted the social status labels and repeated the DMR detection process, then filtered out any of the actual DMRs that were also identified from the permuted data (see Methods section “Filtering putative DMRs for robustness and social status specificity”). Finally, to determine whether the distribution of DMRs in/near genes and other functional genomic features was biologically meaningful, we bootstrapped the final list of filtered DMRs to create pseudo-random lists of “null DMRs”, which allowed us to estimate the likelihood of observing the genomic distribution of the actual DMRs by chance (see Methods section “Relating DMRs to genomic features and nullDMR generation”).

## Conclusions

Our results revealed genome-wide effects of *A. burtoni* social status on DNA methylation in the hypothalamus and strongly suggest a role for methylation in plasticity across multiple biological levels. DMRs had the potential to affect transcription directly or through interactions with other epigenetic mechanisms, many DMR genes are known to be important for multiple forms of synaptic and homeostatic neural plasticity, and genes key to status-specific behaviors could be primed by methylation for transcription only when specific environmental and/or internal conditions are met.

GnRH1 neurons ultimately control reproduction and our results suggest multiple ways their unique electrical properties and calcium dynamics could be influenced by social status-specific methylation patterns. Mechanisms of homeostatic plasticity are likely important in GnRH1 cells (and others like somatostatin cells) that grow dramatically after social ascent, but they also receive highly complex inputs via different neurotransmitters and neuromodulators, and synaptic plasticity is probably crucial to optimal GnRH1 function as well. GnRH1 and other neuroendocrine cells can be excited by GABA in a manner potentially related to the onset of puberty, and at least one prominent DMR gene (*erbb4*) affects the function of mammalian GABAergic VIP interneurons and related neural circuits in a way that is only observable during adolescence. This is intriguing given that the physiological changes that accompany *A. burtoni* social change strongly resemble a form of repeated puberty that is contingent on the social environment and behavior.

We chose to focus on genes related to neural plasticity and related signaling pathways but there were functional themes and molecular interactions related to, for example, immune function, chromatin modification, and growth factors (insulin-like, transforming, fibroblast, vascular endothelial, growth arrest-specific, and brain-derived neurotrophic) that were well-represented in the DMR genes. There were DMRs in some well-studied genes like *gsk3b*, β-synuclein (*sncb*), and various disease-related genes that did not easily fit into the discussion (see Additional file 2: Table S9), so we encourage readers to explore the Additional file figures and tables. Our results also implied that subfunctionalization of duplicated genes could be affected by methylation. In some cases more than one copy of a gene had a DMR, often in different parts of the gene, and we expect that a deeper analysis of the relationship between methylation and gene duplication would yield exciting novel information relevant to the evolution of social behaviors.

Going forward, our findings and data can be used as a resource to generate new hypotheses about *A. burtoni* social status (e.g. see Table 4), and more generally, the relationships between behavioral, neural, and transcriptional plasticity in the context of social status.

**Table 4:** Example hypotheses inspired by DMR findings

Finding(s)	Hypothesis
Top DMRs in ERAD genes, e.g. *ubxn4*, possible connections to AR	ERAD modulation by AR necessary to stabilize neural circuits that include GnRH1 and somatostatin cells after social ascent.
*cacna2d2* DMR upstream of lncRNA has AR binding site & CNE	lncRNA regulates *cacna2d2* under specific control of AR in the context of altered Ca2+ currents in cells that recently changed size
DMR in *erbb4* and interactors, DMR genes in VIP gene clusters	*erbb4* signaling in VIP cells modulates neural circuit changes related to reproductive capability, D fish lacking *erbb4* may struggle to mate or maintain status
DMRs in *nr3c1*, *nos1ap*, GABA/glutamate/endocannabinoid pathways	GR stimulation of endocannabinoids/NO selectively regulates excitatory/inhibitory signals onto VIP and AVT cells, based on social status
Intronic DMR in *nr3c1*	DNA methylation governs the expression of specific *nr3c1* isoforms, driving status-specific cortisol sensitivity

This table summarizes some results from this study in column 1 and hypotheses they inspired in column 2. This is by no means an exhaustive list, but we think these represent some provocative but testable ideas that would not have arisen if not for this whole-genome scan of methylation patterns in the context of *A. burtoni* social status

## Methods

### Animals and tissue collection

*A. burtoni* derived from wild-caught stock [34] were maintained in aquaria under conditions mimicking their natural habitat (28 °C, pH 8, 12 h:12 h full-spectrum light:dark cycle, constant aeration and water chemistry matched to that of Lake Tanganyika), and fed cichlid flakes (AquaDine) and brine shrimp once a day. Fish were reared in community tanks (∼35 fish per 114 L tank, 91.4 × 55.9 × 30.5 cm, l × w × h) with four terra cotta pots cut in half lengthwise to produce a truncated half cone territorial sites (11 × 11 × 5.5 cm, l × w × h).

Pairs of size- and age-matched males (within 10% body length and 1 week) were moved to smaller (30 L) tanks that contained three reproductively able females. We restricted our experiments to males that were 5–6 months of age and chose animals that showed few territorial behaviors in rearing tanks to minimize differences in life-experience across competitors. Within 1–2 days a stable dominant(D)-nondominant (ND) dyad relationship was established between the two males and they remained in this configuration for 3–6 weeks, since suppression of the reproductive axis requires this length of sustained social suppression [134].

To collect tissue for sequencing, two D and two ND males from four separate dyads were sacrificed via cervical transection in the morning and brains were immediately extracted and dissected. A coronal (transverse) cut was made in the caudal telencephalon, but rostral to the optic nerves/chiasm to include the nPPa. Then we made a diagonal-horizontal cut above the inferior lobe of the hypothalamus that was angled dorsally at the rostral side. Thus samples contained the hypothalamus, including the POA and GnRH1 cells, but potentially also some thalamic areas & caudal telencephalon areas rostrally. Tissue was flash-frozen and stored at -80C. Both D males had gonadosomatic indices > 0.8 (GSI; ratio of gonad weight to body weight × 100) and ND males had GSI < 0.3. All experimental procedures were approved by the Stanford Administrative Panel for Laboratory Animal Care.

### DNA/RNA isolation, library construction, and sequencing

DNA and RNA were purified from hypothalamic samples using a standard protocol for DNA and RNA extraction (Qiagen AllPrep DNA/RNA Mini kit). Nucleic acid yield (Qubit spectrophotometer) and quality (Agilent Bioanalyzer) were verified before sequencing library preparation. Strand-specific bisulfite-treated genomic DNA libraries for BS-seq and cDNA libraries for RNA-seq were prepared following established protocols [145, 146]. Library quality was verified (Agilent Bioanalyzer) and sequencing was performed (Illumina HiSeq 2000 platform), generating 101 bp paired-end read data. For BS-seq, each library was sequenced in its own lane, with an average of ~ 125 million read pairs passing quality filters for each sample (~ 96% of total reads). For RNA-seq, samples were processed to remove rRNA (Epicentre Ribo-Zero rRNA removal kit), then barcoded with standard Illumina index sequences and sequenced in a single lane, yielding an average of ~ 38 million good quality read pairs per sample (~ 95% of total reads).

### BS-seq read alignment and calculation of CpG methylation ratios

Fastq files of raw sequencing reads were aligned to the *A. burtoni* genome using BSMAP 2.9 [147]. Default values were used for all BSMAP parameters except the following: -A (3′ adapter sequences to trim: GAGCCGTAAGGACGACTTGG and ACACTCTTTCCCTACACGAC; default = none), −q (trim bases below quality score: 30; default = 0), −m (minimum allowed insert size: 0; default = 28), and -S (seed for random number generator to select from reads with multiple hits: 1, allows reproducible mapping results; default = read index number). The resulting SAM files were sorted, compressed into BAM files, and the bias-plot.py script [148] was used to plot mean methylation percentage as a function of base position within reads. This was stable around 85–86% for all four subjects except for positions 1–3 and 99–101 (Additional file 1: Figure S15), therefore the first and last three bases were trimmed from every read using fastx_trimmer [149], then reads were realigned using the same BSMAP settings. An average of ~ 56.9% of reads for each subject aligned to the genome in proper pairs. Since the *A. burtoni* genome is ~831mb long this yielded a rough average coverage of: (125 million reads) x (95 bp/read) x (.569 mapped) / (831 million bp) ~ 8X, exceeding coverage recommendations for the regional smoothing and DMR detection procedure used in downstream analysis [55, 144].

CpG methylation ratios were computed from BAM files using the methratio.py script included in the BSMAP release, with the following options set: -u (process only uniquely mapped read pairs), −p (process only reads mapped in proper pairs), −z (report loci with zero methylation ratios), −r (remove duplicated reads), −m (report loci with given minimum sequencing depth, set to 4), −g (combine CpGs across strands). Note that we use “methylation ratio” here to refer to the raw proportion of total reads at a given CpG locus that were not converted by the bisulfite treatment (thus indicating methylation). Throughout the paper we use “methylation level” to refer to the smoothed values of these ratios produced by BSmooth (see below). Both quantities ranged between 0 and 1, where 0 was no methylation and 1 was complete methylation. Part of the output from methratio.py is an estimation of cytosine coverage across the genome, which averaged ~21X across samples. Exact values of mapped reads and genome/cytosine coverage for individual samples are reported in Additional file 2: Table S10.

### RNA-seq alignment and assessment of gene expression

Cutadapt [150] was used to trim the first two bases from each read (−u 2, −U 2), low quality ends from both ends of each read (−q 30,30), and Illumina adapter sequences (−a AGATCGGAAGAGCACACGTCTGAACTCCAGTCAC, −A AGATCGGAAGAGCGTCGTGTAGGGAAAGAGTGTAGATCTCGGTGGTCGCCGTATCATT). Processed reads shorter than 50 bp were discarded (−m 50). Kallisto [151] was used to assign read pairs to annotated transcripts and quantify transcripts per million (TPM). All transcripts for a gene were summed to yield gene level expression values. Estimated read counts from Kallisto were used as input to functions in the DESeq2 R package [152] to generate log2(D/ND) differential expression fold-difference estimates.

### Smoothing and identification of differentially methylated regions (DMRs)

DMRs were identified using functions in the bsseq package for R [55, 153]. Briefly, we used BSmooth to generate smoothed methylation profiles for each subject, BSmooth.tstat to compute estimates of the mean differences and standard errors for each CpG across groups, and dmrFinder to find regions of consecutive CpGs that passed a significance threshold based on the marginal empirical distribution of the t-statistic, i.e. differentially methylated regions (DMRs).

BSmooth uses local-likelihood smoothing with the key parameters *ns* (minimum number of CpGs in smoothing window, default = 70), *h* (minimum number of total bases in smoothing window, default = 1000), and *maxGap* (maximum gap between two methylation loci before smoothing is broken across the gap, default = 10^8). These were optimized using the human genome, for example *maxGap* = 10^8 ensures that smoothing could not occur across chromosomes, and [55] recommend adapting them when applying BSmooth to non-human organisms. The public version of the *A. burtoni* genome [154] is spread across 8001 scaffolds, the vast majority of which are relatively small with roughly half that are shorter than 5 kb and only 3% that are 1mb or longer (median/mean length: 4.8 kb/104 kb). Each scaffold was smoothed separately, therefore *maxGap* did not affect our results except at values smaller than the length of the longest *A. burtoni* scaffold (~7mb), so we held it at 10^8 and ran BSmooth using all combinations of *n*= [25, 50, 70] and *h* = [1000, 750, 500]. Only DMRs identified in all nine of these smoothing runs were analyzed further (see below). Scaffolds with fewer than 70 CpGs with 4X coverage in all fish were excluded since they had undefined methylation estimates in the runs where *n* = 70, which left 2071 scaffolds containing 4.2 million loci. They represented 96% of the entire genome (median/mean length: 9 kb/384 kb) and 95% of all annotated genes [155].

We used the same BSmooth.tstat settings for processing the smoothed values from every combination of the BSmooth parameters: *estimate.var* = “same”, *local.correct* = TRUE, *qSd* = 0.75, *k* = 101. For dmrFinder, significance cutoffs were defined as the 0.025 and 0.975 centiles of t-statistics on each scaffold. Other settings were defaults: *maxGap* = 300 (maximum distance allowed between two CpGs in a single DMR), *stat* = “tstat.corrected”. Putative DMRs with less than three measured CpGs or an absolute mean methylation difference of 0.1 were removed, as per the recommendation of [55]. For example, the default BSmooth settings (*n* = 70, *h* = 1000) yielded 29,107 putative DMRs and this filter reduced that number to 5569, mostly because ~ 75% had too small of an absolute mean methylation difference. Ultimately, DMRs were identified on 335 scaffolds. Scaffold length was not significantly related to any of the following DMR characteristics: size, number of CpG loci or density, effect size, or whether methylation was higher in D or ND fish (Additional file 1: Figure S16). Custom R code for applying this analysis to a scaffolded genome, analyzing the results, and performing other downstream processing is available on the Fernald lab github repository [156].

### Filtering putative DMRs for robustness and social status specificity

We did not know how many DMRs to expect or what their statistical characteristics might be. BSmooth can identify true DMRs with minimal biological replicates, as shown in [55] using data from a fibroblast cell line [145], normal and tumor colon samples [157], and peripheral blood mononuclear cells [25]. But, these datasets were specifically selected for strong methylation signal with minimal variability and represent more homogenous tissue types than brain. Methylation patterns are cell-type specific, including in the brain [107], and our samples undoubtedly contained multiple types of neurons and glial cells. Also, fish life histories were controlled as much as possible (see above), but *A. burtoni* social behavior and physiology are interesting precisely because of their plasticity and it is difficult to fully control early life behavior while maintaining a naturalistic social environment. Thus we sought to minimize the chances of identifying putative social status DMRs that actually reflected extreme variability in the dissection or life history of one animal.

Toward this end we ran BSmooth multiple times and combined automated filtering with hand curation to identify DMRs that were both 1) robust to the smoothing procedure and 2) the most likely to be specific to social status. First, we ran BSmooth using all combinations of *n*= [25, 50, 70] and *h* = [1000, 750, 500]. DMRs were identified separately for each smoothing run as described above and compared, yielding 1872 “common” DMRs that were present in all nine runs; 900 exact matches and 972 with at least 100 bp overlap. Exact matches were found using the base R functions intersect and setdiff, and overlapping DMRs were found by converting them to GRanges objects and using the findOverlaps function in the IRanges package [158]. This process was repeated two more times after shuffling the social status labels to compare D1&ND1 vs D2&ND2 and D1&ND2 vs D2&ND1, with the key difference that every DMR from every run was retained, yielding 68,664 “shuffled” DMRs. Almost half of the common DMRs did not overlap a shuffled DMR at all (*n* = 854) but even more overlapped one by > 95% (*n* = 920). Any common DMR that overlapped a shuffled DMR by at least 50% was removed (*n* = 999), leaving 873 putative DMRs that were robust to smoothing parameters and unlikely to be driven by extreme values in one fish. Finally, the remaining common DMRs were curated to eliminate any that appeared to result from over-smoothing, were situations where one D and one ND fish were very different but the other two were similar, or appeared spurious for any other reason. Smoothed methylation levels and raw methylation ratios were visualized using the bsseq::plotManyRegions function and were examined by eye, and 164 suspicious DMRs were removed during this process, leaving 709 final DMRs.

### Annotating features in the *A. burtoni* genome

Gene annotations were acquired from NCBI [155]. These include gene models, mRNA transcript isoforms, exons, and lncRNAs. Almost half of the protein-coding genes in these annotations were assigned a gene symbol (for example *nr3c1*) but the rest were assigned an identifier beginning with “LOC” followed by nine numbers (as were lncRNAs). In the paper, the first time LOC genes are mentioned they are referred to first by the gene symbol of the best human or fish homolog (see functional enrichment section below), followed by the nine-digit LOC number in parentheses, for example *sema6b(102313929)*, then afterwards and in the figures they are only referred to by gene symbol for clarity.

Gene basal regulatory regions were defined as 5 kb up- to 1 kb downstream of the transcription start site (TSS, 5′) and 1 kb up- to 5 kb downstream of the end (3′) based on [159]. Transposable elements (TEs) were annotated by the BROAD Institute [154]. Conserved non-coding elements were determined using a set of 54,533 high quality conserved non-genic elements identified in zebrafish (CNEs) [160]. These sequences were aligned to the *A. burtoni* genome using hardware that was specifically built to optimize the Smith-Waterman algorithm for alignment of non-coding sequences [161]. 32,210 sequences matched with a bit score > 3500 and were retained as CNEs in *A. burtoni*.

Transcription factor binding site (TFBS) predictions were performed using position weight matrices from the JASPAR2016 database [162]. Nucleotide sequences for each *A. burtoni* genome scaffold were loaded into R using the getSeq function in the BSgenome package [163]. Both strands of each scaffold were scanned using the searchSeq function in the TFBSTools package [164] and hits with min.score > 90% were recorded as putative TFBSs. TFBS hits that overlapped a CNE were considered higher confidence. For comparison to the DMRs, GRanges objects were constructed for genes, 5′ and 3′ basal regulatory regions, coding and non-coding exons, introns, TEs, and CNEs. GC content for all feature types was computed with the BSgenome::alphabetFrequency function.

### Relating DMRs to genomic features and nullDMR generation

Distances between DMRs and all genes on their respective scaffolds were computed using the IRanges::distance function. Gene counts and distances in different sized windows around DMRs were determined by filtering these results with various distance thresholds. Overlaps between DMRs and all genomic feature types were found using IRanges::findOverlap, as were overlaps between features, for example lncRNAs and protein-coding genes.

To test the significance of these results “nullDMRs” were generated with the following procedure. First, genome scaffold names were sampled with replacement 709 times using the number of DMRs on each scaffold as probability weights. Then, the actual DMR widths were randomly assigned to this new list of scaffold names and used to generate random genomic intervals, for example if scaffold_100 was assigned one DMR with width = 200, then one nullDMR was defined as a randomly chosen 200 bp interval somewhere on scaffold_100. The end result was a list of 709 genomic intervals that 1) were positioned across scaffolds with a similar density as the real DMRs, and 2) had the same width distribution as the DMRs. This process was repeated to create 10,000 lists of 709 nullDMRs each, and distances/overlaps to genes and other features were computed for each nullDMR list. *P*-values were defined as the fraction of nullDMR sets that met or exceeded whatever DMR attribute we were testing, and if none did we reported *p* < 1e-4. For example, 15 DMRs overlapped > 1 gene, and 15 or more nullDMRs overlapped > 1 gene 1498/10,000 times, so *p* = 0.1498 (Table 1A).

### General statistics and visualization

The bsseq::dmrFinder function returns statistics about each DMR: *n* (number of smoothed CpGs, i.e. those with enough coverage to be included in the analysis), *width* (size of interval, bp), *invdensity* (mean distance between CpGs, bp), *areaStat* (sum total of t-statistics), *maxStat* (most extreme t-statistic value), *group1.mean* (mean smoothed methylation value for ND fish), *group2.mean* (mean smoothed methylation value for D fish), *meanDiff* (mean difference of smoothed methylation values, ND-D), *tstat.sd* (standard deviation of t-statistics). We also computed overall GC content, mean methylation level across all four fish, and log2(D/ND) mean methylation values for each DMR. The following were computed for every gene body, exon, intron, and basal regulatory region in the *A. burtoni* genome: size, GC content, and the numbers of overlapping TEs, CNEs, and DMRs. We also counted the number of isoforms for each gene, computed expression levels and fold-difference in D vs ND fish (see above), and kept track of whether the signs of expression and methylation fold-difference were the same.

All comparisons of these statistics across subsets of DMRs or genes were performed in R using the Mann-Whitney *U* test, or the Kruskal-Wallis extension when more than two groups were tested, as were comparisons of average gene counts and distances across different window sizes around DMRs/nullDMRs (stats::kruskal.test function). All *p*-values are uncorrected unless otherwise stated. Tests of categorical associations between subsets of DMRs or genes were performed with Fisher’s exact test, for example Table 2 (stats::fisher.test). In some cases, p-values for these tests were either Bonferroni-corrected for the number of total tests, for example testing for overlap between DMR genes in 14 enriched KEGG pathways and 6 clusters of enriched GO terms, or adjusted using the less conservative Benjamini-Hochberg (BH) procedure (stats::p.adjust), for example testing for enrichment of DMR genes in one of 33 hypothalamic cell-type clusters. For correlations, Pearson’s *r* was used when two variables were similarly scaled or one of the variables was a principal component (*p*-values via Fisher’s *Z*-transformation), otherwise Spearman’s *rho* was used (p-values via algorithm AS 89 or asymptotic *t* approximation, stats::cor.test). Principal components of the correlations between DMRs (based on the types of genomic features they overlapped, Fig. 3) and GO terms (based on which DMR genes they contained, Fig. 5) were computed using the stats::prcomp R function. The input correlation matrices were scaled and centered first.

Most plots were initially made in R and all were edited using Adobe Illustrator (Adobe Systems, San Jose, CA). Smoothed and raw methylation ratios were visualized using the bsseq::plotManyRegions function (Fig. 1). Average numbers of genes around DMRs were plotted as a function of average gene-DMR distance across different window sizes using ggplot2 [165] (Additional file 1: Figure S5). All other line and scatter plots were made using graphics::plot, fit-lines for correlations were computed using stats::lm and plotted with graphics::abline, box-and-whisker plots and barplots were made with graphics::boxplot and graphics::barplot, heatmaps and dendrograms were made with stats::heatmap and stats::hclust, and venn diagrams were made with functions in the VennDiagram R package [166]. Two types of visualizations were not constructed in R: KEGG pathway diagrams were downloaded as .xml files from the KEGG website [167] then edited in Cytoscape [168] and Illustrator, and gene/DMR schematics (Fig. 8) were exported as .svg files from Integrated Genomics Viewer [169, 170] then edited in Illustrator.

### Functional enrichment analysis of DMR genes

Functional enrichment analysis of the DMR genes was performed using Entrez gene identifiers for humans to maximize exploratory power. Since systematic mappings between *A. burtoni* and human gene ids were not available at the time of this analysis we performed a reciprocal blast procedure to generate high-confidence *A. burtoni*-human mappings for as many genes as possible. First, we used blastx to compare the *A. burtoni* transcriptome [171] to the proteomes of five other well-annotated fish species (*Danio rerio*, *Gasterosteus aculeatus*, *Oreochromis niloticus*, *Oryzias latipes*, *Tetraodon nigroviridis*) acquired from Ensembl [172], then blastp to compare the best protein hit for each transcript back to the *A. burtoni* proteome [173]. When the best hit for this reciprocal blast was the protein made by the original *A. burtoni* transcript we called it a successful hit, then used the Ensembl id of the protein in the other fish species to search for a human homolog using functions in the biomaRt R package [174, 175]. This yielded human Entrez ids for > 20,000 of the genes in the *A. burtoni* annotations. All *A. burtoni* genes that successfully mapped to a human Entrez id were used as background in functional enrichment tests. In cases where a single *A. burtoni* gene mapped to multiple human ids (< 5% of mappings) we used the human Entrez id with the lowest numeric value. The Fernald lab github repository contains Python and R scripts and instructions for implementing this procedure with any species [176].

Gene ontology (GO) analysis was performed in R using the GOFunction package [177], and molecular pathways from the Kyoto Encyclopedia of Genes and Genomes (KEGG) database were screened for DMR genes using the enrichKEGG function in the clusterProfiler package [178] (Additional file 2: Table S5). GO categories and KEGG pathways with *p* < 0.1 after adjustment via the Benjamini-Yekutieli (BY) or Benjamini-Hochberg (BH) procedure were considered “significantly” enriched, i.e. compelling enough to report. GOFunction performs a standard gene ontology analysis where the significance of overlap between input genes and GO terms is quantified using the hypergeometric test. We chose it over other GO analysis tools because it also culls the list of significant terms to reduce redundancy resulting from 1) ancestor-offspring term relationships, and 2) non-ancestor-offspring terms with overlapping genes.

We also used the WEB-based Gene Set Analysis Toolkit [179] to screen DMR genes for molecular interactions in the Biological General Repository for Interaction Datasets (BioGRID) [180] and disease associations with the Gene List Automatically Derived For You (Glad4U) tool [181]. Only one molecular interaction set was significant after BY-correction (1742:DLG4, see Results section on glutamatergic synapses, Additional file 2: Table S7), but 50 others were significant before correction and were used throughout as context for other results. Fourteen disease categories were enriched at BY-adjusted *p* < 0.05 (Additional file 2: Table S9).

### Enrichments for cell-type markers in the DMR genes

We tested for DMR genes in two sets of cell-type marker lists that were generated using single-cell RNA-seq data from mice. Zhang et al. [56] identified markers for subtypes of glia, neurons, and vascular cells in mouse cerebral cortex based on relative overexpression in specific cell-types. We downloaded expression values for astrocytes, neurons, oligodendrocyte precursor cells, newly formed oligodendrocytes, myelinating oligodendrocytes, microglia, and endothelial cells from the web [182]. Following [56] (Materials and Methods: Analysis of cell type-enriched genes, transcription factors, signaling pathways, and metabolic pathways), we defined markers as genes with > 5 FPKM expression levels that were > 5x higher in one cell-type compared to their mean expression across all other types. The resulting lists were > 99% mutually exclusive.

In contrast to overexpression, Chen et al. [57] used unbiased clustering to detect cell-type specific coexpression signatures in mouse hypothalamus that corresponded to ependymal cells, tanycytes, six stages of oligodendrocyte development, 15 types of glutamatergic neurons, and 18 types of GABAergic neurons. Genes were often present in multiple clusters that each corresponded to a subtype of a specific kind of cell, for example GABAergic neurons. For this reason, we prefer to designate cell-type-specific coexpression signatures, i.e. clusters, rather than individual gene markers. Tables of gene-cluster mappings were downloaded as supplementary data from the web. We also merged all GABAergic and glutamatergic clusters into lists representative of these two classes of neurons in the hypothalamus, and created some additional clusters based on Chen et al.’s assessment of their data: neurosecretory cells (Glu10–15), suprachiasmatic nucleus cells (GABA8–9), arcuate nucleus cells (Glu11,13 and GABA11,12,15), Gnrh cells (Glu10, GABA11), and cells containing genes that were differentially expressed after food deprivation (Glu5,8,12 and GABA1,11,15,18).

Identifiers for genes of interest from both datasets were converted from gene symbols to human Entrez ids using functions in the biomaRt R package [174, 175], then compared to the DMR genes using Fisher’s exact test. When markers/clusters were compared to multiple subsets of DMR genes, for example enriched GO terms, *p*-values were BH-corrected for the number of subsets tested.

## Additional files

Additional file 1: **Figure S1.** Plasticity across multiple biological levels in Astatotilapia burtoni. **Figure S2.** Descriptive statistics across all DMRs. **Figure S3.** Methylation levels and variability by genome scaffold in D versus ND fish. **Figure S4.** Comparisons of descriptive statistics in D-DMRs versus ND-DMRs. **Figure S5.** Distances between DMRs and genes across different genome scaffold sizes. **Figure S6.** DMR statistics as a function of the number of genes within different distances. **Figure S7.** DMR counts in different combinations of genomic features. **Figure S8.** Expression fold-difference and isoforms of DMR genes depending on location and sign of DMR. **Figure S9.** DMR gene properties compared to other genes. **Figure S10.** Enriched biological functions in subsets of DMR genes. **Figure S11.** DMR genes in glutamatergic and GABAergic synapses. **Figure S12.** DMR genes involved in axon guidance and oligodendrocyte progenitor cell development. **Figure S13.** Properties of DMR genes in development (Dev) versus neural activity (Act) GO term clusters. **Figure S14.** Correlations between gene properties and number of TEs they contain. **Figure S15.** M-bias plots before and after trimming BS-Seq read pairs. **Figure S16.** No relationship between genome scaffold length and DMR statistical properties. (PDF 4380 kb)

Additional file 2: **Table S1.** Characteristics of individual DMRs and DMR genes. **Table S2.** Multi-gene DMRs and multi-DMR genes. **Table S3.** Differentially expressed genes within 50kb of a DMR. **Table S4.** DMR genes overlapping lncRNAs. **Table S5.** GO categories and canonical molecular pathways enriched in the DMR genes. **Table S6.** DMR genes associated with cell-type markers in other studies. **Table S7.** Molecular interactions enriched in the DMR genes. **Table S8.** Functions enriched in DMR genes oligodendrocyte progenitor cell coexpression clusters. **Table S9.** Disease-associated genes enriched in the DMR genes. **Table S10.** BS-Seq information. (XLSX 925 kb)

## Data Availability

The datasets generated and analyzed during the current study are available in the Sequence Read Archive database (SRP142604) and are accessible through NCBI BioProject ID PRJNA453533.

## Abbreviations

Act: Gene ontology terms related to neural structure and activity
CNE: Conserved non-coding element
D: Dominant social status
D-DMR: Differentially methylated region with higher methylation levels in dominant fish
Dev: Gene ontology terms related to development and cell differentiation
DMR: Differentially methylated region
GnRH: Gonadotropin-releasing hormone
GO: Gene ontology
lncRNA: long non-coding RNA
ND: Non-dominant social status
ND-DMR: Differentially methylated region with higher methylation levels in non-dominant fish
OPC: Oligodendrocyte progenitor cell
TE: Transposable element
TSS: Transcription start site
VGCC: Voltage-gated calcium channel
