# Supplementary material for: Genome-wide effects of social status on DNA methylation in the brain of a cichlid fish, Astatotilapia burtoni
Source: BMC Genomics. 2019 Sep 11;20:699. doi: 10.1186/s12864-019-6047-9 (PMC6737626; doi:10.1186/s12864-019-6047-9)
Supplement: Supplementary file 1 — Figure S1. Plasticity across multiple biological levels in Astatotilapia burtoni. Figure S2. Descriptive statistics across all DMRs. Figure S3. Methylation levels and variability by genome scaffold in D versus ND fish. Figure S4. Comparisons of descriptive statistics in D-DMRs versus ND-DMRs. Figure S5. Distances between DMRs and genes across different genome scaffold sizes. Figure S6. DMR statistics as a function of the number of genes within different distances. Figure S7. DMR counts in different combinations of genomic features. Figure S8. Expression fold-difference and isoforms of DMR genes depending on location and sign of DMR. Figure S9. DMR gene properties compared to other genes. Figure S10. Enriched biological functions in subsets of DMR genes. Figure S11. DMR genes in glutamatergic and GABAergic synapses. Figure S12. DMR genes involved in axon guidance and oligodendrocyte progenitor cell development. Figure S13. Properties of DMR genes in development (Dev) versus neural activity (Act) GO term clusters. Figure S14. Correlations between gene properties and number of TEs they contain. Figure S15. M-bias plots before and after trimming BS-Seq read pairs. Figure S16. No relationship between genome scaffold length and DMR statistical properties. (PDF 4380 kb) [file 12864_2019_6047_MOESM1_ESM.pdf]

Additional file 1

This file contains Supplementary figures S1-16 and their legends, as well as the legends for Supplementary tables S1-10 (see Additional file 2 for the actual tables).

Legends for Supplementary figures S1-16

Figure S1: Plasticity across multiple biological levels in *Astatotilapia burtoni*

A: *A.burtoni* are endemic to Lake Tanganyika, east Africa where they live in shore pools, estuaries and tributaries as well as the lake. A typical shore pool colony in Lake Tanganyika, Burundi, Africa. Solid dots are grid stakes spaced ~50 cm and labeled (1–4; A–D) for identification during observations. Circles represent spawning pit locations of dominant (D) males that are brightly colored, defend spawning sites, and can reproduce. Lighter colored outlines circumscribe the approximate territories of individuals. Non-dominant (ND) males that have pale body coloration school together with similarly colored females near the territorial area. (Based on [34]).

B: Phenotypic characteristics of reproductively active D males (top) and socially suppressed ND males (bottom). D males have larger GnRH1 neurons (red; immunohistochemical staining) in the preoptic area of the hypothalamus and larger testes compared to NDs. (Based on [39]).

C: Timing of the changes in behavior, body color, hypothalamic gene expression, and GnRH1 cell size as a ND male ascends to D social status, as observed across a number of studies (see [39]). Arrows indicate the time point after social opportunity at which the first significant increase from stable ND male values was observed. Transcriptional changes (e.g., as determined by in situ hybridization or qRT-PCR) are indicated in red. *ARα/β*, androgen receptor subtypes; *egr-1*, early growth response factor-1; *ERβa/βb*, estrogen receptor subtypes; *GnRH1*, gonadotropin-releasing hormone 1; *kiss1r*, kisspeptin receptor 1. (Based on [39]).

Figure S2: Descriptive statistics across all DMRs

Histograms of DMR statistics with the mean and median of each distribution reported above the plot and represented by vertical solid and dashed red lines, respectively. All values for individual

DMRs are reported in Additional file 2: Table S1. During the DMR identification process, all DMRs with n<3 or meanDiff.abs<0.1 were filtered out. **n:** Number of CpGs within DMR that had at least 4x coverage in every fish. **width:** Number of total base pairs within DMR. **invdensity:** Average distance between 4x coverage CpGs within DMR, i.e. ratio of width/n. **GC:** Percentage of all base pairs within DMR that were guanines (G) or cystines (C). **all.mean:** Average of smoothed methylation levels within DMR, averaged across all fish, i.e. the mean of Figure 2A-B. Referred to as "baseline" methylation throughout the manuscript. **areaStat:** Sum of all t-statistic values for 4x coverage CpGs within DMR. **areaStat.abs:** Absolute values of areaStat. **maxStat:** Maximum t-statistic value across all 4x coverage CpGs within DMR. **maxStat.abs:** Absolute values of maxStat. **meanDiff.abs:** Absolute value of mean methylation difference (Figure 2C) **log2fc.abs:** Absolute value of methylation fold-difference (Figure 2D).

Figure S3: Methylation levels and variability by genome scaffold in D versus ND fish

Comparisons of the coefficient of variation (**A, C**; CV) and median (**B, D**) of smoothed methylation levels for every genome scaffold in D (green) versus ND (blue) fish. P-values above boxplots (**A, B**) are from the Mann-Whitney U test. Top and bottom of boxes represent the first and third quartiles, respectively, whiskers extend to the most extreme data points no more than 1.5 times the interquartile range from the box, and data points beyond the whiskers are not shown to improve visibility. Each dot in the scatterplots (**C, D**) represents a scaffold and values are the same as those compared in boxplots. Pearson correlations with Student asymptotic p-values are reported above plots, with best fit regression lines shown in red.

Figure S4: Comparisons of descriptive statistics in D-DMRs versus ND-DMRs

Boxplots comparing descriptive statistics from Additional file 1: Figure S2 and Additional file 2: Table S1 of DMRs where D fish had higher methylation levels (D-DMRs, green) versus DMRs where ND fish had higher methylation levels (ND-DMRs, blue). P-values are from the Mann-Whitney U test and median values are reported under each box after the group name. Top and bottom of boxes represent the first and third quartiles, respectively, whiskers extend to the most extreme data points no more than 1.5 times the interquartile range from the box. Only the absolute values of areaStat, maxStat, meanDiff, and log2fc were compared because these

statistics have opposite signs in D-DMRs and ND-DMRs by definition, thus the comparisons would have been trivial.

Figure S5: Distances between DMRs and genes across different genome scaffold sizes

A: Average number of genes (y-axis) plotted as a function of their average distance to DMRs (x-axis) for increasing window sizes (dots, 0-100kb in 2kb increments). Error bars on each dot represent the standard error around the mean on each axis.

B-C: Average number of genes (y-axis) within different window sizes around DMRs (x-axis). Different colored lines represent increasingly larger minimum genome scaffold sizes, with color key to right of **C**. For example, the black line represents minimum scaffold size of 0bp, i.e. all scaffolds, while the darkest blue line represents gene counts around DMRs only on scaffolds that are at least 5mb long (n=8). **C** is the same as **B**, except zoomed in to the lower left quadrant defined by the grey lines at y=10 and x=1e5 in **B** for better visibility.

Figure S6: DMR statistics as a function of the number of genes within different distances

A: Significance level (y-axis) of average gene number (blue dots) and distance to DMRs (green dots) plotted as a function of window size (x-axis). Significance was defined as the fraction of all 10,000 nullDMR sets (p-value) that averaged at least as many genes, at as close of a distance, as the real DMRs. -log10(p-values) are plotted here so that more significant results are higher on the y-axis and horizontal red lines denote values equivalent to p=0.05 and p=0.01. The nullDMRs never had as many genes around them as the real DMRs in windows up to 10kb, so for these tests p=0 and -log10(0)=positive infinity.

B: Fraction of DMRs (y-axis) that had more than the mean number of genes in a given window size (x-axis) around them. Numbers along the line represent the average gene count for their respective window sizes.

C: -log10(p-values) (y-axis) plotted against window size (x-axis) from tests comparing the descriptive statistics of DMRs with more versus less than the mean number of genes in a given window size. Dotted lines represent different statistics from Additional file 1: Figure S2 and Additional file 2: Table S1: log2fc.abs (green), meanDiff.abs (purple), n, width, invdensity, areaStat.abs, and the ratio of D- to ND-DMRs (all grey). All p-values from Mann-Whitney U tests

except the D-DMR/ND-DMR ratio comparison, which was done with Fisher's exact test. Only log2fc.abs and meanDiff.abs ever surpass -log10(p=0.05).

Figure S7: DMR counts in different combinations of genomic features

A: DMRs in gene bodies and/or regulatory regions, which were defined as 5kb up- to 1kb downstream of the 5' end, or 1kb up- to 5kb downstream of the 3' end.

B: DMRs outside of gene bodies that were within 5kb up- or downstream of genes (5' and 3' type-1 regulatory region DMRs), transposable elements (TEs), and/or conserved non-coding elements (CNEs). Three different types of DMRs in regulatory regions are schematized in Figure 1D in the main text. Type-1: within 5kb of a gene but outside the body. Type-2: overlapping any of the first or last 1kb of a gene. Type-3: a type-1 or type-2 DMR for one gene that was also within the body of another gene on the opposite strand.

C: Same DMRs as in **A**, but with gene body DMRs divided into those in introns and/or exons and regulatory region DMRs divided into type-1 and type-2. The type-3 designation is ignored here since by definition any type-3 regulatory region DMRs are in the body of a different gene.

D: All type-2 and type-3 regulatory region DMRs.

Figure S8: Expression fold-difference and isoforms of DMR genes depending on location and sign of DMR

A-B: Boxplots show gene expression fold-difference, defined as log2 of D/ND TPM (**A**), and number of isoforms for DMR genes (**B**), depending on whether DMRs were in the body and/or 5' or 3' regulatory regions. Horizontal grey lines in each plot denote the overall median for all data points.

C: Boxplot comparing gene expression fold-difference in genes colored red (same sign) versus blue (opposite sign) in **E**. Those with expression and methylation fold-differences with different signs had lower expression in D versus ND fish. Horizontal grey line marks 0 on the y-axis for reference.

Omnibus p-values from the Kruskal-Wallis (**A-B**) or Mann-Whitney (**C**) test are reported above each boxplot and group sizes are reported in parentheses after the group name under each box. Top and bottom of boxes represent the first and third quartiles, respectively, whiskers

extend to the most extreme data points no more than 1.5 times the interquartile range from the box. Data points beyond this range are not shown in **C** to increase visibility.

*p<0.05, ***p<0.001, ****p<0.0001

Figure S9: DMR gene properties compared to other genes

Boxplots comparing genes with a DMR in their body and/or regulatory region to all other genes on genome scaffolds analyzed with BSmooth (see Methods): average expression in transcripts per million (TPM) across all fish (**A**), the absolute value of log2 D/ND expression fold-difference (**B**), log2 D/ND expression fold-difference (**C**), number of splice variants (**D**), GC content (**E**), and number of transposable elements (TEs) in the gene body (**F**). Mann-Whitney p-values are reported above each plot and numbers of genes in each category are in parentheses below the group names. Top and bottom of boxes represent the first and third quartiles, respectively, whiskers extend to the most extreme data points no more than 1.5 times the interquartile range from the box. Horizontal grey lines denote the median value of each quantity across all genes.

Figure S10: Enriched biological functions in subsets of DMR genes

Venn diagrams showing enriched gene ontology categories (BY-adjusted p<0.1) for DMR genes that overlapped a lncRNA (**A**), had at least one duplicate in the *A.burtoni* genome (**B**), were transcription factors (**C**), and/or are known to interact with nuclear receptors (**D**).

A-B: Italicized text denotes genes with more than one DMR and black boxes highlight duplicated genes where more than one copy had a DMR. Bold text indicates that the gene and an antisense lncRNA were overlapped by a DMR (**A**), or the gene had an antisense lncRNA but the lncRNA itself was not necessarily overlapped by a DMR (**B**).

C: Distribution of transcription factor DMR genes across the Dev1, Dev2, and Act2 GO term clusters. Bold text indicates that the gene's DMR includes a conserved non-coding element (CNE) and italics indicate that the gene is also present in one of the other Act GO term clusters. Black boxes with solid lines surround genes involved in steroid hormone signaling and boxes with dashed lines denote genes involved in cell fate determination.

D: Distribution of nuclear receptor-related DMR genes across GO categories that were specifically enriched in these genes. Bold text indicates genes involved in steroid hormone

mediated signaling and italics indicate the one gene here with a subDMR (*abl1*). The dashed box surrounds genes found in histone deacetylase complexes.

Figure S11: DMR genes in glutamatergic and GABAergic synapses

A-B: Schematics of the glutamatergic (**A**) and GABAergic (**B**) synapse KEGG pathways, adapted from visualizations of these pathways on the KEGG website [167]. Each rectangle represents a gene (e.g. *dlg4* in **A**) or family of genes (e.g. KA in **A** represents all kainate receptors). Blue, green, and grey indicate genes/families with ND-DMRs, D-DMRs, and no DMRs, respectively. KA, SHANK (**A**), and GABAA (**B**) are colored blue and green in proportion to how many genes in each family had higher DMR methylation levels in ND or D fish, respectively. Red rectangles highlight molecules that did not necessarily have DMRs themselves, but are known to interact with multiple DMR genes (see Additional file 2: Table S7). Glutamate receptor genes: *gria4*(*102292924*), *grid1(102294093), grik2(102291713), grik4, grik5(102301729), grin2a, grin3b(102310445), grm6(102298439).* GABA receptor genes: *gabra5, gabbr1(102294658), gabbr2*, *gabrb3, gabrg3, gabrp(102297141).* Complete lists of all DMR genes in each KEGG pathway can be found in Additional file 2: Table S5B.

Figure S12: DMR genes involved in axon guidance and oligodendrocyte progenitor cell development

A: Schematic of the Axon guidance KEGG pathway, adapted from visualization of this pathway on the KEGG website [167]. All coloring and naming conventions match those in Figure S11.

B: Venn diagram of DMR genes that interact with signaling molecules in the axon guidance pathway (SRC, CDC42, RHOA, FYN), as indicated by red rectangles in **A**. Genes that are bolded also interact with NCK1, also highlighted in **A**, along with two other axon guidance pathway DMR genes, *erbb4* and *adgrl2*. See Additional file 2: Table S7.

Figure S13: Properties of DMR genes in development (Dev) versus neural activity (Act) GO term clusters

Boxplots show comparisons between DMR genes grouped by whether they were part of the Dev and/or Act GO term clusters, including gene size (**A**), gene GC content (**B**), number of transposable elements (TEs) in the gene body (**C**), gene expression in transcripts per million

(TPM) (**D**), average methylation levels in DMRs across all fish, i.e. all.mean in Additional file 1: Figure S2 and Additional file 2: Table S1 (**E**), the absolute values of gene expression (**F**) and methylation (**G**) fold-differences across social status, defined as log2(D/ND), i.e. log2fc.abs in Additional file 1: Figure S2 and Additional file 2: Table S1, whether the two kinds of fold-differences had opposite signs (**H**), the ratio of methylation to expression signed fold-differences (**I**), and the absolute value of the difference between ND and D methylation in DMRs, i.e. meanDiff in Figure S2 and Table S1 (**J**). In **K**, the same fold-difference ratio is plotted as in **I,** except for every individual GO term.

Figure S14: Correlations between gene properties and number of TEs they contain

Heatmap representing Spearman correlations of the number of transposable elements (TEs) contained in gene bodies with other gene properties (rows), including those of their DMRs. Correlations were computed within all genes (column 1), just DMR genes (column 2), DMR genes grouped by enriched gene ontology (GO) category/term (column 3), and DMR genes grouped by cluster of enriched GO terms (column 4). For columns 3 and 4, gene TE number and other properties were averaged across all genes in each term or term cluster. Spearman's rho values are shown in cells where the correlation p-value passed Bonferroni-correction for this 13x4 matrix of correlations. Gene properties included the following. **TPM:** expression level in transcripts per million. **%GC (DMR/gene):** ratio of GC content within the DMR compared to the GC content of the entire gene. **%AntiCorFC:** whether gene expression and DMR methylation fold-differences had different signs. For the DMR genes column, TE number was correlated to a vector of 1's and 0's, where each gene with opposite-signed fold-differences was assigned a 1. For the GO terms and GO term clusters columns, the mean of these 1's and 0's for genes in a given term or term cluster worked out to be the percentage of genes with opposite-signed fold-differences. **ND-D meth. (abs):** absolute value of the difference between ND and D methylation in DMRs, i.e. meanDiff.abs from Additional file 1: Figure S2 and Additional file 2: Table S1. **Baseline methylation:** average of DMR methylation levels across all fish, i.e. all.mean from Figure S2 and Table S1. **%GC (DMR):** DMR GC content. **ND-D methylation:** difference between ND and D methylation in DMRs, i.e. meanDiff from Additional file 1: Figure S2 and

Additional file 2: Table S1. **log2(D/ND) meth. (abs):** absolute value of the log2 of D/ND DMR methylation levels, i.e. log2fc.abs from Additional file 1: Figure S2 and Additional file 2: Table S1. **isoforms:** number of splice variants. **log2(D/ND) methylation:** log2 of D/ND DMR methylation levels, i.e. log2fc from Additional file 1: Figure S2 and Additional file 2: Table S1. **%GC (gene):** gene GC content. **log2(D/ND) TPM:** log2 of D/ND expression levels. **log2(D/ND) TPM (abs):** absolute value of log2 of D/ND expression levels.

Figure S15: M-bias plots before and after trimming BS-Seq read pairs

M-bias plots show methylation levels (y-axis) as a function of base position (x-axis) in BS-Seq read pairs from each fish, both before (**A**) and after (**B**) trimming the first and last 3 bases from each read. Top left of each plot has the fish ID number, as reported in Additional file 2: Table S10.

Figure S16: No relationship between genome scaffold length and DMR statistical properties

Scatterplots show the relationships between genome scaffold length (x-axis) and descriptive statistics for the DMRs from Additional file 1: Figure S2 and Additional file 2: Table S1 (y-axes). Each dot represents a DMR and they are colored to represent whether methylation was higher in D (green, D-DMRs) or ND (blue, ND-DMRs) fish. Spearman correlations are reported above each plot.

Legends for Supplementary tables S1-10

Table S1: Characteristics of individual DMRs and DMR genes

A: Values of the statistics shown in Figure 2 and Additional file 1: Figure S2 (columns) for each of the 709 DMRs (rows).

B: All genes with a DMR(s) in their body and or regulatory region (rows). **Columns A-C:** different identifiers for the genes. **Columns D-M:** other gene information, including whether it is protein coding or a lncRNA (biotype), which genome strand it is on (strand), GC content (GC), how many splice variants it has (isoforms), average expression level across all fish in transcripts per million (expression(TPM)), the log2(D/ND) fold-difference in expression as computed by

DESeq2 (expression_log(D/ND)) , number of transposable elements in its body (TE.num), whether it is a transcription factor (TF), whether it has at least one duplicate copy elsewhere in the genome (Duplicated), number of DMRs it had (dmr.num). **Columns N-W:** information about the DMRs, including genomic position (DMRs), GC content (dmrGC), its location within the gene (DMR_locations), mean methylation across all fish (mean_methylation), mean difference in ND-D methylation, i.e. same as meanDiff in Figure 2C, Additional file 2: Table S1A (meanDiff), log2 (D/ND) methylation fold-difference (methylation_log2(D/ND)), whether the DMR overlaps a TE or CNE (dmrOV.te, dmrOV.CNE), and potential transcription factor binding sites contained within the DMR, followed by the score of the hit (dmrOV.tfbs).

C: Table showing whether DMRs (rows) overlapped a transposable element, and if so, its type and location (**TE**), or conserved non-coding element (**CNE**), and if so the bit score of the alignment (**score**).

Table S2: Multi-gene DMRs and multi-DMR genes

A: DMRs that overlapped the body and/or regulatory region of more than one gene. Rows are genes and the first 8 columns report the following gene properties. **geneLOC:** official locus identifier assigned by NCBI genome annotation pipeline. **geneSymbol:** HGNC-style gene acronym, some assigned by NCBI, some are HGNC symbols for closest human homolog. **geneName:** full gene description assigned by NCBI. **biotype:** whether the gene codes for a protein or is considered a lncRNA, geneSymbol and geneName are not applicable (NA) for lncRNAs. **strand:** whether the gene is on the forward (+) or reverse (-) strand of DNA. **isoforms:** number of mRNA splice variants assigned by NCBI. **expression(TPM):** expression level in transcripts per million (TPM) calculated using Kallisto [151]. **expression_log2(D/ND):** log2 fold-difference of expression in dominant (D) compared to nondominant (ND) fish computed using DESeq2. The remaining columns report DMR information. **DMR:** genome span covered by the DMR in format scaffold_number:start-end. **DMR_location:** combination of gene features overlapped by DMR, “cds" and “exon” refer to protein-coding and non-coding exons, respectively. **mean_methylation:** average methylation levels within the DMR across all fish, i.e. all.mean from Additional file 1: Figure S2 and Additional file 2: Table S1. **meanDiff:** difference of

average ND - D methylation levels in the DMR, also in Additional file 1: Figure S2 and Additional file 2: Table S1. **methylation_log2(D/ND):** log2 fold-difference of methylation levels in D compared to ND fish, i.e. log2fc in Additional file 1: Figure S2 and Additional file 2: Table S1.
B: Genes that had more than one DMR in their body and/or regulatory regions. Rows are genes and columns are the same as in **A**, with the addition of **numDMR:** number of DMRs a given gene had in its body and/or regulatory region. DMR_location reflects the locations of all DMRs for a given gene, and the values in mean_methylation, meanDiff, and methylation_log2(D/ND) are averaged across all DMRs for the gene. Thus, genes where the absolute value of meanDiff<0.1 had a mix of D- and ND-DMRs that changed roughly the same amount, since DMRs with meanDiff.abs<0.1 were filtered out.

Table S3: Differentially expressed genes within 50kb of a DMR

Three genes that were differentially expressed (DE) in D versus ND fish had a DMR in their body and/or regulatory region (rows 3-5) and four others had a DMR within 50kb (rows 6-9). Columns 1-3 (**DE gene**) contain information about the DE genes. **name:** gene symbol. **adj.pval:** adjusted p-value from DESeq2 [152] test for differential expression. **log2.fc:** log2 of D/ND expression, computed using DESeq2. Columns 4-6 (**DMR**) contain information about the relevant DMRs. **id:** genome scaffold and position. **location:** location relative to DE gene. **log2.fc:** log2 of D/ND methylation. Columns 7-8 (**Other genes within 50kb of DMR**) contain information about other genes that were within 50kb **upstream** or **downstream** of the DE gene DMRs. Gene symbols are followed by distance to the DMR and expression log2.fc in parentheses. Some gene symbols are assigned by the NCBI annotation pipeline (e.g. *ubxn4*) while others are the symbols for the best human homolog. All LOC names refer to lncRNAs.

Table S4: DMR genes overlapping lncRNAs

A: All genes (rows) with a DMR in their body and/or regulatory region that also overlapped a lncRNA, with 34/39 genes in antisense orientation with the lncRNA. Columns A-O contain information about the DMR genes, while columns P-AB contain the same information for the lncRNA overlapped by the DMR gene. **geneLOC:** official locus identifier assigned by NCBI genome annotation pipeline. **geneSymbol:** HGNC-style gene acronym, some assigned by

NCBI, some are HGNC symbols for closest human homolog. **geneName:** full gene description assigned by NCBI. **scaffold:** genome scaffold containing the gene. **start/end:** position of gene on the scaffold. **strand:** whether the gene is on the forward (+) or reverse (-) strand of DNA. **isoforms:** number of mRNA splice variants assigned by NCBI. **expression(TPM):** expression level in transcripts per million (TPM) calculated using Kallisto [151]. **expression_log2(D/ND):** log2 fold-difference of expression in dominant (D) compared to nondominant (ND) fish computed using DESeq2 [152]. The remaining columns report DMR information. **DMR:** genome span covered by the DMR in format scaffold_number:start-end. **DMR_location:** combination of gene features overlapped by DMR, “cds" and “exon” refer to protein-coding and non-coding exons, respectively. **mean_methylation:** average methylation levels within the DMR across all fish, i.e. all.mean from Additional file 1: Figure S2 and Additional file 2: Table S1. **meanDiff:** difference of average ND - D methylation levels in the DMR, also in Additional file 1: Figure S2 and Additional file 2: Table S1. **methylation_log2(D/ND):** log2 fold-difference of methylation levels in D compared to ND fish, i.e. log2fc in Additional file 1: Figure S2 and Additional file 2: Table S1.

Table S5: GO categories and canonical molecular pathways enriched in the DMR genes

A: GO categories (rows) that contained significant numbers of DMR genes. Columns contain information about each category: parent GO hierarchy (**ontology;** BP: Biological process, CC: Cellular component, MF: Molecular function), unique identifier (**goid**) and name (**name**), total number of background genes in the category (**refnum**), total number of DMR genes in the category (**interestnum**), raw hypergeometric p-value (**p-value**), Benjamini-Yekutieli (BY) adjusted p-value (**adjustp**), and the *A.burtoni* identifiers for DMR genes in the category (**genes**). GO enrichments were identified using the GOFunction R package [177] (see Methods).

B: KEGG molecular pathways (rows) that contained significant numbers of DMR genes. Columns contain information about each pathway: unique identifier for the KEGG database (**ID**) and name of the pathway (**Description**), ratio of DMR genes in the pathway to DMR genes in the KEGG database (**GeneRatio**), ratio of all genes in the pathway to all background genes in the KEGG database (**BgRatio**), raw p-value (**pvalue**), Benjamini-Hochberg adjusted p-value

(**p.adjust**), and the *A.burtoni* identifiers for DMR genes in the pathway (**geneID**). KEGG enrichments were identified using the enrichKEGG function in the clusterProfiler R package [178] (see Methods).

C: Core set of DMR genes that were found in 15-20 enriched GO terms from **A** and/or at least 3 KEGG pathways from **B**. Italicized genes had multiple DMRs and underlined genes overlapped a lncRNA. *in 15-20 GO terms and 3 KEGG pathways, **in 9 KEGG pathways.

Table S6: DMR genes associated with cell-type markers in other studies

Table showing DMR genes (rows) that were either overexpressed in a certain cell type from [56] **Columns N-T**), or in a cell type coexpression cluster from [57] (**Columns U-X**). **Columns A-M:** Same as previous supplemental tables, e.g. Additional file 2: Table S1.

Table S7: Molecular interactions enriched in the DMR genes

Categories of molecular interactions (rows) from the Biological General Repository for Interaction Datasets (BioGRID) [180] that were in enriched in the DMR genes. Columns are the names of molecules that have significantly enriched interactors in the DMR genes (Term), number of DMR genes that interact with the molecule (Count), uncorrected hypergeometric p-value of the enrichment as computed with WebGestalt [179] (PValue), DMR gene interactors, ids match geneSymbol column in Additional file 2: Table S1B (Genes), total number of DMR genes in the BioGRID database (List.Total), total number of genes from the database that interact with the given molecule (Pop.Hits), total number of *A.burtoni* genes in the BioGRID database, Benjamini-Yekutieli (BY) corrected p-value (Benjamini).

Table S8: Functions enriched in DMR genes oligodendrocyte progenitor cell coexpression clusters

Gene ontology results from just DMR genes that were in one of the OPC clusters from [57]. Rows are enriched GO categories and columns are the same as Additional file 2: Table S5A, except “Figure 7C category”, which references where genes from a given GO term are represented in the Venn diagram in Figure 7C.

Table S9: Disease-associated genes enriched in the DMR genes

Results from comparing the DMR genes against the disease database from Gene List

Automatically Derived For You (Glad4U) [181]. Rows are diseases, columns are ID number and name of the disease (ontology, name), total number of genes associated with that disease in the Glad4U database (refnum), total number of DMR genes associated with the disease (interestnum), hypergeometric and BY-corrected p-values computed with WebGestalt (pvalue, adjustp), and DMR gene ids (genes), same as geneSym column in Additional file 2: Table S1B.

Table S10: BS-Seq information

Table shows each fish in the study (rows), columns are social status of the fish (dominant or nondominant; Group), tissue id number (Sample), sequencing id number (Sequencing), total number of BS-Seq read pairs sequenced (Total), number of read pairs that passed quality control (Post-QC filter), number of read pairs that aligned to the *A.burtoni* genome before and after trimming the first and last three bases (Aligned pre-, post-trimmed; see Additional file 1: Figure S15), percentage of quality-controlled read pairs that successfully aligned (Percent aligned), approximate genome coverage of alignments (Final genomic coverage), total number of cytosines that were sequenced (Valid mappings) and had at least 4x coverage in every fish (Covered), approximate cytosine coverage across the genome (Average Coverage).

Figure S1

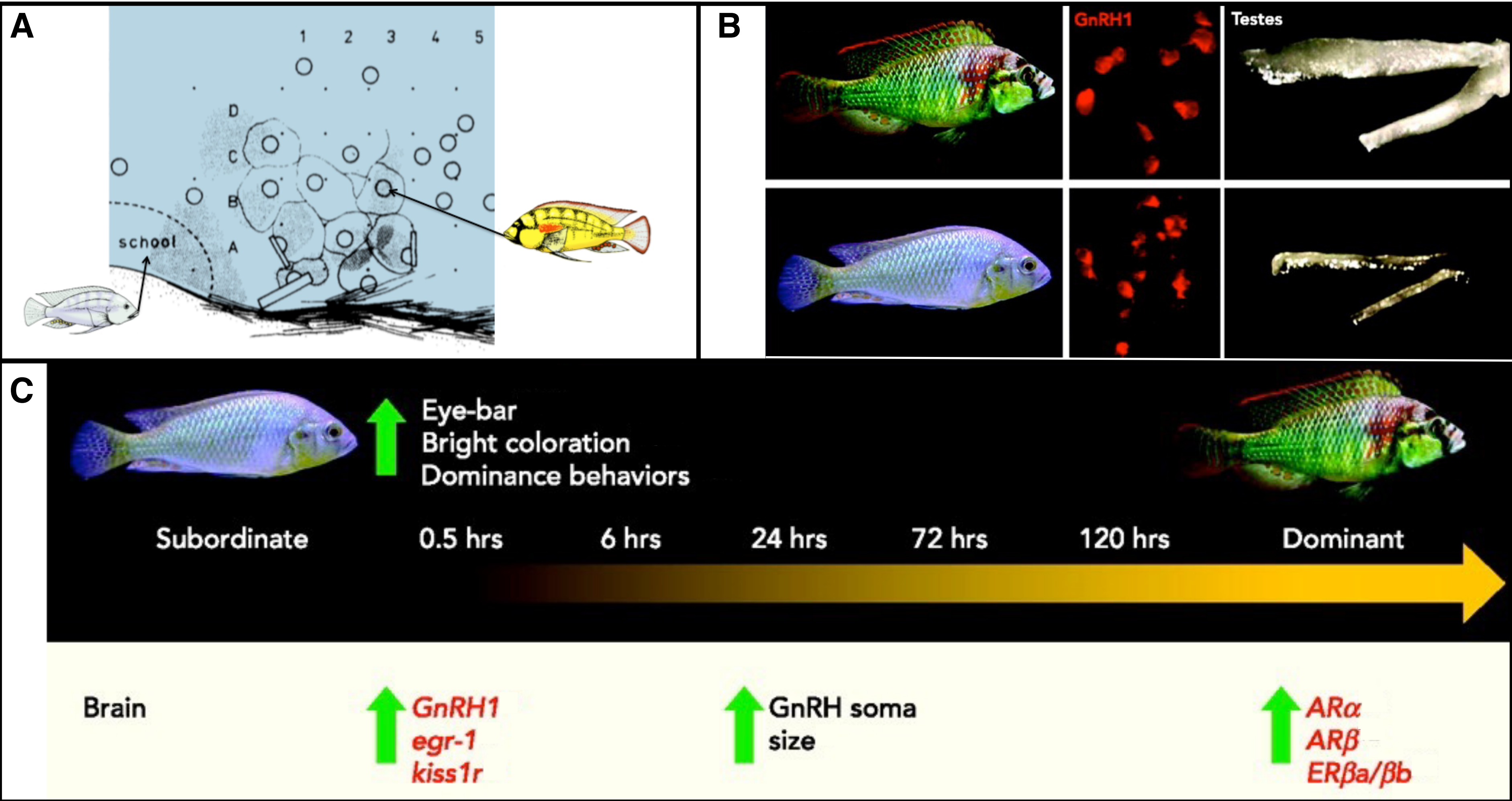
A
1 2 3 4 5
D
C
B
A
school
B
GnRH1
Testes
C
Eye-bar
Bright coloration
Dominance behaviors
Subordinate
0.5 hrs
6 hrs
24 hrs
72 hrs
120 hrs
Dominant
Brain
GnRH1
egr-1
kiss1r
GnRH soma size
ARα
ARβ
ERβa/βb

Figure S2

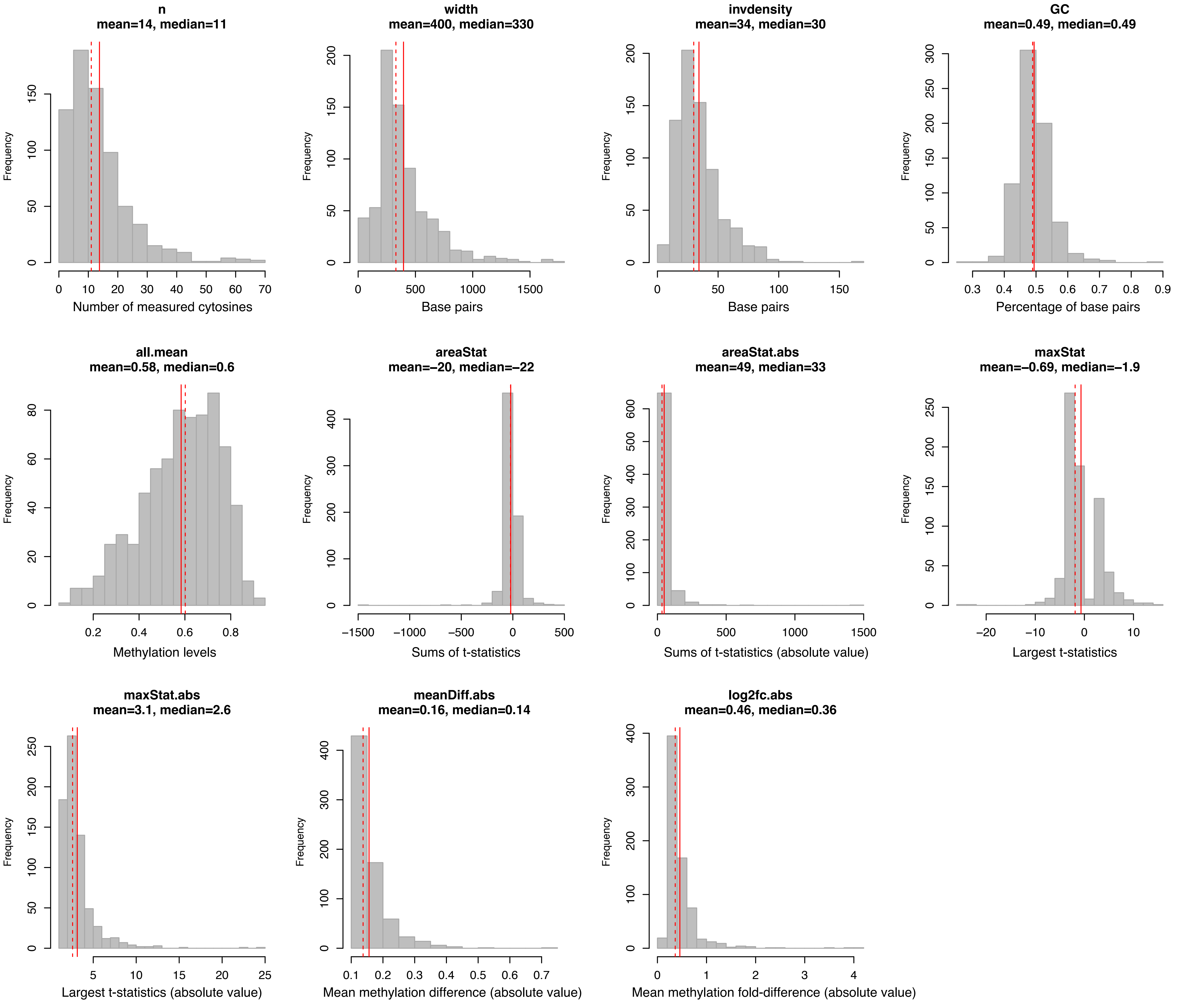

n
mean=14, median=11
Frequency
Number of measured cytosines
width
mean=400, median=330
Frequency
Base pairs
invdensity
mean=34, median=30
Frequency
Base pairs
GC
mean=0.49, median=0.49
Frequency
Percentage of base pairs
all.mean
mean=0.58, median=0.6
Frequency
Methylation levels
areaStat
mean=−20, median=−22
Frequency
Sums of t-statistics
areaStat.abs
mean=49, median=33
Frequency
Sums of t-statistics (absolute value)
maxStat
mean=−0.69, median=−1.9
Frequency
Largest t-statistics
maxStat.abs
mean=3.1, median=2.6
Frequency
Largest t-statistics (absolute value)
meanDiff.abs
mean=0.16, median=0.14
Frequency
Mean methylation difference (absolute value)
log2fc.abs
mean=0.46, median=0.36
Frequency
Mean methylation fold-difference (absolute value)

Figure S3

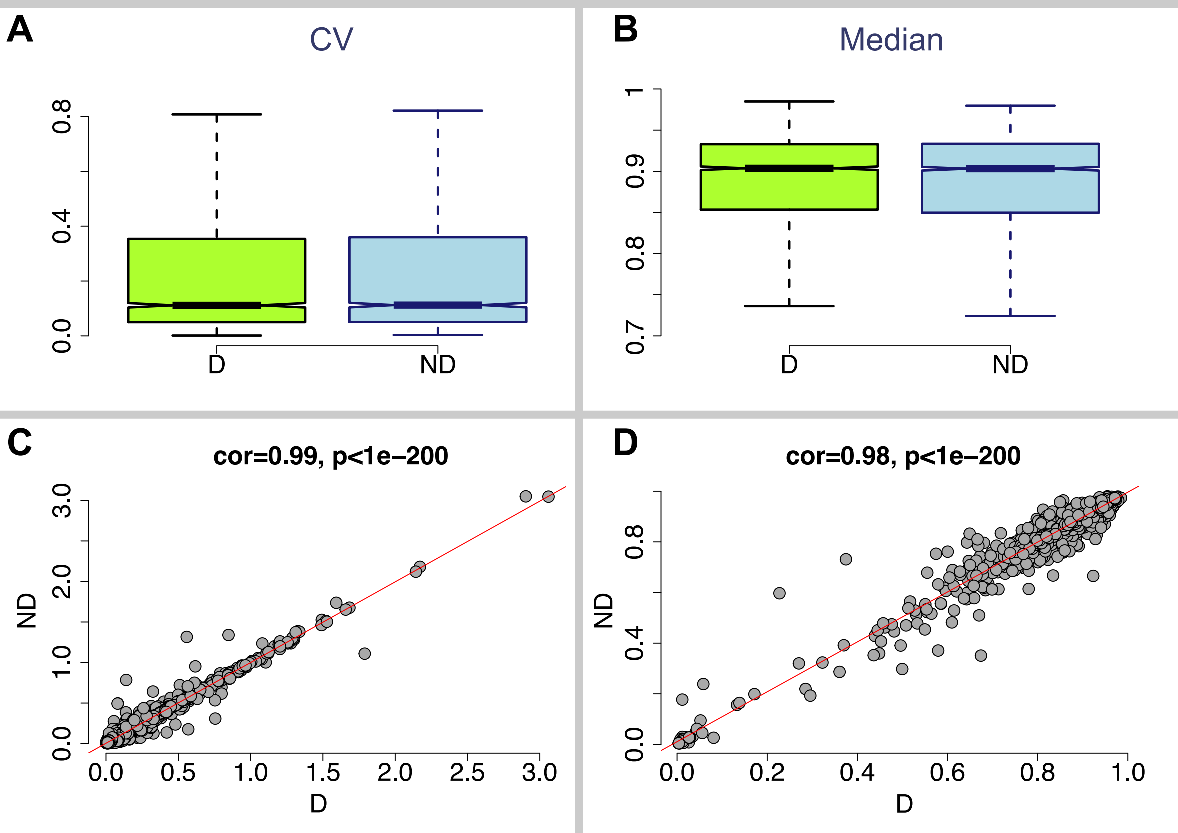
A
CV
0.0
0.4
0.8
D
ND
B
Median
0.7
0.8
0.9
1
D
ND
C
cor=0.99, p<1e−200
0.0
1.0
2.0
3.0
ND
0.0
0.5
1.0
1.5
2.0
2.5
3.0
D
D
cor=0.98, p<1e−200
0.0
0.4
0.8
ND
0.0
0.2
0.4
0.6
0.8
1.0
D

Figure S4

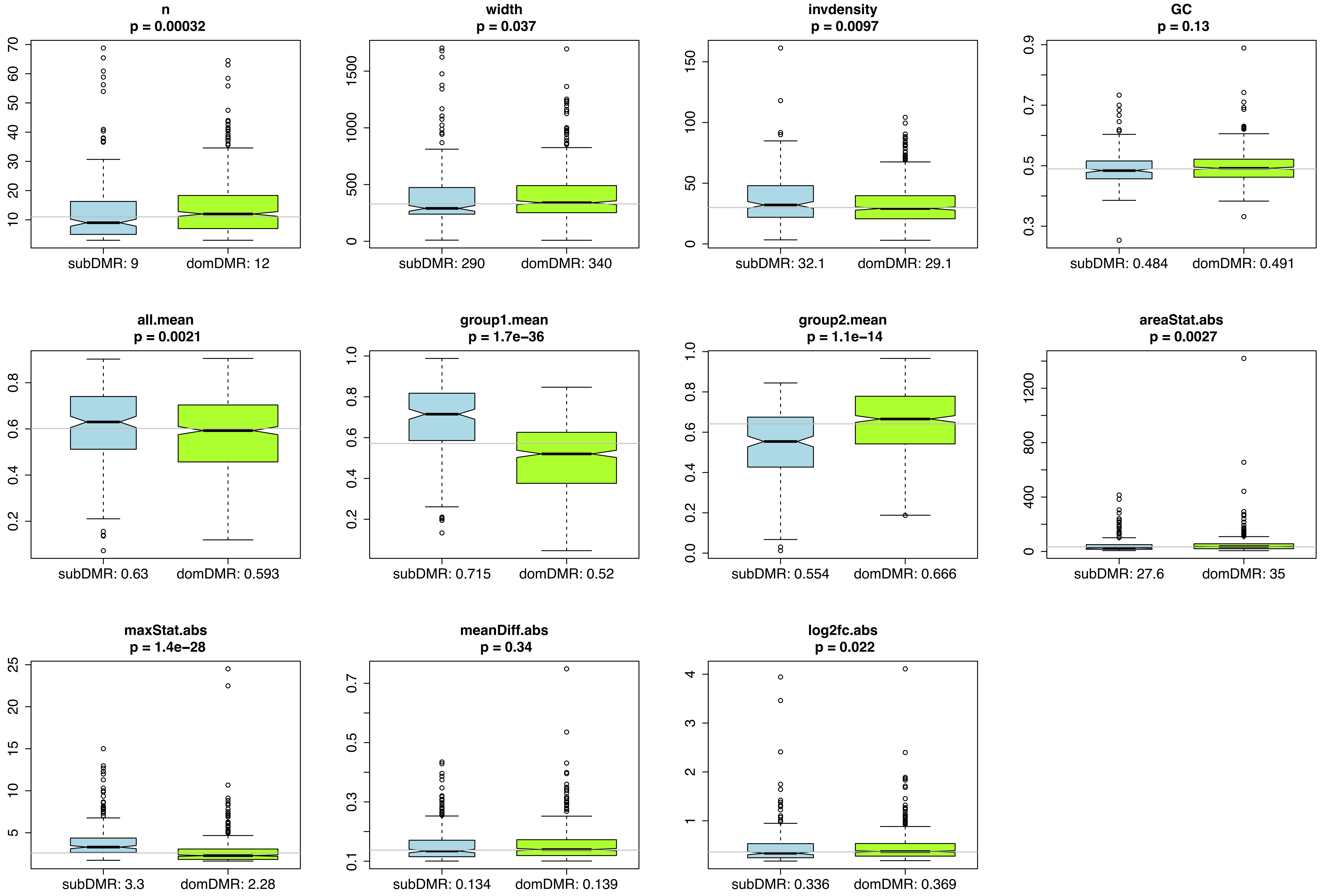
n
p = 0.00032
subDMR: 9
domDMR: 12
width
p = 0.037
subDMR: 290
domDMR: 340
invdensity
p = 0.0097
subDMR: 32.1
domDMR: 29.1
GC
p = 0.13
subDMR: 0.484
domDMR: 0.491
all.mean
p = 0.0021
subDMR: 0.63
domDMR: 0.593
group1.mean
p = 1.7e−36
subDMR: 0.715
domDMR: 0.52
group2.mean
p = 1.1e−14
subDMR: 0.554
domDMR: 0.666
areaStat.abs
p = 0.0027
subDMR: 27.6
domDMR: 35
maxStat.abs
p = 1.4e−28
subDMR: 3.3
domDMR: 2.28
meanDiff.abs
p = 0.34
subDMR: 0.134
domDMR: 0.139
log2fc.abs
p = 0.022
subDMR: 0.336
domDMR: 0.369

Figure S5

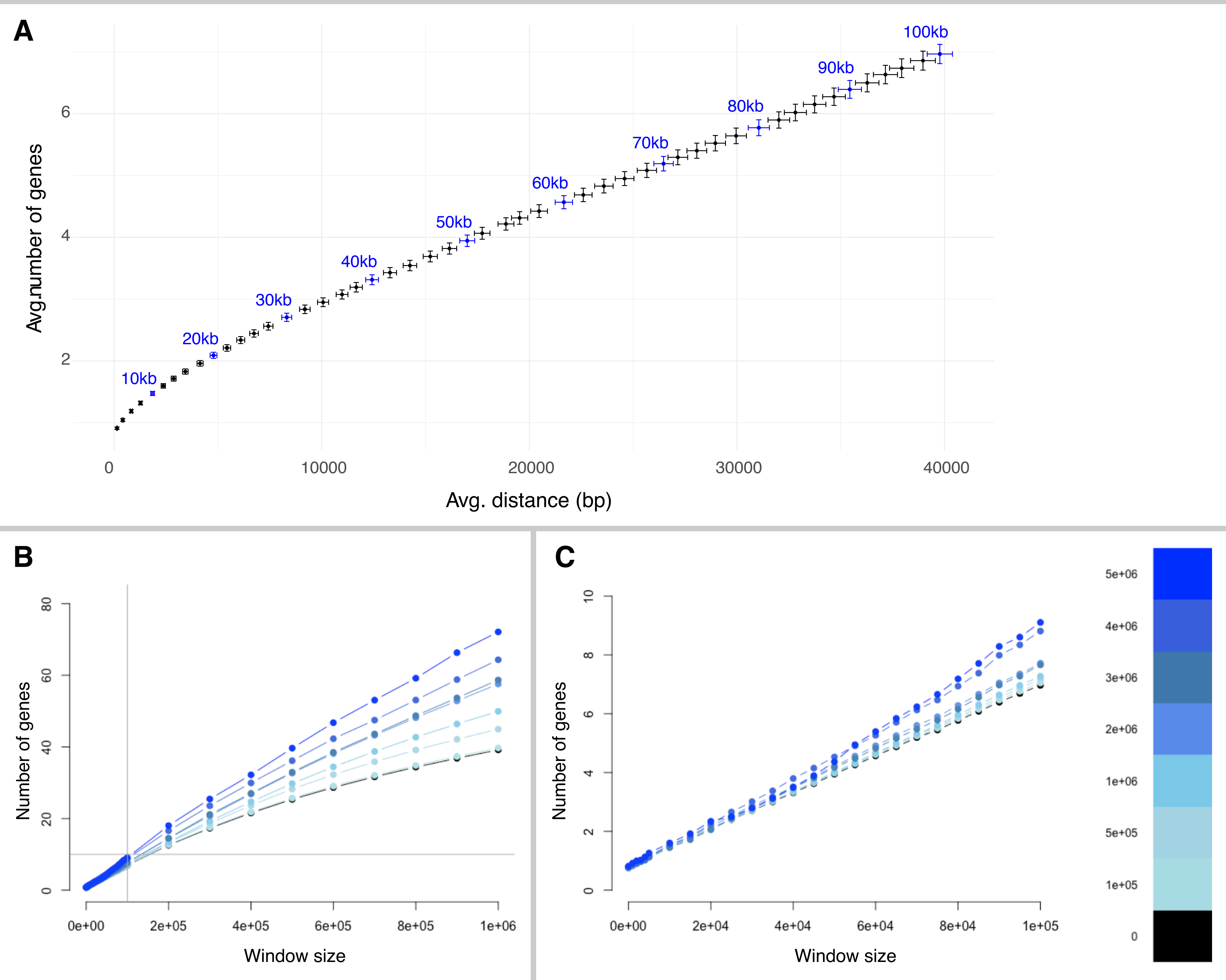
A
Avg.number of genes
Avg. distance (bp)
10kb
20kb
30kb
40kb
50kb
60kb
70kb
80kb
90kb
100kb
0
10000
20000
30000
40000
2
4
6
B
Number of genes
Window size
0e+00
2e+05
4e+05
6e+05
8e+05
1e+06
0
20
40
60
80
C
Number of genes
Window size
0e+00
2e+04
4e+04
6e+04
8e+04
1e+05
0
2
4
6
8
10
5e+06
4e+06
3e+06
2e+06
1e+06
5e+05
1e+05
0

Figure S6

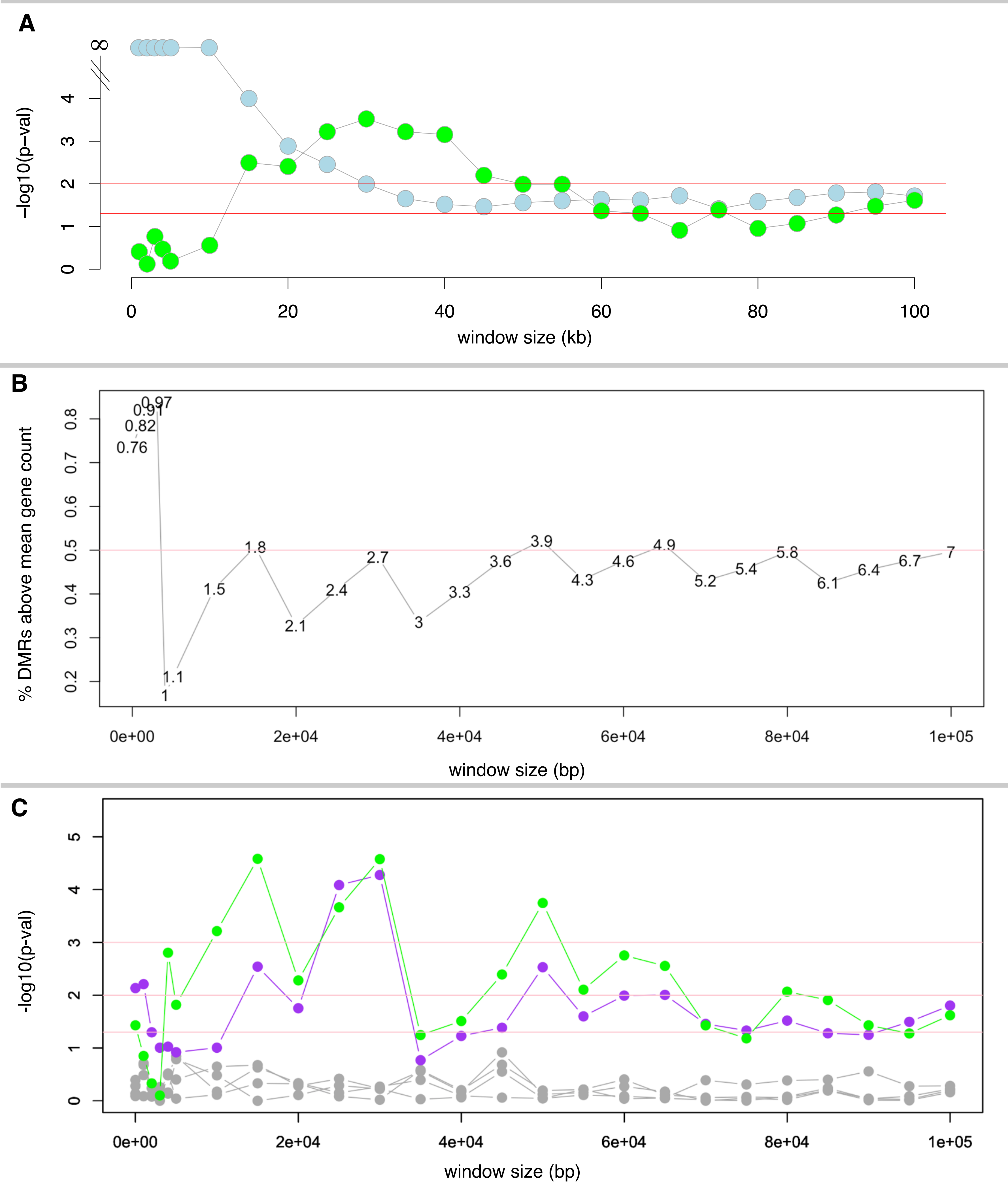
A
−log10(p−val)
∞
0
1
2
3
4
0
20
40
60
80
100
window size (kb)
B
% DMRs above mean gene count
0.2
0.3
0.4
0.5
0.6
0.7
0.8
0.97
0.91
0.82
0.76
1.1
1
1.5
1.8
2.1
2.4
2.7
3
3.3
3.6
3.9
4.3
4.6
4.9
5.2
5.4
5.8
6.1
6.4
6.7
7
0e+00
2e+04
4e+04
6e+04
8e+04
1e+05
window size (bp)
C
-log10(p-val)
0
1
2
3
4
5
0e+00
2e+04
4e+04
6e+04
8e+04
1e+05
window size (bp)

Figure S7

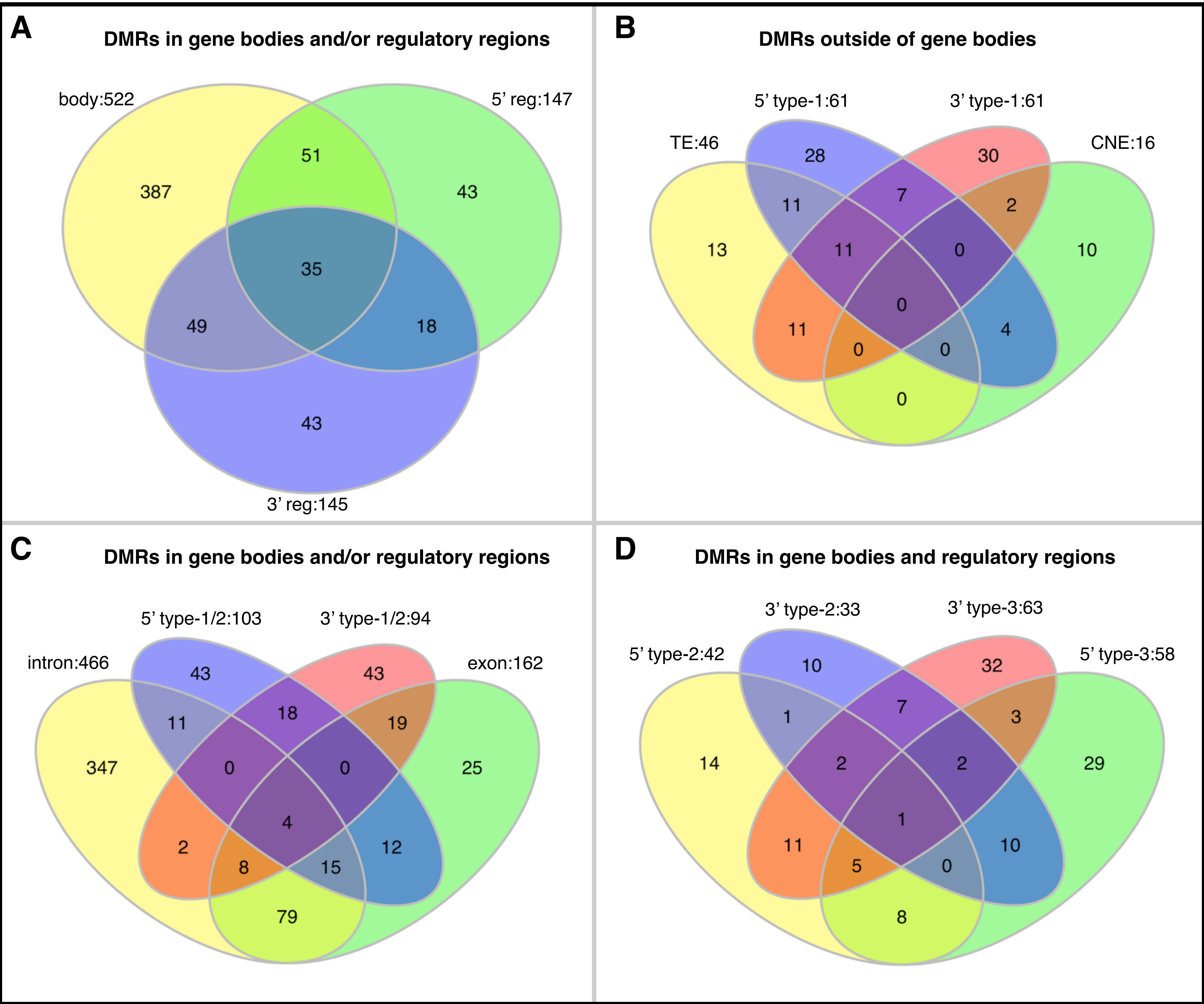
A
DMRs in gene bodies and/or regulatory regions
body:522
5’ reg:147
3’ reg:145
387
51
43
35
49
18
43
B
DMRs outside of gene bodies
TE:46
5’ type-1:61
3’ type-1:61
CNE:16
28
30
11
7
2
13
11
0
10
0
11
0
0
4
0
C
DMRs in gene bodies and/or regulatory regions
intron:466
5’ type-1/2:103
3’ type-1/2:94
exon:162
43
43
11
18
19
347
0
0
25
4
2
8
15
12
79
D
DMRs in gene bodies and regulatory regions
5’ type-2:42
3’ type-2:33
3’ type-3:63
5’ type-3:58
10
32
1
7
3
14
2
2
29
1
11
5
0
10
8

Figure S8

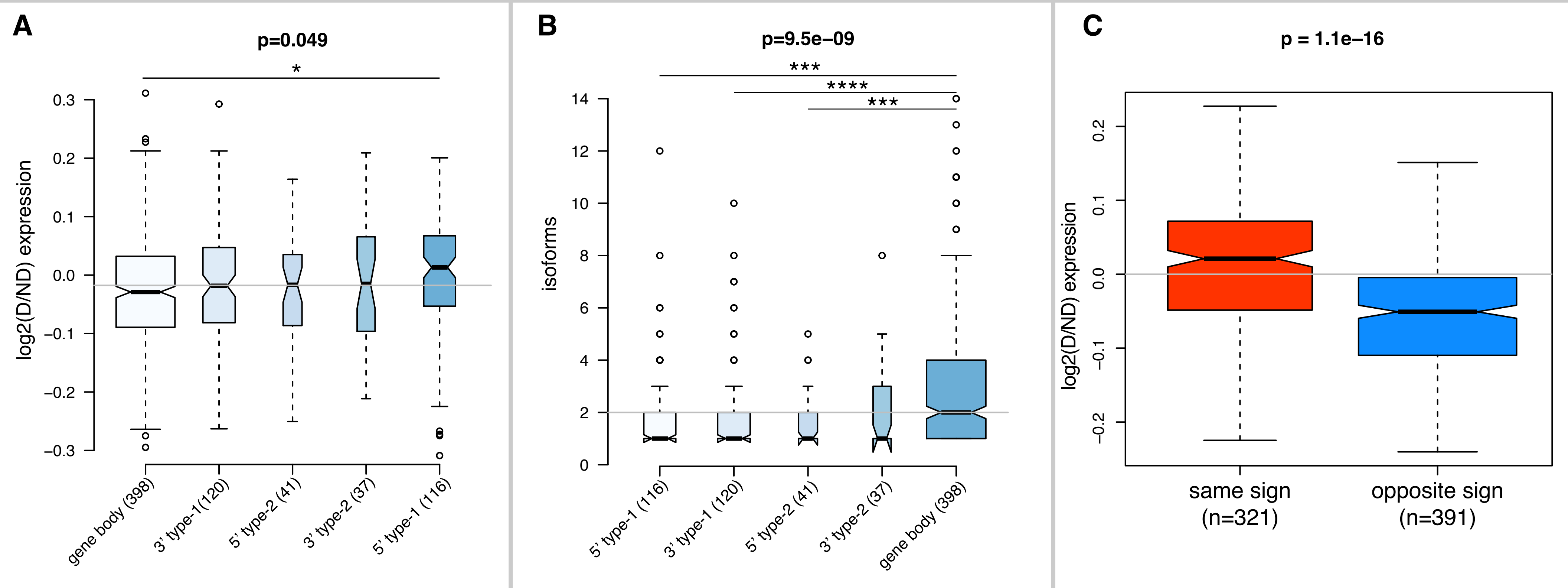

A
p=0.049
*
log2(D/ND) expression
0.3
0.2
0.1
0.0
−0.1
−0.2
−0.3
gene body (398)
3' type-1(120)
5' type-2 (41)
3' type-2 (37)
5' type-1 (116)
B
p=9.5e−09
***
****
***
isoforms
14
12
10
8
6
4
2
0
5' type-1 (116)
3' type-1 (120)
5' type-2 (41)
3' type-2 (37)
gene body (398)
C
p = 1.1e−16
log2(D/ND) expression
0.2
0.1
0.0
−0.1
−0.2
same sign
(n=321)
opposite sign
(n=391)

Figure S9

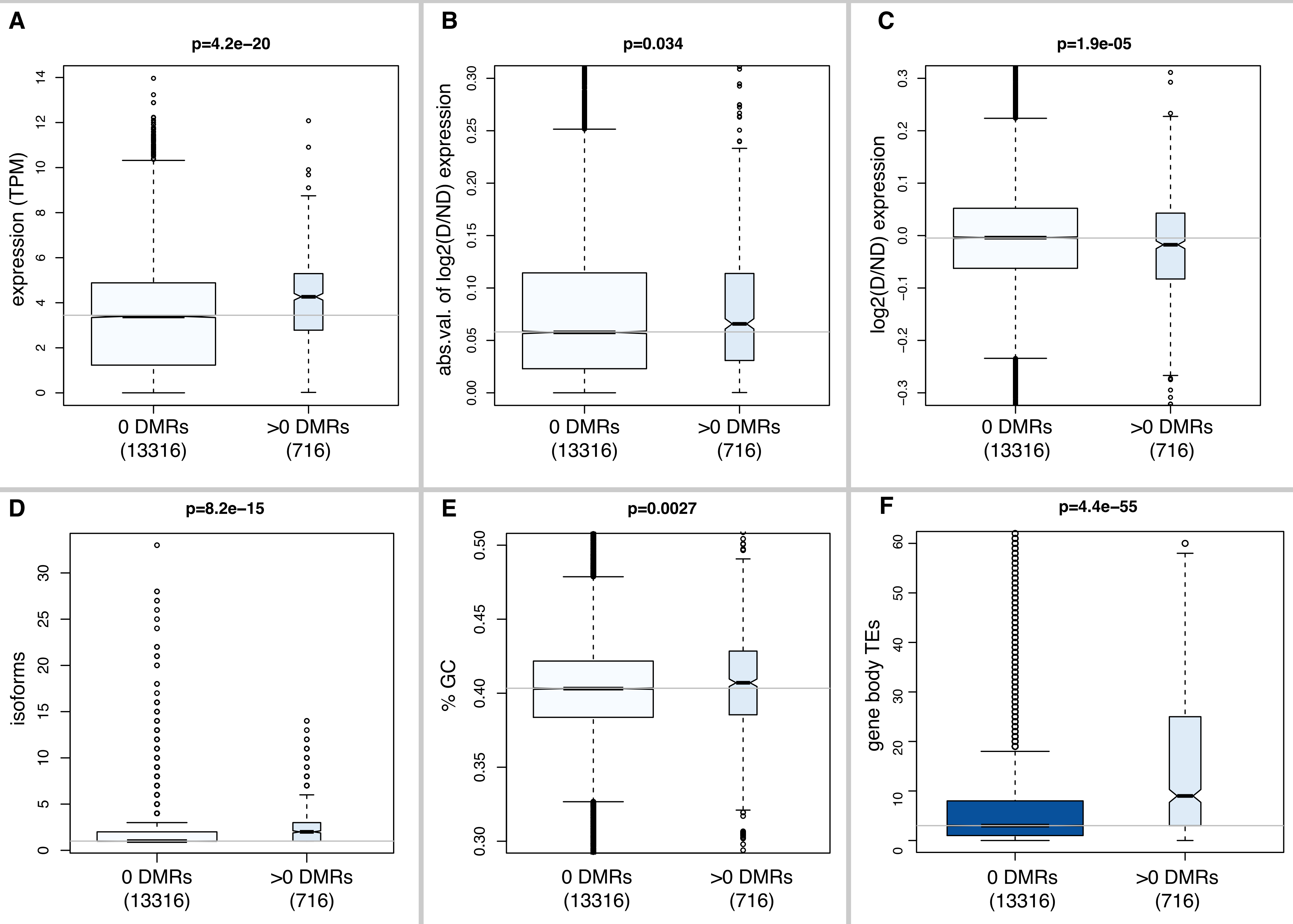
A
p=4.2e−20
expression (TPM)
0 DMRs (13316)
>0 DMRs (716)
B
p=0.034
abs.val. of log2(D/ND) expression
C
p=1.9e-05
log2(D/ND) expression
D
p=8.2e−15
isoforms
E
p=0.0027
% GC
F
p=4.4e−55
gene body TEs

Figure S10

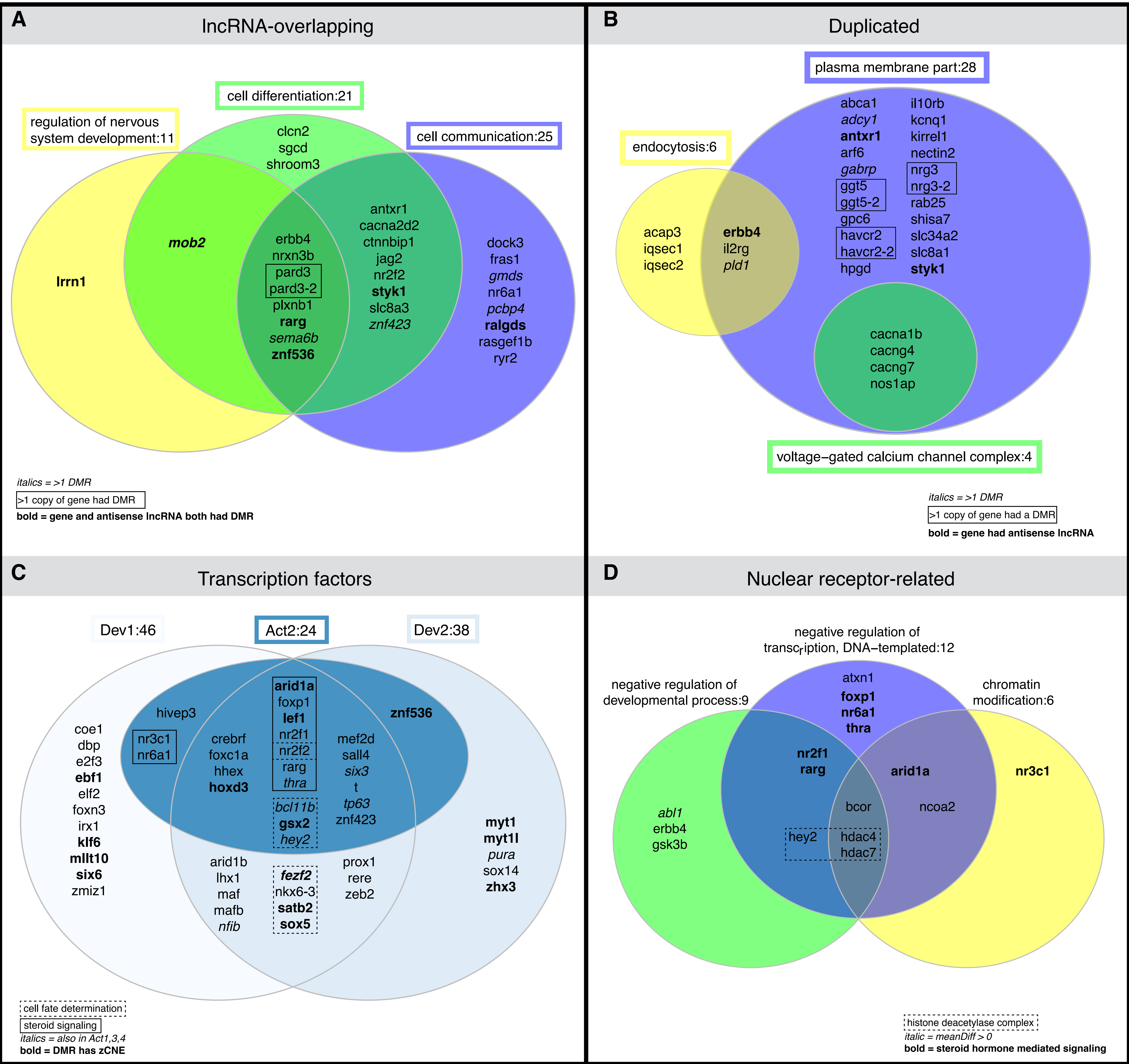
A
lncRNA-overlapping
regulation of nervous system development:11
cell differentiation:21
cell communication:25
clcn2
sgcd
shroom3
mob2
lrrn1
erbb4
nrxn3b
pard3
pard3-2
plxnb1
rarg
sema6b
znf536
antxr1
cacna2d2
ctnnbip1
jag2
nr2f2
styk1
slc8a3
znf423
dock3
fras1
gmds
nr6a1
pcbp4
ralgds
rasgef1b
ryr2
italics = >1 DMR
>1 copy of gene had DMR
bold = gene and antisense lncRNA both had DMR
B
Duplicated
plasma membrane part:28
endocytosis:6
abca1
adcy1
antxr1
arf6
gabrp
ggt5
ggt5-2
gpc6
havcr2
havcr2-2
hpgd
il10rb
kcnq1
kirrel1
nectin2
nrg3
nrg3-2
rab25
shisa7
slc34a2
slc8a1
styk1
acap3
iqsec1
iqsec2
erbb4
il2rg
pld1
cacna1b
cacng4
cacng7
nos1ap
voltage–gated calcium channel complex:4
italics = >1 DMR
>1 copy of gene had a DMR
bold = gene had antisense lncRNA
C
Transcription factors
Dev1:46
Act2:24
Dev2:38
hivep3
nr3c1
nr6a1
coe1
dbp
e2f3
ebf1
elf2
foxn3
irx1
klf6
mllt10
six6
zmiz1
crebrf
foxc1a
hhex
hoxd3
arid1a
foxp1
lef1
nr2f1
nr2f2
rarg
thra
bcl11b
gsx2
hey2
mef2d
sall4
six3
t
tp63
znf423
znf536
arid1b
lhx1
maf
mafb
nfib
fezf2
nkx6-3
satb2
sox5
prox1
rere
zeb2
myt1
myt1l
pura
sox14
zhx3
cell fate determination
steroid signaling
italics = also in Act1,3,4
bold = DMR has zCNE
D
Nuclear receptor-related
negative regulation of transcription, DNA–templated:12
negative regulation of developmental process:9
chromatin modification:6
atxn1
foxp1
nr6a1
thra
nr2f1
rarg
arid1a
nr3c1
abl1
erbb4
gsk3b
bcor
ncoa2
hey2
hdac4
hdac7
histone deacetylase complex
italic = meanDiff > 0
bold = steroid hormone mediated signaling

Figure S11

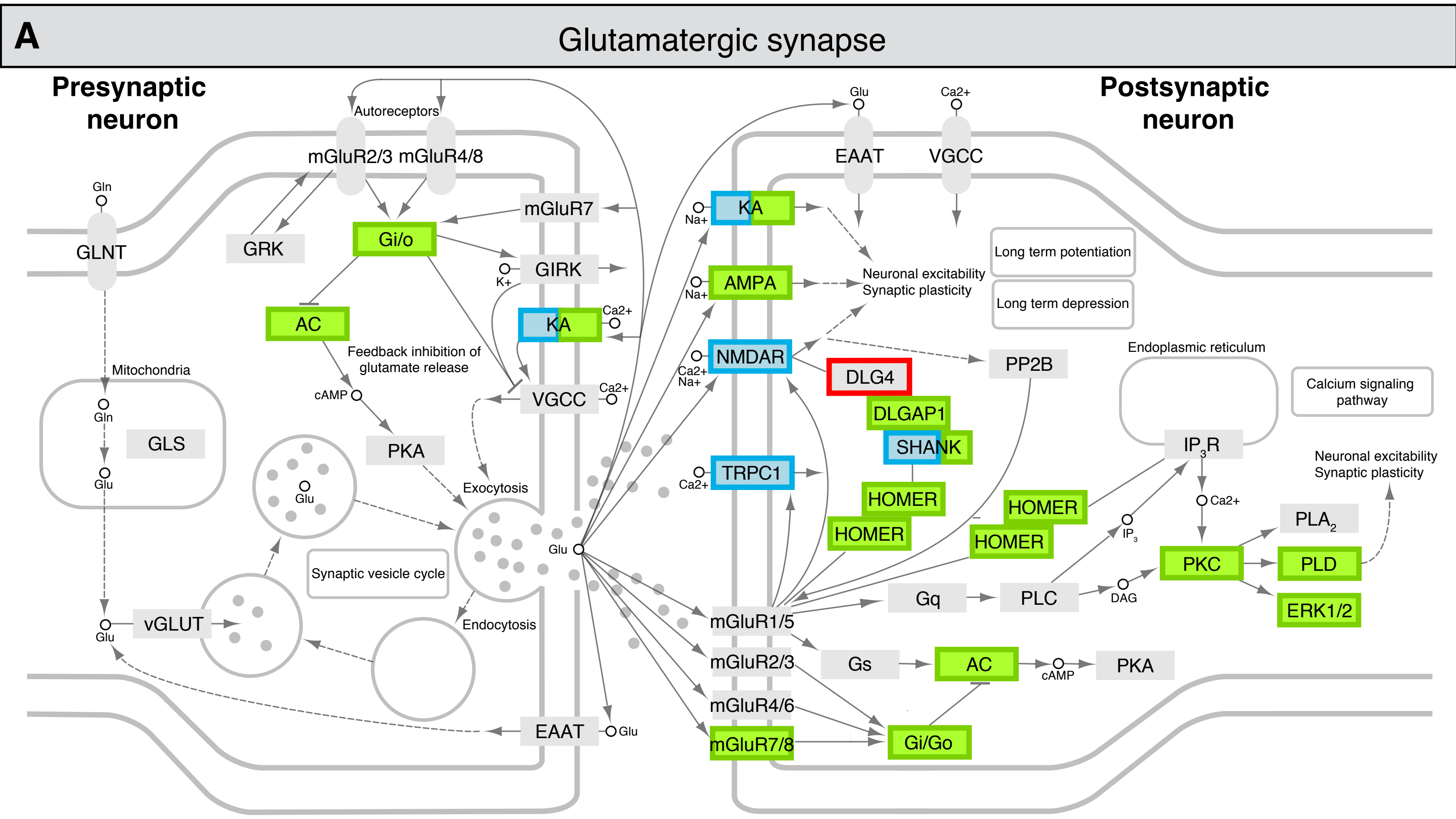
A
Glutamatergic synapse
Presynaptic neuron
Postsynaptic neuron
Autoreceptors
mGluR2/3 mGluR4/8
GRK
Gi/o
mGluR7
GIRK
KA
AC
Feedback inhibition of glutamate release
cAMP
PKA
VGCC
GLNT
Gln
Mitochondria
GLS
Glu
Exocytosis
Endocytosis
Synaptic vesicle cycle
vGLUT
EAAT
Na+
K+
Ca2+
AMPA
NMDAR
TRPC1
Neuronal excitability
Synaptic plasticity
Long term potentiation
Long term depression
PP2B
DLG4
DLGAP1
SHANK
HOMER
Endoplasmic reticulum
Calcium signaling pathway
IP_3R
IP_3
PLA_2
PKC
PLD
ERK1/2
Gq
PLC
DAG
mGluR1/5
mGluR2/3
mGluR4/6
mGluR7/8
Gs
Gi/Go
PKA

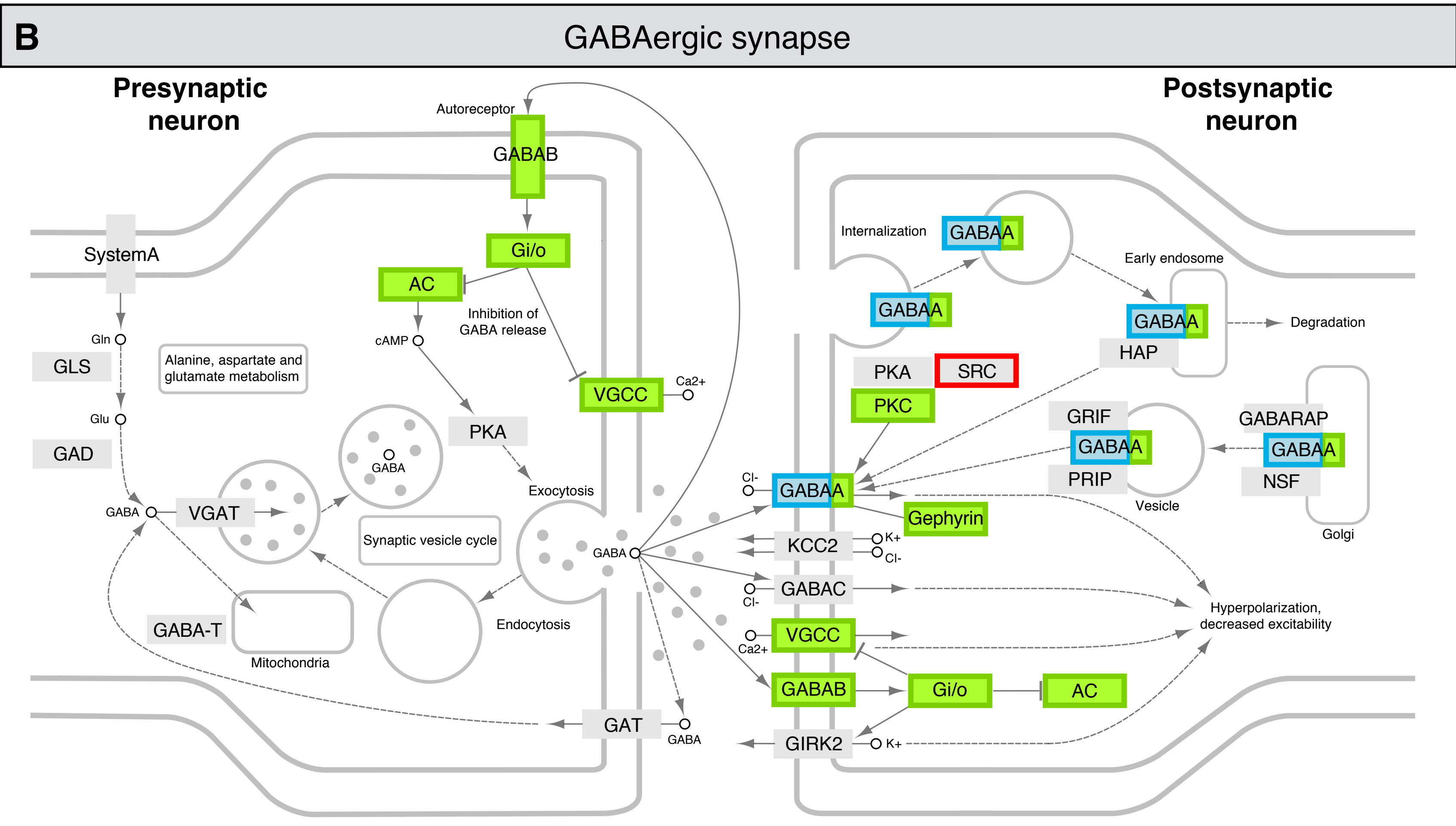
B
GABAergic synapse
Presynaptic neuron
Postsynaptic neuron
Autoreceptor
GABAB
Gi/o
AC
Inhibition of GABA release
cAMP
PKA
VGCC
Ca2+
SystemA
Gln
GLS
Glu
GAD
Alanine, aspartate and glutamate metabolism
GABA
VGAT
Synaptic vesicle cycle
Exocytosis
Endocytosis
GABA-T
Mitochondria
GAT
Internalization
GABAA
Early endosome
Degradation
HAP
PKA
SRC
PKC
GRIF
PRIP
Vesicle
GABARAP
NSF
Golgi
Cl-
Gephyrin
KCC2
K+
GABAC
Hyperpolarization, decreased excitability
GIRK2

Figure S12

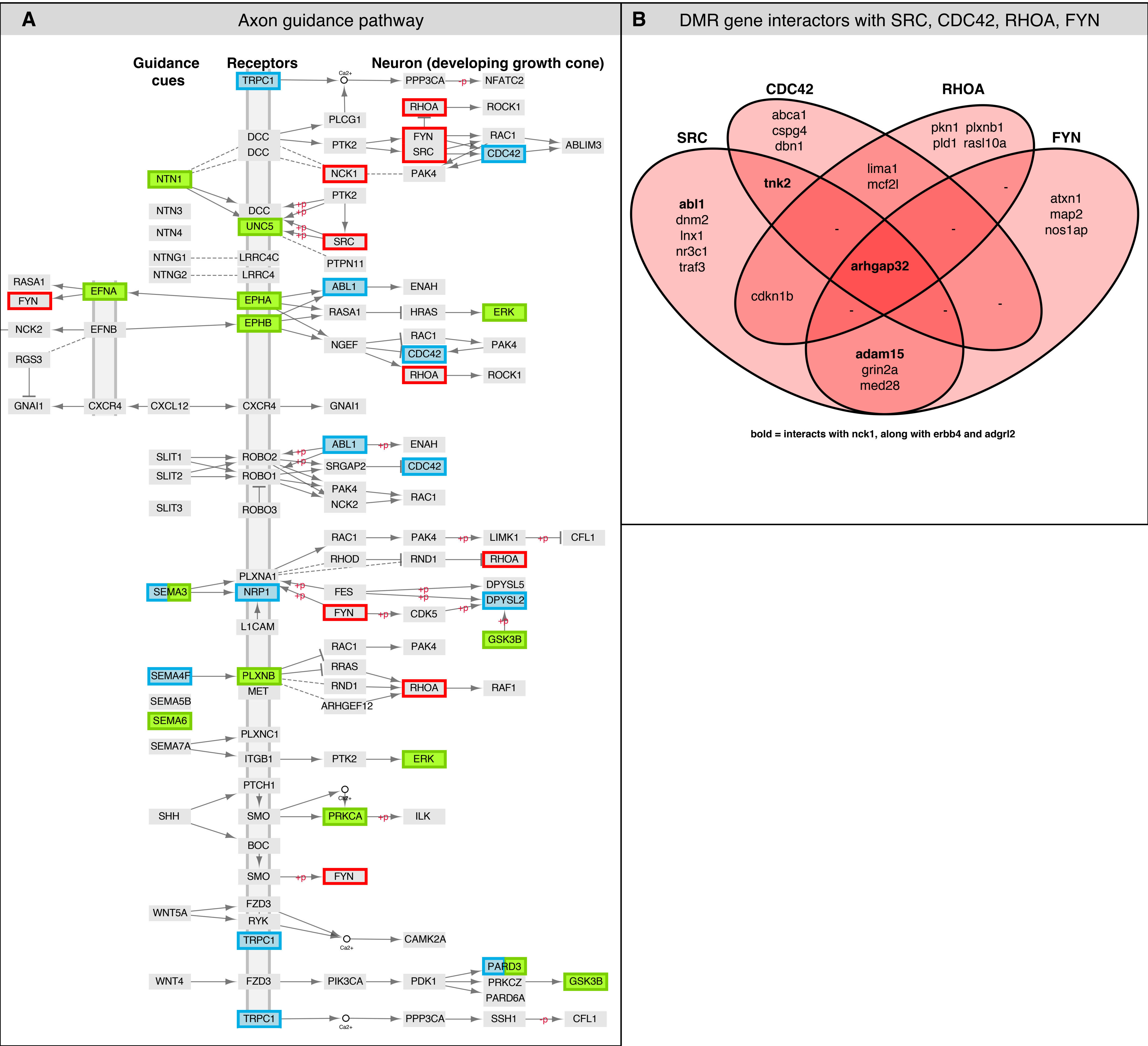
A
Axon guidance pathway
Guidance cues
Receptors
Neuron (developing growth cone)
TRPC1
Ca2+
PPP3CA
NFATC2
RHOA
ROCK1
PLCG1
DCC
PTK2
FYN
SRC
RAC1
CDC42
ABLIM3
NTN1
NCK1
PAK4
NTN3
NTN4
UNC5
NTNG1
NTNG2
LRRC4C
LRRC4
PTPN11
RASA1
EFNA
EPHA
ABL1
ENAH
HRAS
ERK
NCK2
EFNB
EPHB
NGEF
RGS3
GNAI1
CXCR4
CXCL12
SLIT1
SLIT2
SLIT3
ROBO2
ROBO1
ROBO3
SRGAP2
LIMK1
CFL1
RHOD
RND1
PLXNA1
SEMA3
NRP1
FES
DPYSL5
DPYSL2
CDK5
GSK3B
L1CAM
SEMA4F
PLXNB
MET
RRAS
ARHGEF12
RAF1
SEMA5B
SEMA6
SEMA7A
PLXNC1
ITGB1
PTCH1
SHH
SMO
PRKCA
ILK
BOC
WNT5A
FZD3
RYK
CAMK2A
PARD3
WNT4
PIK3CA
PDK1
PRKCZ
PARD6A
SSH1
+p
-p
B
DMR gene interactors with SRC, CDC42, RHOA, FYN
SRC
CDC42
RHOA
FYN
abca1
cspg4
dbn1
pkn1 plxnb1
pld1 rasl10a
lima1
mcf2l
tnk2
abl1
dnm2
lnx1
nr3c1
traf3
atxn1
map2
nos1ap
arhgap32
cdkn1b
adam15
grin2a
med28
-
bold = interacts with nck1, along with erbb4 and adgrl2

Figure S13

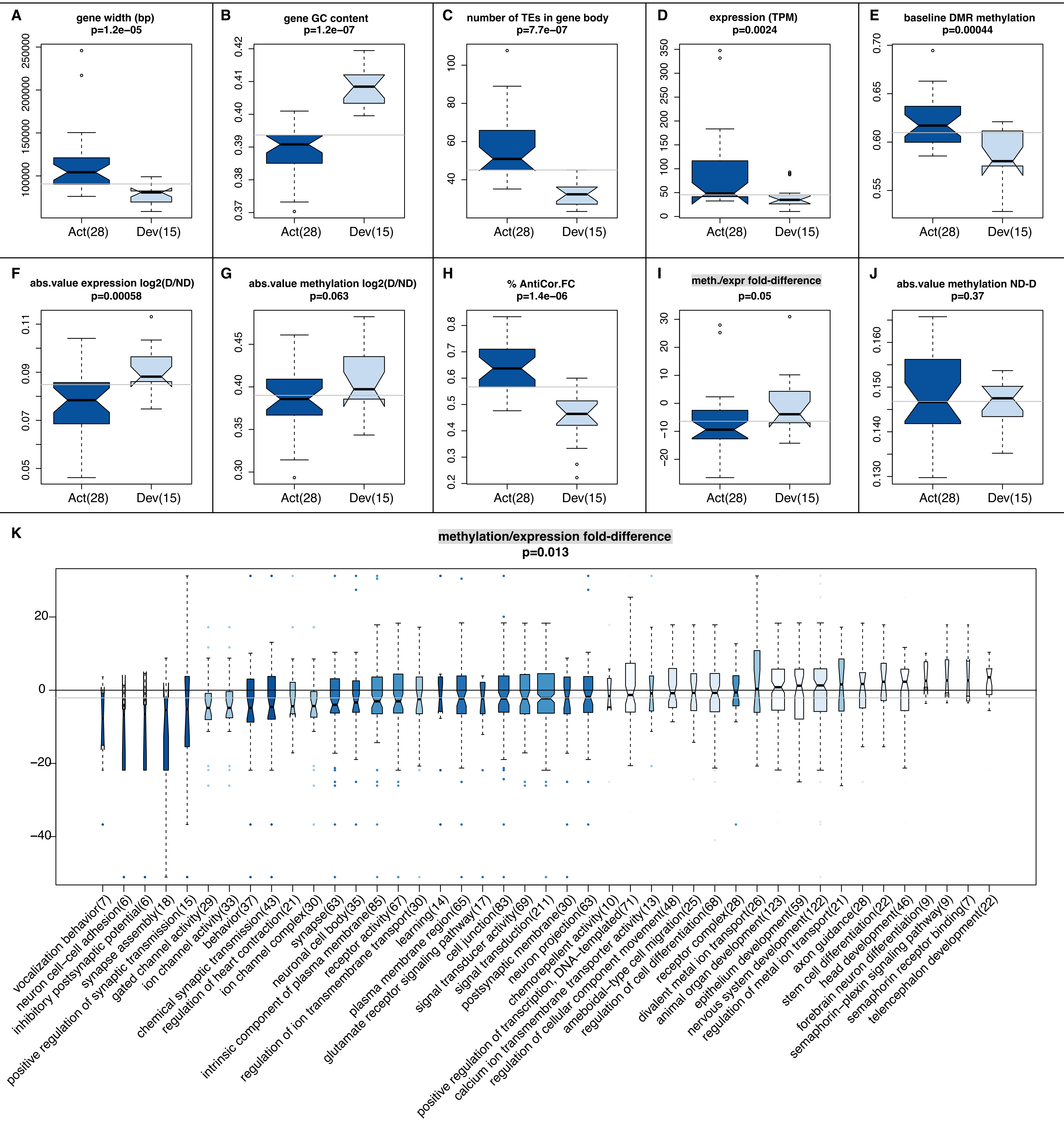
A
gene width (bp)
p=1.2e−05
Act(28)
Dev(15)
B
gene GC content
p=1.2e−07
C
number of TEs in gene body
p=7.7e−07
D
expression (TPM)
p=0.0024
E
baseline DMR methylation
p=0.00044
F
abs.value expression log2(D/ND)
p=0.00058
G
abs.value methylation log2(D/ND)
p=0.063
H
% AntiCor.FC
p=1.4e−06
I
meth./expr fold-difference
p=0.05
J
abs.value methylation ND-D
p=0.37
K
methylation/expression fold-difference
p=0.013
vocalization behavior(7)
neuron cell−cell adhesion(6)
inhibitory postsynaptic potential(6)
synapse assembly(18)
positive regulation of synaptic transmission(15)
gated channel activity(29)
ion channel activity(33)
behavior(37)
chemical synaptic transmission(43)
regulation of heart contraction(21)
ion channel complex(30)
synapse(63)
neuronal cell body(35)
intrinsic component of plasma membrane(85)
receptor activity(67)
regulation of ion transmembrane transport(30)
learning(14)
plasma membrane region(65)
glutamate receptor signaling pathway(17)
cell junction(83)
signal transducer activity(69)
signal transduction(211)
postsynaptic membrane(30)
neuron projection(63)
chemorepellent activity(10)
positive regulation of transcription, DNA−templated(71)
calcium ion transmembrane transporter activity(13)
regulation of cellular component movement(48)
ameboidal−type cell migration(25)
regulation of cell differentiation(68)
receptor complex(28)
divalent metal ion transport(26)
animal organ development(123)
epithelium development(59)
nervous system development(122)
regulation of metal ion transport(21)
axon guidance(28)
stem cell differentiation(22)
head development(46)
forebrain neuron differentiation(9)
semaphorin−plexin signaling pathway(9)
semaphorin receptor binding(7)
telencephalon development(22)

Figure S14

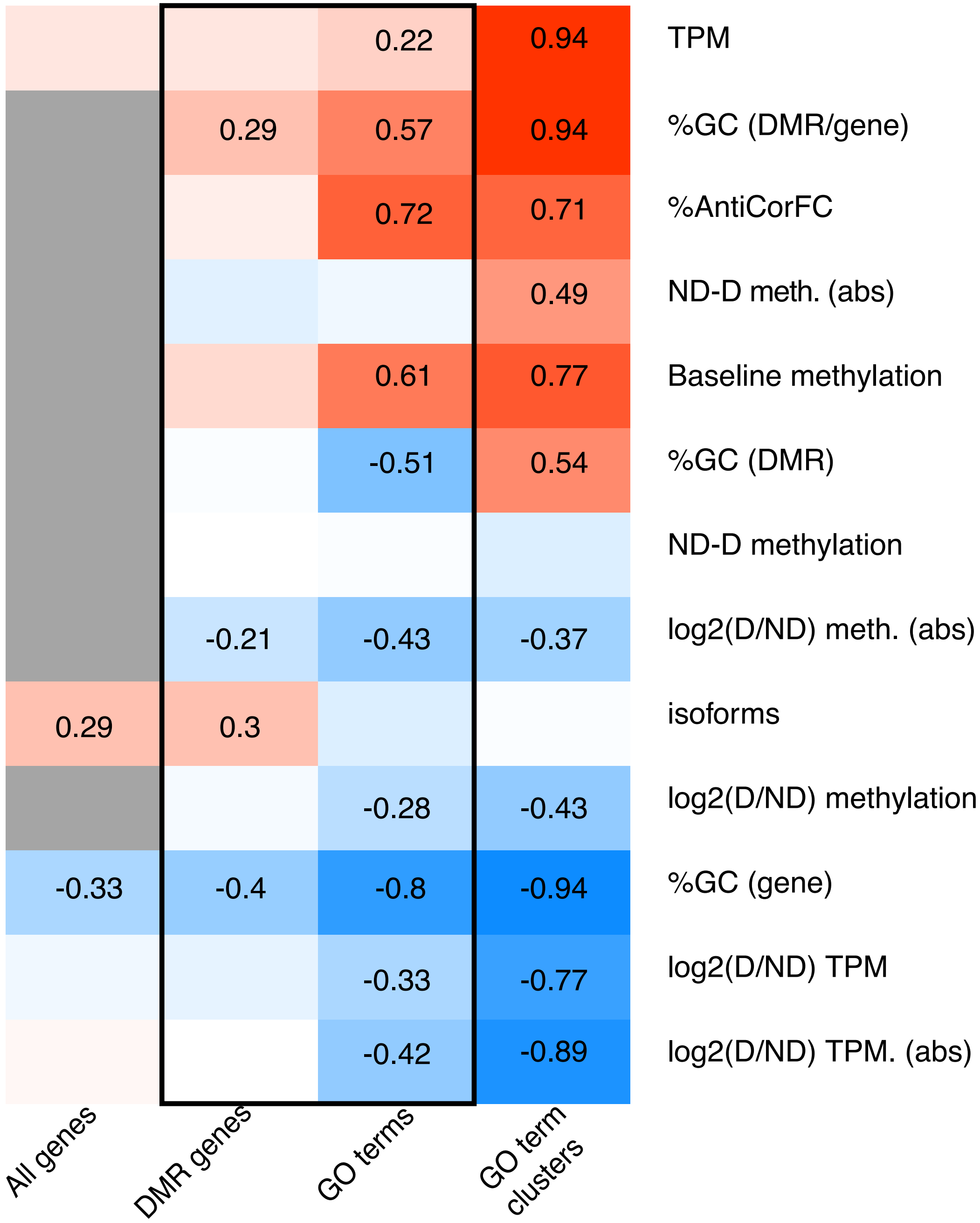
0.22
0.94
TPM
0.29
0.57
0.94
%GC (DMR/gene)
0.72
0.71
%AntiCorFC
0.49
ND-D meth. (abs)
0.61
0.77
Baseline methylation
-0.51
0.54
%GC (DMR)
ND-D methylation
-0.21
-0.43
-0.37
log2(D/ND) meth. (abs)
0.29
0.3
isoforms
-0.28
-0.43
log2(D/ND) methylation
-0.33
-0.4
-0.8
-0.94
%GC (gene)
-0.33
-0.77
log2(D/ND) TPM
-0.42
-0.89
log2(D/ND) TPM. (abs)
All genes
DMR genes
GO terms
GO term clusters

Figure S15

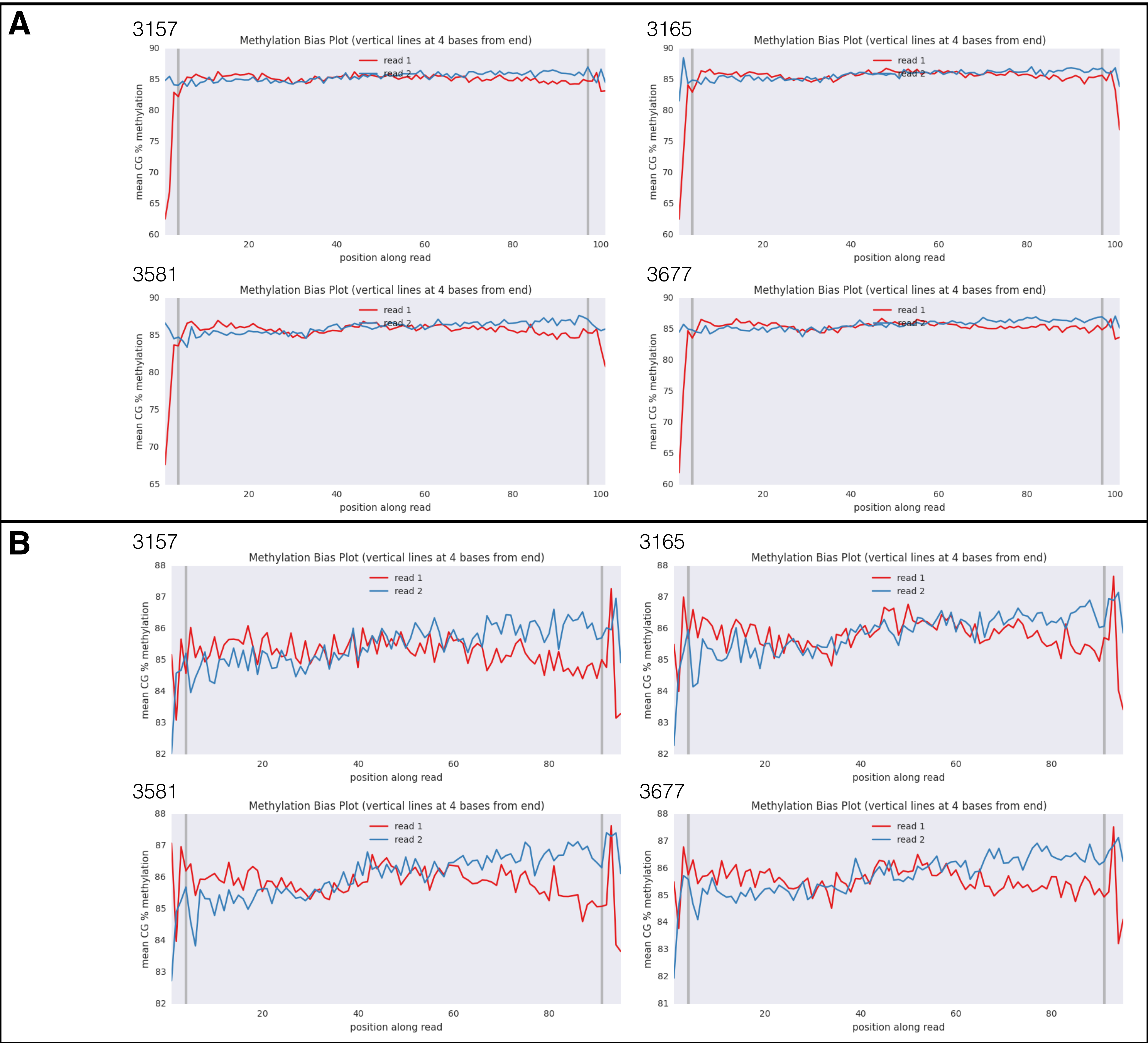
A
3157
Methylation Bias Plot (vertical lines at 4 bases from end)
read 1
read 2
mean CG % methylation
position along read
3165
Methylation Bias Plot (vertical lines at 4 bases from end)
read 1
read 2
mean CG % methylation
position along read
3581
Methylation Bias Plot (vertical lines at 4 bases from end)
read 1
read 2
mean CG % methylation
position along read
3677
Methylation Bias Plot (vertical lines at 4 bases from end)
read 1
read 2
mean CG % methylation
position along read
B
3157
Methylation Bias Plot (vertical lines at 4 bases from end)
read 1
read 2
mean CG % methylation
position along read
3165
Methylation Bias Plot (vertical lines at 4 bases from end)
read 1
read 2
mean CG % methylation
position along read
3581
Methylation Bias Plot (vertical lines at 4 bases from end)
read 1
read 2
mean CG % methylation
position along read
3677
Methylation Bias Plot (vertical lines at 4 bases from end)
read 1
read 2
mean CG % methylation
position along read

Figure S16

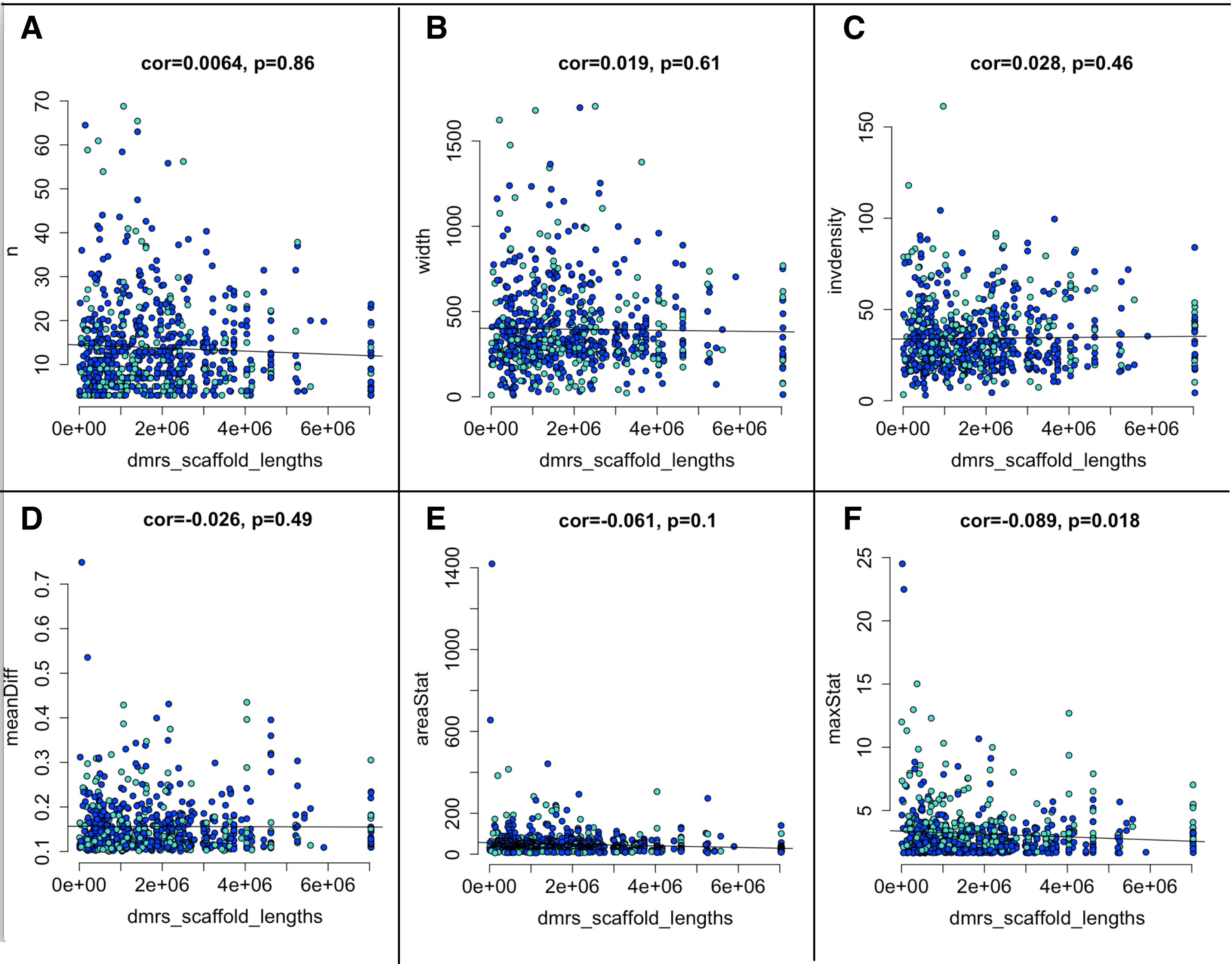

A
cor=0.0064, p=0.86
n
dmrs_scaffold_lengths
B
cor=0.019, p=0.61
width
dmrs_scaffold_lengths
C
cor=0.028, p=0.46
invdensity
dmrs_scaffold_lengths
D
cor=-0.026, p=0.49
meanDiff
dmrs_scaffold_lengths
E
cor=-0.061, p=0.1
areaStat
dmrs_scaffold_lengths
F
cor=-0.089, p=0.018
maxStat
dmrs_scaffold_lengths
